# Design, synthesis, anticancer evaluation, and molecular modelling studies of novel tolmetin derivatives as potential VEGFR-2 inhibitors and apoptosis inducers

**DOI:** 10.1080/14756366.2021.1901089

**Published:** 2021-04-26

**Authors:** Asmaa E. Kassab, Ehab M. Gedawy, Mohammed I. A. Hamed, Ahmed S. Doghish, Rasha A. Hassan

**Affiliations:** a Faculty of Pharmacy, Department of Pharmaceutical Organic Chemistry, Cairo University, Cairo, Egypt; b Faculty of Pharmacy, Department of Pharmaceutical Chemistry, Badr University in Cairo (BUC), Badr City, Egypt; c Faculty of Pharmacy, Department of Organic and Medicinal Chemistry, Fayoum University, Fayoum, Egypt; d Faculty of Pharmacy (Boys), Department of Biochemistry, Al-Azhar University, Nasr City, Cairo, Egypt; e Faculty of Pharmacy, Department of Biochemistry, Badr University in Cairo (BUC), Badr City, Egypt

**Keywords:** Tolmetin, synthesis, anticancer activity, VEGFR-2, apoptosis

## Abstract

Novel tolmetin derivatives **5a–f** to **8a–c** were designed, synthesised, and evaluated for antiproliferative activity by NCI (USA) against a panel of 60 tumour cell lines. The cytotoxic activity of the most active tolmetin derivatives **5b** and **5c** was examined against HL-60, HCT-15, and UO-31 tumour cell lines. Compound **5b** was found to be the most potent derivative against HL-60, HCT-15, and UO-31 cell lines with IC_50_ values of 10.32 ± 0.55, 6.62 ± 0.35, and 7.69 ± 0.41 µM, respectively. Molecular modelling studies of derivative **5b** towards the VEGFR-2 active site were performed. Compound **5b** displayed high inhibitory activity against VEGFR-2 (IC_50_ = 0.20 µM). It extremely reduced the HUVECs migration potential exhibiting deeply reduced wound healing patterns after 72 h. It induced apoptosis in HCT-15 cells (52.72-fold). This evidence was supported by an increase in the level of apoptotic caspases-3, -8, and -9 by 7.808-, 1.867-, and 7.622-fold, respectively. Compound **5b** arrested the cell cycle in the G0/G1 phase. Furthermore, the ADME studies showed that compound **5b** possessed promising pharmacokinetic properties.

## Introduction

Cancer is a dangerous life-threatening disease which considered the second cause of death after cardiovascular diseases^1^. Although it started long ago, the search for effective and safer new antitumor drugs is still an active research topic due to the systemic toxicity caused by classical non-selective chemotherapies and resistance development for the existing anticancer drugs^2^. Therefore, it remains an urgent research point to develop new antitumor agents with improved tumour selectivity, efficiency, and safety.

**Figure 1.**
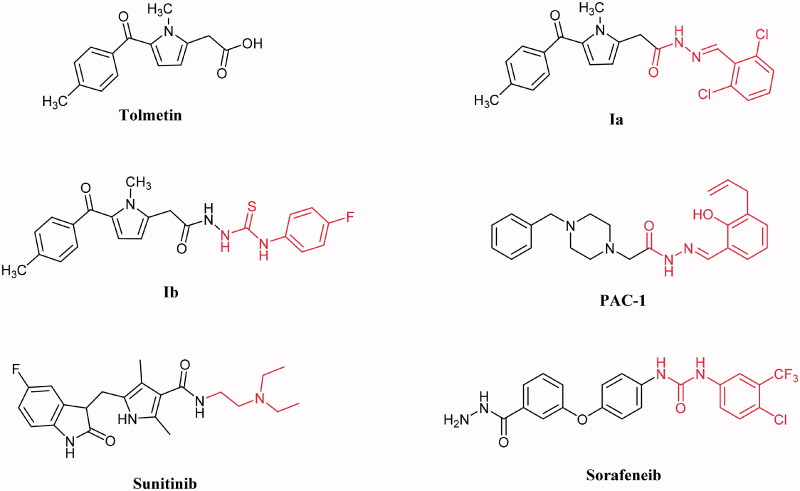
The biological active apoptosis inducers and VEGFR-2 inhibitors.

One of the hallmarks of cancer is the ability of cancer cells to avoid apoptosis, permitting unchecked proliferation^3^. Therefore, reactivation of apoptosis in cells with defective apoptotic pathways is a promising anticancer strategy. Compounds such as Bcl-2 inhibitors (ABT-737), XIAP inhibitors (SM-164), and p53-MDM2 disruptors (Nutlins), act specifically on proteins within the apoptotic cascade, actuating apoptosis and driving to death of cancer cells^4–6^. Although tolmetin showed minor activity, tolmetin hydrazone and thiosemicarbazide derivatives **Ia** and **Ib** (Figure 1) exhibited anticancer activity against colon (HT-29) and prostate (PC-3) cancer cell lines, respectively, due to the activation of caspase-8 and caspase-9 involved in the apoptotic pathway^7^^,^^8^. Moreover, procaspase-3 levels are elevated in certain cancers, including lymphomas, leukemias, melanomas, pancreatic, liver, lung, breast, and colon cancers^9–14^. Procaspase-Activating Compound-1 (PAC-1, Figure 1) reinforces the enzymatic activity of procaspase-3 *in vitro*, induces apoptotic cell death in cancer cells^15^. Structure-activity relationship (SAR) studies revealed that the activity of PAC-1 *in vitro* and in cell culture is dependent on the presence of the *o*-hydroxy *N*-acyl hydrazone moiety (highlighted in Figure 1), a structural motif known to participate in Zn^2+^ metal chelation^16^^,^^17^.

On the other side, angiogenesis is essential for the rapid expansion of a tumour mass and is a critical process for the formation and metastases^18^. Numerous growth factors are involved in angiogenesis, the vascular endothelial growth factor (VEGF) is one of the most known angiogenic molecules which regulate blood and lymphatic vessel development and homeostasis^19^. The biological action of VEGF is interceded by three structurally related receptors, VEGFR-1 (Flt-1), VEGFR-2 (Flk-1/Kinase domain receptor), and VEGFR-3 (Flt-4)^20^. VEGFR-2 is the major regulator of VEGF-driven responses in endothelial cells and can mediate proliferation, differentiation, and microvascular permeability. Moreover, it has been confirmed to be a prerequisite signal transducer in both pathologic and physiologic angiogenesis^21^^,^^22^. VEGFR-2 is overexpressed in several malignancies, including hepatocellular carcinoma, ovarian, colorectal, breast, thyroid, melanoma, and medulloblastoma cancers^23–25^. Subsequently, suppression of angiogenesis through blocking the VEGFR-2 pathway has been recognised as an amazing medicinal target for the generation of novel anticancer agents. Besides, prohibition of VEGF signalling can also alter or annihilate tumour vessels^26^.

Several VEGFR-2 kinase inhibitors have been approved as anti-angiogenic drugs. Generally, VEGFR-2 inhibitors are classified into three categories, Type I inhibitors such as sunitinib (Figure 1), competitively bind to the ATP binding pocket in the active “DFG-in” conformation via hydrophobic interactions in and around the adenine region and hydrogen bonding with the hinge region^27^. Type II inhibitors such as sorafenib (Figure 1), occupy the ATP binding site in the inactive “DFG-out” conformation and extend over the gatekeeper Val916 into the adjacent allosteric hydrophobic back pocket^27–30^. Type III inhibitors bind to the inactive “DFG-out” conformation beyond the gatekeeper Val916 especially to the less conservative allosteric hydrophobic back pocket outside the adenosine triphosphate (ATP)-binding pocket locking VEGFR-2 in the inactive “DFG-out” conformation. Subsequently, they are anticipated to own superior selectivity profiles and provide new opportunities for scaffold development^27^^,^^30^.

Sunitinib was approved by FDA as a multikinase inhibitor for the treatment of patients with progressed renal cell carcinoma, gastrointestinal stromal tumour, and chronic myeloid leukaemia^31^^,^^32^. Sunitinib also induces apoptosis and inhibits cell proliferation of human medulloblastomas owing to activation of caspase-3, caspase-9, and upregulation of pro-apoptotic genes (Bak and Bim), and restrains the expression of survivin^33^^,^^34^. However, some adverse effects are observed during its clinical use, such as hypothyroidism, hypertension, cardiotoxicity, left ventricular dysfunction and overt heart failure, fatigue, hand-foot syndrome, diarrhoea^35^^,^^36^, indicating that the development of safer VEGFR-2 inhibitors remains an active field of research.

A study of the SAR and binding pattern of sunitinib and various VEGFR-2 inhibitors uncovered that they shared four fundamental features^37^: (i) The core structure of most inhibitors consists of a flat aromatic ring system which occupied the catalytic ATP-binding domain and participating in H-bond interaction with Cys919 residues located in the hinge area of the binding site, sunitinib can form an extra hydrogen bond with the amino acid of the hinge region as Glu917^20^^,^^38^. (ii) A central aryl ring (hydrophobic spacer) occupies the connecting area between the ATP-binding domain and the DFG domain^39^. (iii) Most VEGFR-2 inhibitors as in sorafenib apparently form H-bond interactions through a linker containing a functional group acting as pharmacophore (e.g. amino or urea) with two essential residues (Glu885 and Asp1046) in the DFG (Asp-Phe-Gly) motif^40^. (iv) The terminal hydrophobic moiety that occupies the allosteric hydrophobic pocket through various hydrophobic interactions^40^.

**Figure 2.**
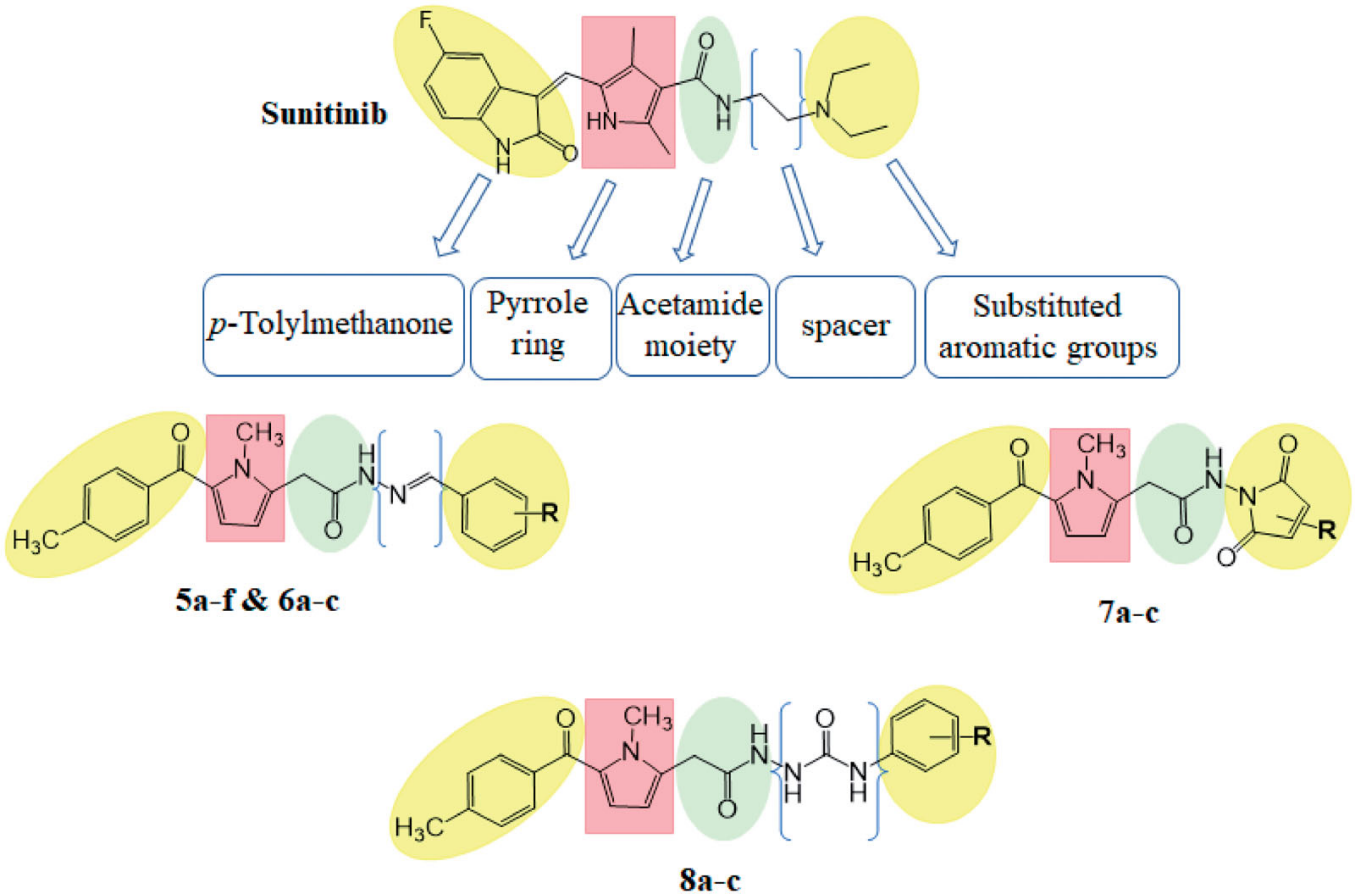
Structural similarities and pharmacophoric features of VEGFR-2 inhibitor (sunitinib) and designed compounds.

The goal of our work is to synthesise new tolmetin derivatives with the same essential pharmacophoric features of the reported and clinically used sunitinib (through molecular hybridisation with the essential moieties of PAC-1, sorafenib or cyclisation of *N*,*N*-diethylamine moiety of sunitinib, highlighted in Figure 2), aiming the development of efficient anticancer agents that exhibited different enzymatic and cellular targets such as apoptosis induction in different human cancer cell lines and inhibition of VEGFR-2.

Firstly, a bioisosteric approach was adopted in the target *p*-tolylmethanone to replace indolin-2-one moiety with the purpose of sharing H-bond with amino acid Cys919 (Figure 2), type I inhibitors typically form one to three hydrogen bonds with Cys919 and Glu917 residues in the hinge simulating the adenine of ATP^20^. The 2nd strategy is using an acetamide linker containing HBA-HBD functional groups that possess H-bond acceptors and/or donors. The 3rd strategy involves the replacement of the ethyl spacer between the amide and *N*,*N*-diethylamine moieties of sunitinib by azomethine, urea or removing it, to study its effect on fitting the terminal hydrophobic group with the allosteric binding domain. Within the 4th strategy, the hydrophobic *N*,*N*-diethylamine tail of sunitinib was replaced by other distal distinctive hydrophobic moieties including substituted phenyl, aromatic heterocyclic or alicyclic groups. The substitution design of these hydrophobic moieties was selected to guarantee diverse lipophilic and electronic environments, which might impact the activity of the target compounds. Besides, the hydrophobic moieties were further modified to carry additional HBA-HBD groups that will result in various extra binding interactions within the pocket leading to diverse inhibitory activity.

In this study, all the synthesised compounds were evaluated for their *in vitro* antiproliferative activities on a panel of 60 tumour cell lines. The results prompted more examinations to gain a thorough understanding of the mechanism of action of the synthesised compounds. The most active cytotoxic compounds were selected to be further studied through the determination of their half-maximal inhibitory concentration (IC_50_) values against three cancer cell lines (HL-60, HCT-15, and UO-31). The most potent compound **5b**, was subjected to further investigation for its kinase inhibitory activity against VEGFR-2, and wound healing assay was performed to examine its *in vitro* anti-angiogenic property. Analysis of its conceivable influence on cell cycle progression and apoptosis induction possibility in HCT-15 cell line was carried out. Molecular docking studies were conducted to study the interaction of compound **5b** with VEGFR-2 active site, its binding mode, and the ability to satisfy the pharmacophoric features required to induce the desired inhibition.

## Materials and methods

### Chemistry

#### General

Sigma-Aldrich Company is the only supplier for all the used chemicals. Melting points were obtained on a Griffin apparatus and were uncorrected. Microanalyses for C, H, and N were carried out at the Regional Centre for Mycology and Biotechnology, Faculty of Pharmacy, Al-Azhar University. IR spectra were recorded on Shimadzu IR 435 spectrophotometer (Shimadzu Corp., Kyoto, Japan) Faculty of Pharmacy, Cairo University, Cairo, Egypt, and values were represented in cm^−1^. ^1^H NMR spectra were carried out on Bruker 400 MHz (Bruker Corp., Billerica, MA, USA) spectrophotometer, Faculty of Pharmacy, Cairo University, Cairo, Egypt. Chemical shifts were recorded in ppm on *δ* scale, coupling constants (*J*) were given in Hz, and peak multiplicities are designed as follows: s, singlet; d, doublet; dd, doublet of doublet; t, triplet; m, multiplet. ^13^C NMR spectra were carried out on Bruker 100 MHz spectrophotometer, Faculty of Pharmacy, Cairo University, Cairo, Egypt. Progress of the reactions was monitored by TLC using precoated aluminium sheet silica gel MERCK 60 F 254 and was visualised by UV lamp.

2–(1-Methyl-5–(4-methylbenzoyl)-1*H*-pyrrol-2-yl)acetic acid (tolmetin) (**2**), methyl 2–(1-methyl-5–(4-methylbenzoyl)-1*H*-pyrrol-2-yl)acetate (**3**) and 2–(1-methyl-5–(4-methylbenzoyl)-1*H*-pyrrol-2-yl)acetohydrazide (**4**) were prepared according to the reported procedures^7^.

#### General procedure for the synthesis of tolmetin hydrazones (5a–f)

A mixture of tolmetin hydrazide **4** (2.71 g, 0.01 mol) and the appropriate aldehyde (0.01 mol) was dissolved in absolute ethanol containing 0.5 ml of glacial acetic acid. The reaction mixture was refluxed for 6 h. After cooling the separated solid was filtered, washed with ethanol, and crystallised from ethanol.

##### N′-(2-Hydroxybenzylidene)-2–(1-methyl-5–(4-methylbenzoyl)-1H-pyrrol-2-yl)aceto hydrazide (5a)

White solid: 85% yield; mp 222–224 °C; IR (KBr, cm^−1^) 3200–3100 (br, OH, NH str), 3028 (CH aromatic), 2990, 2954 (CH aliphatic), 1685 (C=O str), 1604 (C=N str), 1273, 748; ^1^H NMR (400 MHz, DMSO-d_6_) *δ*: 11.88, 11.50 (2s, 1H, OH, D_2_O exchangeable), 11.13, 11.04 (2s, 1H, OH/NH, D_2_O exchangeable), 8.43, 8.34 (2s, 1H, N = CH), 7.73–7.69, 7.55–7.53 (2 m, 1H, ArH), 7.63 (dd, *J* = 2.4, 8.4 Hz, 2H, ArH), 7.31 (dd, *J* = 3.2, 8.4 Hz, 2H, ArH), 7.27–7.23 & 7.00–6.96 (2 m, 1H, ArH), 6.93–6.85 (m, 2H, ArH) 6.59 (t, *J* = 4.4 Hz, 1H, C4H-pyrrole), 6.15, 6.12 (2d, *J* = 4.4, 4.4 Hz, 1H, C3H-pyrrole), 4.16, 3.78 (2s, 2H, CH_2_C=O) 3.90, 3.87 (2s, 3H, N-CH_3_), 2.39 (s, 3H, ArCH_3_); ^13^C NMR (100 MHz, DMSO-d_6_) *δ*: 184.8, 184.7, 170.3, 164.9, 157.7, 156.8, 147.5, 142.0, 141.8, 141.6, 137.7, 137.5, 137.4, 137.1, 131.8, 131.6, 130.8, 130.6, 129.7, 129.4, 129.3, 129.2, 129.1, 122.2, 122.1, 120.5, 119.9, 119.8, 119.0, 116.8, 116.6, 110.0, 109.8, 33.5, 33.4, 32.8, 31.2, 21.5; Anal. Calcd. for C_22_H_21_N_3_O_3_ (375.42): C, 70.38; H, 5.64; N, 11.19 found C, 70.54; H, 5.80; N, 11.51.

##### N′-(2,3-Dihydroxybenzylidene)-2–(1-methyl-5–(4-methylbenzoyl)-1H-pyrrol-2-yl) acetohydrazide (5b)

Creamy white solid: 90% yield; mp 263–265 °C; IR (KBr, cm^−1^) 3471 (NH str), 3151 (OH str), 3028 (CH aromatic), 2989, 2954 (CH aliphatic), 1685 (C=O str), 1604 (C=N str), 1289, 740; ^1^H NMR (400 MHz, DMSO-d_6_) *δ*: 11.92, 11.50 (2s, 1H, OH/NH, D_2_O exchangeable), 10.84 (s, 1H, OH, D_2_O exchangeable), 9.27 (s, 1H, OH, D_2_O exchangeable), 8.38, 8.33 (2s, 1H, N = CH), 7.65–7.62 (m, 1H, ArH), 7.33–7.30 (m, 2H, ArH), 7.17–7.15 & 6.98–6.95 (2 m, 1H, ArH), 6.86–6.81 (m, 1H, ArH), 6.75–6.67 (m, 1H, ArH), 6.75–6.67 (m, 1H, ArH), 6.59, 6.57 (2d, *J* = 4.0, 4.0 Hz, 1H, C4H-pyrrole), 6.15, 6.11 (2d, *J* = 4.0, 4.0 Hz, 1H, C3H-pyrrole), 4.15, 3.79 (2s, 2H, CH_2_C=O), 3.90, 3.87 (2s, 3H, N-CH_3_), 2.39 (s, 3H, ArCH_3_); ^13^C NMR (100 MHz, DMSO-d_6_) *δ*: 184.8, 184.7, 170.2, 164.9, 148.3, 146.4, 146.1, 146.0, 145.7, 142.3, 141.9,141.8, 137.7, 137.5, 137.4, 137.1, 130.8, 130.6, 129.4, 129.3, 129.1, 129.1, 122.2, 122.1, 121.0, 120.2, 119.6, 119.1, 117.8, 117.3, 117.0, 110.0, 109.8, 33.5, 33.4, 32.7, 31.2 21.5; Anal. Calcd. for C_22_H_21_N_3_O_4_ (391.42): C, 67.51; H, 5.41; N, 10.74, found C, 67.80; H, 5.62; N, 11.02.

##### N′-(2,4-Dihydroxybenzylidene)-2–(1-methyl-5–(4-methylbenzoyl)-1H-pyrrol-2-yl) acetohydrazide (5c)

Light brown solid: 85% yield; mp 226–228 °C; IR (KBr, cm^−1^) 3300–3100 (br, 2OH, NH str), 3032 (CH aromatic), 2985, 2954 (CH aliphatic), 1670 (C=O str), 1631, 1608 (C=N str), 1265, 748; ^1^H NMR (400 MHz, DMSO-d_6_) *δ*: 11.69, 11.40 (2s, 1H, OH, D_2_O exchangeable), 11.31, 11.20 (2s, 1H, OH/NH, D_2_O exchangeable), 9.95, 9.81 (2s, 1H, OH, D_2_O exchangeable), 8.29, 8.20 (2s, 1H, N = CH), 7.63 (d, *J* = 8.0 Hz, 2H, ArH), 7.49, 7.43 (2d, *J* = 8.4, 8.4 Hz, 1H, ArH), 7.31 (d, *J* = 8.0 Hz, 2H, ArH), 6.59–6.57 (m, 1H, C4H-pyrrole), 6.36–6.30 (m, 2H, ArH), 6.13, 6.10 (2d, *J* = 4.0, 4.0 Hz 1H, C3H-pyrrole), 4.11, 3.74 (2s, 2H, CH_2_C=O) 3.90, 3.86 (2s, 3H, N-CH_3_), 2.39 (s, 3H, ArCH_3_); ^13^C NMR (100 MHz, DMSO-d_6_) *δ*: 184.8, 184.7, 169.8, 164.5, 161.1, 160.8, 159.7, 158.5, 148.5, 142.7, 141.9, 141.8, 137.8, 137.5, 137.4, 137.3, 131.6, 130.7, 130.6, 129.4, 129.3, 129.2, 129.1, 122.2, 122.1, 112.0, 110.8, 110.7, 109.9, 109.7, 108.1, 103.0, 33.5, 33.4, 32.8, 31.2, 21.5; Anal. Calcd. for C_22_H_21_N_3_O_4_ (391.42): C, 67.51; H, 5.41; N, 10.74, found C, 67.62; H, 5.65; N, 10.98.

##### N′-(Benzo[d][1,3]dioxol-5-ylmethylene)-2–(1-methyl-5–(4-methylbenzoyl)-1H-pyrrol-2-yl)acetohydrazide (5d)

Creamy white solid: 88% yield; mp 242–244 °C; IR (KBr, cm^−1^) 3417 (NH str), 3093 (CH aromatic), 2974, 2912 (CH aliphatic), 1670 (C=O str), 1624, 1604 (C=N str), 1265, 744; ^1^H NMR (400 MHz, DMSO-d_6_) *δ*: 11.57, 11.45 (2s, 1H, OH/NH, D_2_O exchangeable), 8.14, 7.95 (2s, 1H, N = CH), 7.63 (d, *J* = 8.0 Hz, 2H, ArH), 7.35, 7.25 (2d, *J* = 1.6, 1.6 Hz, 1H, ArH), 7.31 (d, *J* = 8.0 Hz, 2H, ArH), 7.16, 7.13 (2dd, *J* = 1.6, 8.0 & 1.6, 8.0 Hz, 1H, ArH), 6.98, 6.97 (2d, *J* = 8.0, 8.0 Hz, 1H, ArH), 6.59–6.57 (m,1H, C4H-pyrrole), 6.11 (d, *J* = 4.0 Hz, 1H, C3H-pyrrole), 6.08 (s, 2H, OCH_2_O), 4.17, 3.73 (2s, 2H, CH_2_C=O) 3.90, 3.87 (2s, 3H, N-CH_3_), 2.39 (s, 3H, ArCH_3_); ^13^C NMR (100 MHz, DMSO-d_6_) *δ*: 184.8, 184.7, 170.5, 164.9, 161.1, 149.3, 148.4, 147.1, 143.6, 141.8,137.9, 137.5, 137.4, 130.7, 130.6, 129.4, 129.3, 129.1, 129.3, 129.0, 128.8, 125.5, 123.7, 123.5, 122.2, 122.1, 110.0, 109.7, 109.0, 108.8, 106.4, 105.6, 105.5, 102.1, 102.0, 101.9, 33.4, 33.4, 33.0, 31.1, 21.5; Anal. Calcd. for C_23_H_21_N_3_O_4_ (403.43): C, 68.47; H, 5.25; N, 10.42. found C, 68.71; H, 5.44; N, 10.69

##### N′-((1H-Indol-2-yl)methylene)-2–(1-methyl-5–(4-methylbenzoyl)-1H-pyrrol-2-yl) acetohydrazide (5e)

Reddish brown solid, 84% yield; mp 198–200 °C; IR (KBr, cm^−1^) 3313, 3205 (2 NH str), 3055 (CH aromatic), 2951, 2920 (CH aliphatic), 1666 (C=O str), 1604 (C=N str), 1265, 738; ^1^H NMR (400 MHz, DMSO-d_6_) *δ*: 11.68, 11.59 (2s, 1H, NH, D_2_O exchangeable), 11.56, 11.44 (2s, 1H, OH/NH, D_2_O exchangeable), 8.29, 8.09 (2s, 1H, N = CH), 7.65 (d, *J* = 8.0 Hz, 2H, ArH), 7.57, 7.56 (2d, *J* = 8.0, 8.0 Hz, 1H, ArH), 7.45 (d, *J* = 8.0 Hz, 1H, ArH), 7.30 (d, *J* = 8.0 Hz, 2H, ArH), 7.21–7.14 (m, 1H, ArH), 7.05–6.99 (m, 1H, ArH), 6.84, 6.81 (2d, *J* = 2.4, 2.4 Hz, 1H, ArH), 6.60 (d, *J* = 4.4, Hz, 1H, C4H-pyrrole), 6.19, 6.17 (2d, *J* = 4.0, 4.0 Hz, 1H, C3H-pyrrole), 4.31, 3.80 (2s, 2H, CH_2_C=O) 3.93, 3.92 (2s, 3H, N-CH_3_), 2.38 (s, 3H, ArCH_3_); ^13^C NMR (100 MHz, DMSO-d_6_) *δ*: 184.8, 184.7, 170.7, 164.9, 141.9, 141.8, 137.9, 137.9, 137.5, 137.4, 136.2, 133.7, 133.4, 130.8, 130.7, 129.4, 129.4, 129.1, 129.1, 128.2, 128.0, 123.8, 122.2, 122.2, 121.3, 121.2, 120.0, 112.4, 112.0, 110.2, 109.8, 107.4, 106.5, 33.5, 33.5, 33.1, 30.9, 21.4; Anal. Calcd. for C_24_H_22_N_4_O_2_ (398.46): C, 72.34; H, 5.57; N, 14.06, found C, 72.60; H, 5.71; N, 14.27.

##### N′-((1H-Indol-3-yl)methylene)-2–(1-methyl-5–(4-methylbenzoyl)-1H-pyrrol-2-yl) acetohydrazide (5f)

Creamy white solid: 85% yield; mp 168–170 °C; IR (KBr, cm^−1^) 3417, 3294 (2 NH str), 3078 (CH aromatic), 2958, 2924 (CH aliphatic), 1666 (C=O str), 1593 (C=N str), 1273, 740; ^1^H NMR (400 MHz, DMSO-d_6_) *δ*: 11.56 (s, 1H, NH, D_2_O exchangeable), 11.32, 11.24 (2s, 1H, OH/NH, D_2_O exchangeable), 8.39, 8.23 (2s, 1H, N = CH), 8.20, 8.13 (2d, *J* = 8.0, 8.0 Hz, 1H, ArH), 7.81–7.80 (m, 1H, ArH), 7.65–7.62 (m, 2H, ArH), 7.45–7.42 (m, 1H, ArH), 7.32–7.30 (m, 2H, ArH), 7.22–7.18 (m, 1H, ArH), 7.15–7.10 (m, 1H, ArH), 6.59 (t, *J* = 4.0, Hz, 1H, C4H-pyrrole), 6.15 (d, *J* = 4.0 Hz, 1H, C3H-pyrrole), 4.22, 3.73 (2s, 2H, CH_2_C=O) 3.94, 3.91 (2s, 3H, N-CH_3_), 2.39 (s, 3H, ArCH_3_); ^13^C NMR (100 MHz, DMSO-d_6_) *δ*: 184.8, 184.7, 169.8, 164.2, 144.4, 141.9, 141.8, 141.3, 138.2, 137.9, 137.5, 137.5, 137.4, 130.8, 130.5, 129.4, 129.3, 129.1, 124.5, 123.0, 122.2, 122.0, 121.0, 120.8, 112.3, 111.9, 111.8, 110.0, 109.6, 33.5, 33.4, 33.1, 31.3, 21.5; Anal. Calcd. for C_24_H_22_N_4_O_2_ (398.46): C, 72.34; H, 5.57; N, 14.06, found C, 72.59; H, 5.81; N, 14.32.

#### General procedure for the preparation of tolmetin hydrazones (6a–c)

A mixture of tolmetin hydrazide **4** (0.01 mol) and isatin or 5-substituted isatin (0.01 mol) were dissolved in absolute ethanol containing 0.5 ml of glacial acetic acid. The reaction mixture was refluxed for 6 h. After cooling the separated solid was filtered, washed with ethanol and crystallised from ethanol.

##### 2–(1-Methyl-5–(4-methylbenzoyl)-1H-pyrrol-2-yl)-N′-(2-oxoindolin-3-ylidene) acetohydrazide (6a)

Yellow solid: 90% yield; mp 193–195 °C; IR (KBr, cm^−1^) 3200–3155 (br, 2 NH str), 3081 (CH aromatic), 2954, 2920 (CH aliphatic), 1693 (br, C=O str), 1624 (C=N str), 1265, 744; ^1^H NMR (400 MHz, DMSO-d_6_) *δ*: 13.00, 12.65 (2s, 1H, OH/NH, D_2_O exchangeable), 11.27 (s, 1H, NH, D_2_O exchangeable), 7.65–7.58 (m, 3H, ArH), 7.39 (t, *J* = 7.6 Hz, 1H, ArH), 7.31 (d, *J* = 8.0 Hz, 2H, ArH), 7.11 (t, *J* = 7.6 Hz, 1H, ArH), 6.97–6.92 (m, 1H, ArH), 6.60 (d, *J* = 4.0, Hz, 1H, C4H-pyrrole), 6.19 (d, *J* = 4.0 Hz, 1H, C3H-pyrrole), 4.35, 4.07 (2s, 2H, CH_2_C=O) 3.88 (s, 3H, N-CH_3_), 2.39 (s, 3H, ArCH_3_); ^13^C NMR (100 MHz, DMSO-d_6_) *δ*: 184.9, 171.7, 162.9, 142.9, 142.0, 137.4, 132.1, 129.4, 129.2, 123.1, 122.2, 120.1, 111.6, 110.3, 33.4, 30.1, 21.5; Anal. Calcd. for C_23_H_20_N_4_O_3_ (400.43): C, 68.99; H, 5.03; N, 13.99, found C, 69.21; H, 5.18; N, 14.24

##### N′-(5-Bromo-2-oxoindolin-3-ylidene)-2–(1-methyl-5–(4-methylbenzoyl)-1H-pyrrol-2-yl)acetohydrazide (6b)

Yellow solid: 83% yield; mp 195–197 °C; IR (KBr, cm^−1^) 3200–3130 (br, 2 NH str), 3050 (CH aromatic), 2993, 2927 (CH aliphatic), 1697 (br, C=O str), 1600 (C=N str), 1265, 748; ^1^H NMR (400 MHz, DMSO-d_6_) *δ*: 12.93, 12.56 (2s, 1H, OH/NH, D_2_O exchangeable), 11.38 (s, 1H, NH, D_2_O exchangeable), 7.79–7.71 (m, 1H, ArH), 7.64 (d, *J* = 8.0 Hz, 2H, ArH), 7.55 (dd, *J* = 2.0, 8.4 Hz, 1H, ArH), 7.32 (d, *J* = 8.0 Hz, 2H, ArH), 6.92 (d, *J* = 8.4 Hz, 1H, ArH), 6.60 (d, *J* = 4.0, Hz, 1H, C4H-pyrrole), 6.19 (d, *J* = 4.0 Hz, 1H, C3H-pyrrole), 4.37, 4.13 (2s, 2H, CH_2_C=O), 3.87 (s, 3H, N-CH_3_), 2.39 (s, 3H, ArCH_3_); ^13^C NMR (100 MHz, DMSO-d_6_) *δ*: 184.9, 171.7, 162.5, 141.9, 137.4, 136.2, 134.2, 130.8, 129.4, 129.1, 123.6, 122.3, 122.1, 114.8, 113.6, 110.3, 33.4, 30.2, 21.5; Anal. Calcd. for C_23_H_19_BrN_4_O_3_ (479.33): C, 57.63; H, 4.00; N, 11.69, found C, 57.85; H, 4.23; N, 11.91.

##### 2–(1-Methyl-5–(4-methylbenzoyl)-1H-pyrrol-2-yl)-N′-(2-oxo-5-(trifluoromethoxy) indolin-3-ylidene)acetohydrazide (6c)

Yellow solid: 90% yield; mp 157–159 °C; IR (KBr, cm^−1^) 3300–3200 (br, 2 NH str), 3055 (CH aromatic), 2951, 2924 (CH aliphatic), 1697 (br, C=O str), 1604 (C=N str), 1265, 748; ^1^H NMR (400 MHz, DMSO-d_6_) *δ*: 12.94, 12.58 (2s, 1H, OH/NH, D_2_O exchangeable), 11.44 (s, 1H, NH, D_2_O exchangeable), 7.64 (d, *J* = 8.0 Hz, 2H, ArH), 7.53 (s, 1H, ArH), 7.40 (dd, *J* = 2.0, 8.4 Hz, 1H, ArH), 7.31 (d, *J* = 8.0 Hz, 2H, ArH), 7.04 (d, *J* = 8.8 Hz, 1H, ArH), 6.60 (d, *J* = 4.0, Hz, 1H, C4H-pyrrole), 6.19 (d, *J* = 4.0 Hz, 1H, C3H-pyrrole), 4.38, 4.12 (2s, 2H, CH_2_C=O) 3.87 (s, 3H, N-CH_3_), 2.40 (s, 3H, ArCH_3_); ^13^C NMR (100 MHz, DMSO-d_6_) *δ*: 184.9, 172.9, 163.0, 144.1, 142.0 141.8, 137.4, 129.4, 129.1, 125.0, 122.1, 121.9, 121.5, 119.3, 114.5, 112.7, 110.3, 33.4, 30.3, 21.5; Anal. Calcd. for C_24_H_19_F_3_N_4_O_4_ (484.43): C, 59.50; H, 3.95; N, 11.57, found C, 59.76; H, 4.12; N, 11.80

#### General procedure for the synthesis of tolmetin derivatives (7a–c)

Equimolar amounts of tolmetin hydrazide **4** (2.71 g, 0.01 mol) and the selected acid anhydride and anhydrous sodium acetate (0.082 g, 0.001 mol) were dissolved in glacial acetic acid (10 ml) and heated under reflux for 12 h. After cooling the separated solid was filtered, dried, and crystallised from ethanol.

##### N-(2,5-Dioxopyrrolidin-1-yl)-2–(1-methyl-5–(4-methylbenzoyl)-1H-pyrrol-2-yl) acetamide (7a)

Brown solid: 86% yield; mp >300 °C; IR (KBr, cm^−1^) 3194 (NH str), 3032 (CH aromatic), 2947, 2870 (CH aliphatic), 1720, 1712 (C=O str), 1269, 748; ^1^H NMR (400 MHz, DMSO-d_6_) *δ*: 9.86, 9.69 (2s, 1H, OH/NH, D_2_O exchangeable), 7.62 (d, *J* = 7.4 Hz, 2H, ArH), 7.31 (d, *J* = 7.4 Hz, 2H, ArH), 6.56 (d, *J* = 4.0, Hz, 1H, C4H-pyrrole), 6.13 (d, *J* = 4.0 Hz, 1H, C3H-pyrrole), 3.88 (s, 3H, N-CH_3_), 3.67 (s, 2H, CH_2_C=O), 2.39 (s, 3H, ArCH_3_), 1.87 (s, 2H, CH_2_), 1.84 (s, 2H, CH_2_); ^13^C NMR (100 MHz, DMSO-d_6_) *δ*: 184.9, 175.7, 168.6, 168.4, 167.5, 141.9, 137.5, 137.4, 130.7, 129.4, 129.1, 122.1, 33.3, 32.2, 32.1, 21.5, 20.9; Anal. Calcd. for C_19_H_19_N_3_O_4_ (353.37): C, 64.58; H, 5.42; N, 11.89, found C, 64.82; H, 5.66; N, 12.11

##### N-(2,5-Dioxo-2,5-dihydro-1H-pyrrol-1-yl)-2–(1-methyl-5–(4-methylbenzoyl)-1H-pyrrol-2-yl)acetamide (7b)

Light brown solid, 85% yield; mp 193–195 °C; IR (KBr, cm^−1^) 3356 (NH str), 3032 (CH aromatic), 2900, 2951 (CH aliphatic), 1720–1670 (br C=O str), 1265, 748; ^1^H NMR (400 MHz, DMSO-d_6_) *δ*: 10.04, 9.84 (2s, 1H, OH/NH, D_2_O exchangeable), 7.62 (d, *J* = 8.4 Hz, 2H, ArH), 7.31 (d, *J* = 8.4 Hz, 2H, ArH), 6.58–6.55 (m, 1H, C4H-pyrrole), 6.13 (d, *J* = 4.4 Hz 1H, C3H-pyrrole), 6.03 (s, 2H, pyrrole), 3.85 (s, 3H, N-CH_3_), 3.66 (s, 2H, CH_2_C=O), 2.39 (s, 3H, ArCH_3_); ^13^C NMR (100 MHz, DMSO-d_6_) *δ*: 184.9, 168.6, 167.9, 142.0, 137.4, 137.4, 136.5, 130.6, 129.4, 129.2, 122.2, 109.8, 33.3, 32.1, 21.4; Anal. Calcd. for C_19_H_17_N_3_O_4_ (351.36): C, 64.95; H, 4.88; N, 11.96, found C, 65.12; H, 5.14; N, 12.09.

##### N-(1,3-Dioxoisoindolin-2-yl)-2–(1-methyl-5–(4-methylbenzoyl)-1H-pyrrol-2-yl) acetamide (7c)

Light brown solid, 85% yield; mp >300 °C; IR (KBr, cm^−1^) 3197 (NH str), 3024 (CH aromatic), 2951, 2885 (CH aliphatic), 1743, 1670 (C=O str), 1265, 748; ^1^H NMR (400 MHz, DMSO-d_6_): *δ*: 8.14 (s, 1H, ArH), 7.89 (d, *J* = 2.0 Hz, 2H, ArH), 7.62 (dd, *J* = 2.0, 8.0 Hz, 2H, ArH), 7.49–7.47 (m, 1H, ArH), 7.31 (d, *J* = 8.0 Hz, 2H, ArH), 6.57 (d, *J* = 4.0 Hz, 1H, C4H-pyrrole), 6.18, 6.13 (2d, *J* = 4.0, 4.0 Hz, 1H, C3H-pyrrole), 3.93 (s, 3H, N-CH_3_), 3.81 (s, 2H, CH_2_C=O), 3.66 (s, 1H, NH, D_2_O exchangeable), 2.39 (s, 3H, ArCH_3_); ^13^C NMR (100 MHz, DMSO-d_6_) *δ*:184.9, 168.7, 165.6, 142.0, 137.4, 136.9, 135.6, 130.8, 130.4, 129.4, 129.1, 124.0, 122.1, 109.9, 33.3, 32.1, 21.5; Anal. Calcd. for C_23_H_19_N_3_O_4_ (401.41): C, 68.82; H, 4.77; N, 10.47, found C, 69.03; H, 5.01; N, 10.75.

#### General procedure for the synthesis of tolmetin semicarbazide derivatives (8a–c)

A solution of tolmetin hydrazide **4** (0.81 g, 0.003 mol) in methylene chloride (5 ml) at 0 °C was treated with the appropriate aryl isocyanate (0.0033 mol) and stirred overnight at room temperature. To the resulting suspension, hexane was added to complete precipitation. The solid was filtered, dried, and crystallised from ethanol.

##### N-(2,6-Dichlorophenyl)-2–(2-(1-methyl-5–(4-methylbenzoyl)-1H-pyrrol-2-yl)acetyl) hydrazinecarboxamide (8a)

White solid: 82% yield; mp 245–247 °C; IR (KBr, cm^−1^) 3300–3200 (3 NH str), 3035 (CH aromatic), 2951, 2924 (CH aliphatic), 1670 (C=O str), 1265, 748; ^1^H NMR (400 MHz, DMSO-d_6_) *δ*: 10.05 (s, 1H, NH, D_2_O exchangeable), 8.46 (s, 2H, 2NH, D_2_O exchangeable), 7.62 (d, *J* = 8.0 Hz, 2H, ArH), 7.51 (d, *J* = 8.0 Hz, 2H, ArH), 7.34–7.29 (m, 3H, ArH), 6.56 (d, *J* = 4.0 Hz, 1H, C4H-pyrrole), 6.14 (d, *J* = 4.0 Hz, 1H, C3H-pyrrole), 3.88 (s, 3H, N-CH_3_), 3.67 (s, 2H, CH_2_C=O), 2.39 (s, 3H, ArCH_3_); ^13^C NMR (100 MHz, DMSO-d_6_) *δ*: 184.8, 168.4, 155.6, 141.9, 137.5, 137.5, 134.7, 133.8, 130.6, 129.3, 129.1, 128.9, 128.8, 122.1, 109.9, 33.4, 32.1, 21.5; Anal. Calcd. for C_22_H_20_Cl_2_N_4_O_3_ (459.33): C, 57.53; H, 4.39; N, 12.20, found C, 57.67; H, 4.50; N, 12.46.

##### N-(2-Chloro-6-methylphenyl)-2–(2-(1-methyl-5–(4-methylbenzoyl)-1H-pyrrol-2-yl)acetyl)hydrazinecarboxamide (8b)

White solid: 87% yield; mp 240–242 °C; IR (KBr, cm^−1^) 3300–3200 (3NH str), 3032 (CH aromatic), 2951, 2924 (CH aliphatic), 1670 (C=O str), 1265, 748; ^1^H NMR (400 MHz, DMSO-d_6_) *δ*: 10.02 (s, 1H, NH, D_2_O exchangeable), 8.30 (s, 1H, NH, D_2_O exchangeable), 8.23 (s, 1H, NH, D_2_O exchangeable), 7.63 (d, *J* = 7.2 Hz, 2H, ArH), 7.36–7.30 (m, 3H, ArH), 7.23–7.15 (m, 2H, ArH), 6.57 (d, *J* = 4.0 Hz, 1H, C4H-pyrrole), 6.14 (d, *J* = 4.0 Hz, 1H, C3H-pyrrole), 3.88 (s, 3H, N-CH_3_), 3.69 (s, 2H, CH_2_C=O), 2.39 (s, 3H, ArCH_3_), 2.21 (s, 3H, ArCH_3_); ^13^C NMR (100 MHz, DMSO-d_6_) *δ*: 184.8, 168.6, 167.4, 156.2, 141.9, 139.4, 137.6, 137.5, 134.5, 132.0, 130.6, 129.3, 129.1, 127.8, 127.2, 122.1, 109.9, 33.4, 32.1, 21.5, 18.8; Anal. Calcd. for C_23_H_23_ClN_4_O_3_ (438.91): C, 62.94; H, 5.28; N, 12.77, found C, 63.20; H, 5.41; N, 12.89.

##### N-(4-Chloro-3-(trifluoromethyl)phenyl)-2–(2-(1-methyl-5–(4-methylbenzoyl)-1H-pyrrol-2-yl)acetyl)hydrazinecarboxamide (8c)

Creamy white solid, 89% yield; mp 171–173 °C; IR (KBr, cm^−1^) 3300–3200 (3NH str), 3032 (CH aromatic), 2900, 2927 (CH aliphatic), 1732, 1662 (C=O str), 1265, 752; ^1^H NMR (400 MHz, DMSO-d_6_) *δ*: 10.01 (s, 1H, NH, D_2_O exchangeable), 9.31 (s, 1H, NH, D_2_O exchangeable), 8.47 (s, 1H, NH, D_2_O exchangeable), 8.06 (d, *J* = 2.4, 1H, ArH), 7.78–7.75 (m, 1H, ArH), 7.64–7.59 (m, 3H, ArH), 7.31 (d, *J* = 8.0 Hz, 2H, ArH), 6.58 (d, *J* = 4.0 Hz, 1H, C4H-pyrrole), 6.16 (d, *J* = 4.0 Hz, 1H, C3H-pyrrole), 3.89 (s, 3H, N-CH_3_), 3.70 (s, 2H, CH_2_C=O), 2.39 (s, 3H, ArCH_3_); ^13^C NMR (100 MHz, DMSO-d_6_) *δ*: 184.8, 168.8, 155.6, 141.9, 139.8, 137.4, 137.3, 132.3, 130.7, 129.4, 127.2, 126.9, 124.6, 123.6, 122.8, 122.1, 121.9, 109.8, 33.4, 32.2, 21.5; Anal. Calcd. for: C_23_H_20_ClF_3_N_4_O_3_ (492.88): C, 56.05; H, 4.09; N, 11.37, found C, 56.23; H, 4.25; N, 11.53.

### Biological activity

#### In vitro antiproliferative activity against NCI 60-cell line

Anticancer activity screening of the newly synthesised compounds was measured *in vitro* utilising 60 different human tumour cell lines provided by US National Cancer Institute according to previously reported standard procedure^41–43^ as follows: Cells are inoculated into 96-well microtiter plates in 100 ml. After cell inoculation, the microtiter plates are incubated at 37 °C, 5% CO_2_, 95% air, and 100% relative humidity for 24 h prior to addition of experimental compounds. After 24 h, two plates of each cell line are fixed *in situ* with TCA, to represent a measurement of the cell population for each cell line at the time of compound addition (*Tz*). Experimental compounds are solubilised in dimethyl sulfoxide at 400-fold the desired final maximum test concentration and stored frozen prior to use. At the time of compound addition, an aliquot of frozen concentrate is thawed and diluted to twice the desired final maximum test concentration with a complete medium containing 50 mg/ml gentamicin. Aliquots of 100 ml of the compounds dilutions are added to the appropriate microtiter wells already containing 100 ml of medium, resulting in the required final compound concentration. Following compound addition, the plates are incubated for an additional 48 h at 37 °C, 5% CO_2_, 95% air, and 100% relative humidity. For adherent cells, the assay is terminated by the addition of cold trichloroacetic acid (TCA). Cells are fixed *in situ* by the gentle addition of 50 ml of cold 50% (w/v) TCA (final concentration, 10% TCA) and incubated for 600 min at 4 °C. The supernatant is discarded, and the plates are washed five times with tap water and air-dried. Sulforhodamine B (SRB) solution (100 ml) at 0.4% (w/v) in 1% acetic acid is added to each well, and plates are incubated for 10 min at room temperature. After staining, the unbound dye is removed by washing five times with 1% acetic acid and the plates are air-dried. The bound stain is subsequently solubilised with a 10 mM trizma base, and the absorbance is read on an automated plate reader at a wavelength of 515 nm. For suspension cells, the methodology is the same except that the assay is terminated by fixing settled cells at the bottom of the wells by gently adding 50 ml of 80% TCA (final concentration, 16% TCA). Using the absorbance measurements [time zero, (*Tz*), control growth, (*C*), and test growth in the presence of compound (*Ti*)], the percentage growth is calculated for each compound. The One-dose data are reported as a mean graph of the percent growth of treated cells. The reported number is growth relative to the no-compound control, and relative to the time zero number of cells. This allows the detection of both growth inhibition and lethality.

#### Measurement of IC_50_ against Colon (HCT-15), renal (UO-31), and leukaemia (HL-60) cancer cell lines

The 3-(4,5-dimethylthiazol-2-yl)-2,5-diphenyltetrazolium bromide (MTT) method^44^ of monitoring *in vitro* cytotoxicity is well suited for use with multiwell plates. The assessment of cell population growth is based on the capability of living cells to reduce the yellow product MTT to a blue product, formazan, by a reduction reaction occurring in the mitochondria. The three cell lines were incubated for 24 h in 96-microwell plates. The number of living cells in the presence or absence (control) of the various test compounds is directly proportional to the intensity of the blue colour, measured by spectrophotometry using (ROBONIK P2000 Spectrophotometer) at a wavelength of 570 nm. Measure the background absorbance of multiwell plates at 690 nm and subtract from the 570 nm measurement. Five concentrations ranging from 0.01 to 100 µM (with a semi-log decrease in concentration) were tested for each of the compounds under study. Each experiment was carried out in triplicate. The IC_50_ values [the concentration required for 50% inhibition of cell viability] were calculated using sigmoidal dose-response curve-fitting models.

#### *In vitro* cell based VEGFR-2 TK inhibitory assay

*In vitro* VEGFR-2 inhibitory activity of compound **5b** was evaluated using ten folds serial dilutions (1.0, 0.1, 0.01, 0.001 µM) against VEGFR-2 (KDR) Kinase Assay Kit Catalog # 40325 according to manufacturer’s instructions. In brief, the master mixture (25 µl per well) was prepared and added to each well. 5 µl of inhibitor solution of each well labelled as “Test Inhibitor” was added. For the “Positive Control” and “Blank,” 5 µl of the same solution without inhibitor (Inhibitor buffer) was added. 3 ml of kinase buffer was prepared by mixing 600 µl of kinase buffer with 2400 µl water. To the wells designated as “Blank,” 20 µl of kinase buffer was added. The amount of VEGFR-2 required for the assay was calculated and the enzyme was diluted to 1 ng/µl with kinase buffer. The reaction was initiated by adding 20 µl of diluted VEGFR-2 enzyme to the wells designated “Positive Control” and “Test Inhibitor Control.” The mixtures were incubated at 30 °C for 45 min. After the 45 min, 50 µl of Kinase-Glo Max reagent was added to each well. The plate was incubated at room temperature for 15 min. Luminescence was measured using the microplate reader.

#### Wound healing assay

Wound healing assay of compound **5b** was evaluated using CytoSelect™ 24-Well Wound Healing Assay kit Catalog # CBA-120 according to manufacturer’s instructions. In brief, the 24-well plate wound healing inserts were allowed to warm up at room temperature for 10 minutes, then to each well, 500 µl of human umbilical vascular endothelial cells (HUVECs) suspension (1.0 × 10^6^) in media containing 10% fetal bovine serum (FBS) was added. Cells were kept in a cell culture incubator until a monolayer form. Carefully the insert was removed from the well. The media were slowly aspirated and discarded from the wells. The wells were washed with media to remove dead cells and debris. Finally, the media were added to wells to keep cells hydrated, then wells were visualized under a light microscope. Media containing the indicated concentrations of compound **5b** (2.4 µM) or sunitinib (3.2 µM) were then added into the wells for 72 h. the wound closure was monitored with a light microscope. The percent closure of the cells into the wound field was measured.

#### Cell cycle analysis

The HCT-15 cells were treated with compound **5b** at its IC_50_ concentration for 24 h. After treatment, the cells were washed twice with ice-cold phosphate buffer saline (PBS), collected by centrifugation, and fixed in ice-cold 70% (v/v) ethanol, washed with PBS, resuspended with 0.1 mg/ml RNase, stained with 40 mg/ml propidium iodide (PI), and analysed by flow cytometry using FACS Calibur (Becton Dickinson)^45^. The cell cycle distributions were calculated using Cell- Quest software (Becton Dickinson). Exposure of HCT-15 cells to compound **5b** resulted in an interference with the normal cell cycle distribution as indicated.

#### Annexin V-FITC apoptosis assay

Apoptosis was determined by staining the cells with Annexin V fluorescein isothiocyanate (FITC) and counterstaining with PI using the Annexin V-FITC/PI apoptosis detection kit (BD Biosciences, San Diego, CA) according to the manufacturer’s instructions. Briefly, 4 × 10^6^ cell/T 75 flask were exposed to compound **5b** at its IC_50_ concentration for 24 h. The cells then were collected by trypsinization and 0.5 × 10^6^ cells were washed twice with PBS and stained with 5 µl Annexin V-FITC and 5 µl PI in 1 × binding buffer for 15 min at room temperature in the dark. Analyses were performed using FACS Calibur flow cytometer (BD Biosciences, San Jose, CA).

### The effect on the apoptotic marker levels

#### Effect of compound 5b on the level of caspase-3 protein

The level of the apoptotic marker caspase-3 was assessed using Human Caspase-3 (active) ELISA Kit Catalog # KHO1091. The procedure of the used kit was done according to the manufacturer’s instructions. Allow all reagents to reach room temperature before use. Gently mix all liquid reagents prior to use. Add 100 µl of the standard diluent buffer to the zero standard wells. Add 100 µl of standards and controls or diluted samples to the appropriate microtiter wells. Incubate for 2 h at room temperature. Pipette 100 µl of Caspase-3 (Active) detection antibody solution into each well. Incubate for 1 h at room temperature. Add 100 µl anti-rabbit IgG HRP working solution to each well. Prepare the working dilution and incubate for 30 min at room temperature. Add 100 µl of stabilised chromogen to each well. The liquid in the wells will begin to turn blue. Incubate for 30 min at room temperature and in the dark. Add 100 µl of stop solution to each well. The solution in the wells should change from blue to yellow. Read the absorbance of each well at 450 nm. Read the plate within 2 h after adding the stop solution. Use a curve fitting software to generate the standard curve. Read the concentrations for unknown samples and controls from the standard curve.

#### Effects of compound 5b on the level of caspase-8 and caspase-9 proteins

To measure caspase-8 and caspase-9 levels, DRG^®^ Caspase-8 (human) (EIA-4863) ELISA and DRG^®^ Caspase-9 (human) (EIA-4860) ELISA kits were used. Briefly, prepare cell extracts after induction of apoptosis. Resuspend the extracts in lysis buffer, incubate 60 min at room temperature, and centrifuge for 15 min. Wash the microwell strips with 400 µl wash buffer. Add 100 µl of sample diluent in duplicate to all standard wells. Add 100 µl of sample diluent in duplicate to the blank wells. Add 50 µl of sample diluent to the sample wells. Add 50 µl of each sample in duplicate to the sample wells. Add 50 µl of detection antibody to all wells and incubate at room temperature for 2 h. Add 100 µl of diluted anti-rabbit-IgG-HRP to all wells, including the blank wells. Pipette 100 µl of (3,3′,5,5′-Tetramethylbenzidine) TMB substrate solution to all wells and incubate at room temperature for about 15 min. Stop the enzyme reaction by pipetting 100 µl of stop solution into each well. Read absorbance of each microwell on a spectro-photometer using 450 nm. Determine the absorbance of both the samples and the standards.

### Molecular modelling studies

#### Molecular docking study

All the molecular modelling studies were performed using Molecular Operating Environment (MOE, 2010.10) software. All minimizations were carried out with MOE until an RMSD gradient of 0.05 kcal mol^−1 ^Å^−1^ with MMFF94x force field and the partial charges were automatically calculated. The X-ray crystallographic structure of VEGFR-2 co-crystallised with sunitinib as an inhibitor (PDB ID: 4AGD)^46^ was downloaded from the protein data bank^47^. The protein structure was prepared for the docking study using Protonate 3D protocol in MOE with default options. The program specifications were adjusted so that the docking site (dummy atoms), the placement methodology (triangle matcher), and the scoring methodology (London dG). Rigid receptor as refinement methodology and GBVI/WSA dG as the scoring methodology for selection of the best poses from 200 different poses for each tested compound. The co-crystalized ligand, sunitinib, was used to define the active site in the protein structures for docking. Docking protocol was first validated by re-docking of the co-crystallised ligand (sunitinib) in the vicinity of the active site of VEGFR-2. The validated docking protocols were then used to investigate the ligand-target interactions in the active site of the target kinase for the newly synthesised compound **5b** to predict its binding pattern and affinity.

#### Physicochemical, ADME, and pharmacokinetic properties prediction

The free SwissADME web tool available from the Swiss Institute of Bioinformatics (SIB) was used for the calculation of the physicochemical descriptors as well as to predict the pharmacokinetic properties, ADME parameters, and drug-like nature of the most potent newly synthesised compound **5b**^48^^,^^49^. The structure of compound **5b** was converted to SMILES notations, then submitted to the online server for calculation.

## Results and discussion

### Chemistry

**Scheme 1.**
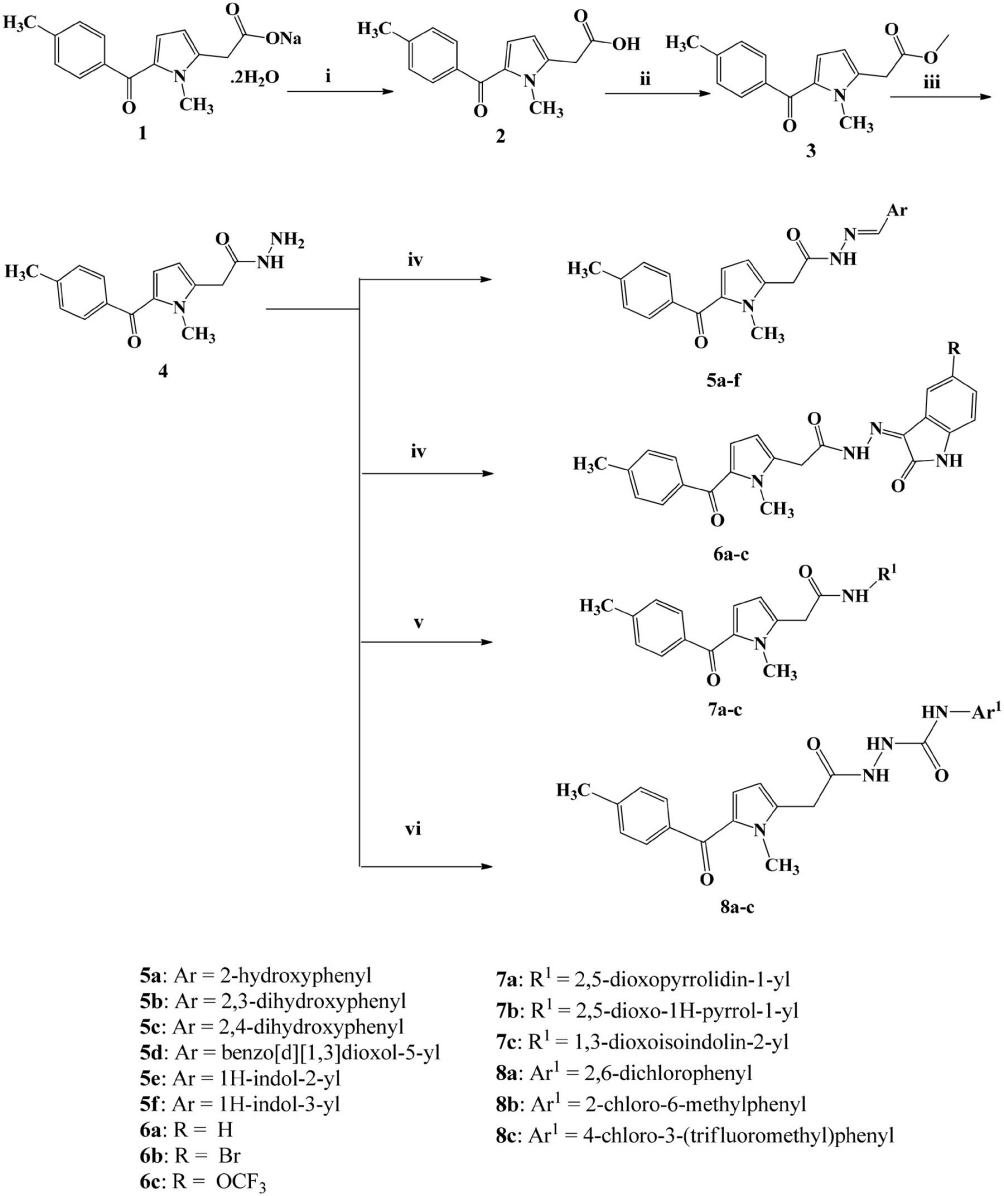
Synthetic pathway of tolmetin hydrazide **4**, tolmetin hydrazones **5a–f**, **6a–c**, tolmetin derivatives**7a–c** and tolmetin semicarbazide derivatives **8a–c**.

Fifteen novel tolmetin derivatives **5a–f**, **6a–c**, **7a–c**, and **8a–c** were synthesised from tolmetin hydrazide **4** according to Scheme **1**. Tolmetin hydrazide **4** was prepared in three steps starting with commercial tolmetin sodium dihydrate **1**. First, 2-(1-methyl-5-(4-methylbenzoyl)-1*H*-pyrrol-2-yl)acetic acid (tolmetin) (**2**) was synthesised through hydrolysis of tolmetin sodium dihydrate **1** under aqueous acidic conditions at room temperature. Methyl 2-(1-methyl-5-(4-methylbenzoyl)-1*H*-pyrrol-2-yl)acetate (**3**) was synthesised from tolmetin **2** by refluxing for 3 h with methanol and few drops of concentrated sulphuric acid. Tolmetin hydrazide **4** was prepared from the ester **3** via reflux for 3 h with 80% hydrazine hydrate in methanol. Hydrazone derivatives **5a–f** were prepared by the reaction of tolmetin hydrazide **4** with different aryl or heteroaryl aldehydes in absolute ethanol with few drops of glacial acetic acid. The IR spectra of **5a–f** showed the amidic C=O stretching as a characteristic strong band in the range 1685–1666 cm^−1^ and the C=N stretching of the azomethine group (N = CH) as a band in the range 1631–1593 cm^−1^.

**Figure 3.**
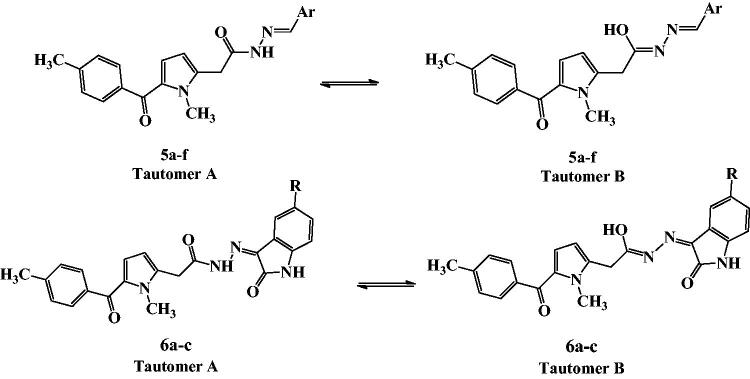
Tautomerism in tolmetin hydrazone derivatives **5a–f** and **6a–c**.

The ^1^H NMR spectra of derivatives **5a–f** displayed the characteristic signals corresponding to different aryl or heteroaryl groups. For all the derivatives **5a–f**, ^1^H NMR spectra showed that the hydrazone exists is a mixture of two tautomers, tautomer **A** (keto amide) and tautomer **B** (enol amide) (Figure 3). In all the ^1^H NMR spectra, CH_2_C=O appeared as two singlet signals with total integration of 2H at the range of *δ* 4.11–4.31 ppm and *δ* 3.73–3.80 ppm corresponding to tautomer **A** and tautomer **B**. Moreover, the azomethine proton of derivatives **5a–f** appeared in the ^1^H NMR spectra as two singlet signals with total integration of 1H, at the range of *δ* 8.14–8.43 ppm and *δ* 7.95–8.34 ppm corresponding to the two tautomers. Additionally, two D_2_O exchangeable peaks with total integration of 1H appeared in the range of *δ* 11.13–11.92 ppm and *δ* 11.04–11.50 ppm corresponding to OH proton in tautomer **B** and NH proton in tautomer **A**, respectively. Another evidence for the presence of two tautomers is the appearance of the NCH_3_ of pyrrole as two singlet signals with total integration of 3H at *δ* 3.90–3.94 ppm and *δ* 3.86–3.92 ppm in all the spectra of tolmetin hydrazones **5a–f**.

Tolmetin derivatives **6a–c** were prepared by the reaction of tolmetin hydrazide **4** with isatin or 5-substituted isatin in absolute ethanol with few drops of glacial acetic acid. The IR spectra of **6a–c** showed the C=O stretching as a characteristic broadband in the range 1697–1693 cm^−1^. ^1^H NMR spectra of these compounds displayed the characteristic signals corresponding to different indoline moieties. The ^1^H NMR spectra of tolmetin derivatives **6a–c** showed also that the hydrazones exist as a mixture of two tautomers, tautomer **A** (keto amide) and tautomer **B** (enol amide) (Figure 3). They showed two D_2_O exchangeable peaks with total integration of 1H in the range of *δ* 12.93–13.00 ppm and *δ* 12.56–12.65 ppm corresponding to OH proton in tautomer **B** and NH proton in tautomer **A**, respectively. The ^1^H NMR of compounds **6a–c**, showed two singlet signals with total integration of 2H at the range of *δ* 4.35–4.38 ppm and the range of *δ* 4.07–4.13 ppm corresponding CH_2_C=O protons. The reaction of tolmetin hydrazide **4** with acid anhydride in presence of anhydrous sodium acetate and acetic acid as solvent, yielded derivatives **7a–c**. IR spectra of **7a–c** showed the presence of 2 C=O stretching as characteristic strong bands in the range 1743–1670 cm^−1^. In all the ^1^H NMR spectra of derivatives **7a–c**, CH_2_C=O protons appeared as a singlet signal at the range *δ* 3.66–3.81 ppm. Also, NCH_3_ appeared as a singlet signal (3H) at the range *δ* 3.85–3.93 ppm. Finally, tolmetin semicarbazide derivatives **8a–c** were prepared by stirring tolmetin hydrazide **4** overnight at room temperature with aryl isocyanate in methylene chloride. IR spectra showed the presence of broadband at 3300–3200 cm^−1^ corresponding to the 3 NH groups. ^1^H NMR spectra of these compounds displayed the characteristic signals corresponding to different NHAr groups. In all **8a–c** ^1^H NMR spectra, C3 and C4 protons of pyrrole appeared as doublet signals at the range *δ* 6.14–6.16 and *δ* 6.56–6.58 ppm, respectively. The coupling constant *J* between C3H and C4H was 4.0 Hz in all tolmetin semicarbazide derivatives **8a–c**.

### Biological activity

#### In vitro antiproliferative activity against NCI 60-cell lines

Fifteen newly synthesised compounds were tested for their *in vitro* antiproliferative activity by the National Cancer Institute (NCI), Maryland, USA^50^, under the Developmental Therapeutic Program (DTP)^41^^,^^43^^,^^51^^,^^52^. The tested compounds were evaluated at a concentration of 10 µM on a panel of 60 tumour cell lines representing leukaemia and non-small cell lung, colon, CNS, melanoma, ovarian, renal, prostate, and breast cancers. The antiproliferative activity was presented by the NCI as percent growth of the treated cells and is presented in Table 1 as growth inhibition percentage (GI%) achieved by the tested compounds. The tested compounds exhibited diverse antiproliferative activities.

**Table 1.** *In vitro* growth inhibition % (GI%) of the synthesised compounds against a panel of tumour cell lines at 10 μM.

Subpanel	Compound ID														
	5a	5b	5c	5d	5e	5f	6a	6b	6c	7a	7b	7c	8a	8b	8c
CCRF-CEM	45.9	86.9	83.2			24.6				^a^	^a^	^a^			^a^
HL-60 (TB)	13	95.9	95.5	^a^	^a^	27.0				^a^	10.6	^a^			
K-562	15.8	69.6	55.1	^a^	^a^	^a^		10.6	^a^	11.2	16.5	^a^	^a^		^a^
MOLT-4	26.0	88.8	87.0		^a^	16.5				^a^	14.0	10.6	^a^	^a^	19.2
RPMI-8226	18.6	64.9	51.6		^a^	15.9		^a^		20.2	^a^		^a^	^a^	11.3
SR	43.8	91.1	82.7	^a^	^a^	11.2		^a^							^a^
A549/ATCC	13.9	41.3	53.4	^a^	^a^	15.4		^a^		11.1	12.8	14	^a^	^a^	21.7
EKVX	17.3	41.2	65.8	^a^	25.7	17.7	^a^	^a^		23.9	25.7	24.5	^a^	^a^	^a^
HOP-62	24	50.1	59.4	^a^	24	^a^	^a^		11.08	15.5	10.4	^a^	11.4	^a^	20
HOP-92	^a^	43.8	16.9		10.3	^a^	11.1	^a^	^a^	19.2			^a^		13.5
NCI-H226	^a^	31	34.3	^a^	15.3	^a^	^a^	^a^	^a^	22.6	22.1	^a^	^a^	^a^	^a^
NCI-H23	17.1	54.6	57.6	^a^	15.4	^a^	^a^	13.7		24.8	23.9	^a^	^a^	^a^	^a^
NCI-H322M	21.1	45.6	62.0			^a^		^a^		^a^	^a^	^a^			14.4
NCI-H460	23.4	91.4	78.9		^a^	^a^				^a^					16.8
NCI-H522	24.9	51.2	44.5	12.4	18.9	18.7	^a^	13.2	11.87	30.8	15.9	14.4	15.9	^a^	21.1
Colo 205		37.0	58.2												^a^
HCC-2998	^a^	29.4	40.4				^a^						^a^		
HCT-116	^a^	69.2	65.1	^a^	^a^	^a^		^a^		11.3	14.0	^a^	^a^		^a^
HCT-15	56.5	84.3	83.2	^a^	12.6	16.8	^a^			^a^	^a^	^a^	^a^	^a^	10.9
HT29		13.3	36.0							^a^					^a^
KM12	19.6	55.8	63.9		^a^			^a^			^a^	^a^			^a^
SW-620	10.4	65.8	57.8	^a^	^a^	^a^	^a^		^a^	^a^	^a^	^a^	^a^	^a^	10.6
SF-268	29.1	62.8	62.7	^a^	^a^	10.5	^a^	^a^	^a^	11.9	12.3	^a^	^a^	^a^	26.3
SF-295	^a^	16.5	52.8	^a^	^a^	^a^	^a^		^a^	^a^	^a^				^a^
SF-539	10.9	66.2	55.6	^a^	^a^	^a^	^a^	^a^	^a^	^a^	^a^		^a^	^a^	^a^
SNB-19	20.7	54	50.6	^a^	17.3	12.5		^a^	^a^	20	11.0	^a^	17.7	16.1	25.8
SNB-75	40.4	74.6	68.9	^a^	15.3	25.5	29.6	36.3	37.12	40.9	16	17.9	28.1	27.9	35.7
U251	23.8	74	62.4	^a^	^a^	10.4	^a^	34.4	^a^	^a^	^a^	^a^	11.7	^a^	^a^
LOX IMVI	42	82.7	81.2	^a^	10.7	16.5	^a^	10.0	^a^	12.8	11.1	10.2	^a^	^a^	10.5
MALME-3M	^a^	41.3	51.4		^a^	19.3		^a^							^a^
M14	17.1	71.5	71.2	^a^		^a^		^a^		^a^	^a^	^a^		^a^	11.7
MDA-MB-435	16.8	38.9	36.9	^a^		^a^	^a^	^a^	^a^	^a^	^a^	^a^			20.7
SK-MEL-2	10.4	^a^	40.6							^a^	^a^	^a^			
SK-MEL-28	15.7	20.0	53.6									^a^			
SK-MEL-5	12.6	65.4	60.7	^a^	^a^	^a^	^a^	^a^	^a^	^a^	^a^	^a^	^a^	^a^	22.3
UACC-257	^a^	10.3	41.7	^a^	^a^		^a^	^a^					^a^		22.9
UACC-62	12.8	60.9	70.2	^a^	15.8	12.2	^a^	^a^	^a^	12.4	^a^		11.1	^a^	19.7
IGROV1	19.1	48.8	58.3	^a^	45.5	15.2	^a^	^a^	^a^	22.3	22.2	14.8	14.0	11.2	36.1
OVCAR-3	19.7	76.5	62.8			^a^									
OVCAR-4	39.8	70.9	79.3	^a^	19.3	11.1		10.1		31.9	^a^		16		
OVCAR-5	^a^	27.4	36.6	^a^	^a^	^a^	^a^	^a^	^a^	10.6	^a^		^a^		10.9
OVCAR-8	21.0	61.4	51.6	^a^	^a^	^a^	^a^	^a^	^a^	11	^a^	^a^	^a^	^a^	10.9
NCI/ADR-RES	30.6	80.9	65.4		^a^	^a^		^a^	^a^	12.8	12.3	^a^			^a^
SK-OV-3	^a^	32.0	54.9		12.1					14	^a^	^a^			
786-0	^a^	23.6	56.6	^a^	^a^	^a^				^a^	^a^				
A498	18	^a^	12.6	13.1	10.9	15.6		10.2		14.5	10.7	13.3	^a^	^a^	18.3
ACHN	46.8	86.5	83.5	^a^	^a^	34.2	^a^	^a^	^a^	11.2	11.1	^a^	^a^	^a^	^a^
CAKI-1	35.5	62.7	53.4	^a^	15.8	17	12.3	15.4	12.64	28.3	20.3	^a^	14.9	11.3	35.2
RXF 393	10.4	70.3	50.3			^a^				^a^	13			^a^	^a^
SN 12 C	10.2	53.2	44.3	^a^	10.5	^a^	^a^	^a^	^a^	19.1	14.8	^a^	^a^	^a^	10.5
TK-10	14.9	61.2	47.1	^a^											
UO-31	59.3	85.8	80.2	22.2	40.4	32.8	25.7	25.0	24.3	41.2	35.3	27.3	31.1	26.1	38.4
PC-3	13.2	40.8	38.9		10.9	^a^	^a^	^a^	^a^	10.0	10.7	^a^	10.5	^a^	22.4
DU-145	10.4	50.7	48.7								^a^				^a^
MCF7	18.5	84	60.8	^a^	22.1	27.0	^a^	^a^	^a^	14.4	13.2	^a^	10.0	^a^	21.7
MDA-MB-231/ATCC	17.4	54.7	48.6	^a^	20.5	12.4	11.4	^a^	^a^	13.7	11.6	^a^	^a^	^a^	16.7
HS 578 T		12.6	25.1	^a^	^a^	^a^					^a^	^a^	^a^	^a^	11.9
BT-5	14.9	54.3	49.2	^a^		^a^				^a^	^a^	^a^	^a^	12.7	11.4
T-47D	24.4	74.1	49.8	15.6	29.4	22.1			^a^	42.8	25.5	25.5	18	^a^	13.8
MDA-MB-468	18.9	45.1	53.2	^a^		16.8	10.5	^a^	^a^	^a^	^a^			^a^	17.5

^a^Growth inhibition % produced by the compound is below 10%.

Compound **5b** (Table 1) exhibited significant antiproliferative activity against 58 cancer cell lines with GI% ranging from 10.3 to 95.9%. It showed potent growth inhibitory effects with GI% from 60.9 to 95.9% against all leukaemia, non-small cell lung (NCI-H460), colon (HCT-116, HCT-15 and SW-620), CNS (SF-268, SF-539, SNB-75, and U251), melanoma (LOX IMVI, M14, SK-MEL-5, and UACC-62), ovarian (OVCAR-3, OVCAR-4, OVCAR-8, and NCI/ADR-RES), renal (ACHN, CAKI-1, RXF 393, TK-10 and UO-31), and breast (MCF7 and T-47D) cell lines.

While compound **5c** exhibited antiproliferative activity against 60 cancer cell lines with GI% ranging from 12.6 to 95.5%. It showed potent growth inhibitory effects with GI% from 60.7 to 95.% against leukaemia (CCRF-CEM, HL-60(TB), MOLT-4 and SR), NSCL (EKVX, NCI-H322M and NCI-H460), colon (HCT-116, HCT-15 and KM12), CNS (SF-268, SNB-75 and U251), melanoma (LOX IMVI, M14, SK-MEL-5, and UACC-62), ovarian (OVCAR-3, OVCAR-4 and NCI/ADR-RES), renal (ACHN and UO-31) and breast (MCF7) cell lines. Regarding sensitivity to individual cell lines, it was noticed that all the newly synthesised compounds exhibited weak to potent antiproliferative activity against renal (UO-31) cell line, while (CAKI-1) cell line demonstrated moderate to strong sensitivity against compounds (**5a–c** and **8c**). The CNS (SNB-75) cell line showed moderate to strong sensitivity against compounds (**5a–c**, **6a,b**, **7a**, and **8c**). Moreover, the NSCLC (NCI-H522) showed moderate to strong sensitivity against compounds (**5b,c** and **7a**).

The relationship between the structure variations of the newly synthesised derivatives and their antiproliferative activity revealed that grafting azomethine spacer between acetamide linker and the terminal hydrophobic moiety had a good impact on the anticancer activity. Tolmetin derivatives incorporating acylhydrazone linker directly connected to a phenyl ring bearing hydroxyl group at ortho position (**5a–c**) or indole ring with a nearby NH group (**5e,f**) showed potent antiproliferative activity. Such a structural motif is well known with its ability to participate in zinc chelation and consequently is essential for procaspase-3 activation and induction of apoptosis. Compounds **5a–c** bearing ortho hydroxyl group showed more potent broad-spectrum anticancer activity than compounds **5e,f** with NH moiety. Further analysis of these compounds clearly revealed that the introduction of the additional hydroxyl group on the phenyl ring in compounds **5b** and **5c** improved the anticancer activity. Compound **5b** with the additional hydroxyl group at position 3 of phenyl ring showed more potent anticancer activity than compound **5c** with the additional hydroxyl group at position 4. An interesting phenomenon is that tolmetin hydrazone derivative **5d** that is devoid of the presence of the aforementioned scaffold was inactive against most investigated cell lines. Tolmetin derivatives **7a–c** having acetamide linker directly attached to heterocyclic ring showed weak to moderate broad-spectrum anticancer activity. It is worth mentioning that, pyrrole ring in derivatives **7a,b** was more tolerated for the anticancer activity than isoindoline moiety in compound **7c**. The incorporation of urea spacer instead of azomethine in tolmetin semicarbazide derivatives **8a–c** caused a marked decrease in the antiproliferative activity. It was noticed that compound **8c** featuring the terminal 4-chloro-3-trifluoromethylphenyl moiety possessed more potent activity than compounds **8a,b**.

#### Measurement of IC_50_ against Colon (HCT-15), renal (UO-31), and leukaemia (HL-60) cancer cell lines

The cytotoxic activity of the most active tolmetin derivatives **5b** and **5c** was examined *in vitro* against three human tumour cell lines namely, leukaemia HL-60 (TB), colon (HCT-15), and renal (UO-31) cell lines using 3‐(4,5‐dimethylthiazol‐2‐yl)‐2,5‐diphenyltetrazolium bromide (MTT) colorimetric assay as described by Mosmann^44^. Sunitinib was included in the experiments as a reference cytotoxic drug. The results were expressed as growth inhibitory concentration (IC_50_) values, which represent the compound concentration required to produce a 50% inhibition of cell growth, calculated from the concentration–inhibition response curve and summarised in Table 2. From the obtained results, it was explicated that, the prepared compounds displayed an excellent growth inhibitory activity against the tested cancer cell lines. Investigations of the cytotoxic activity against UO-31 indicated that it was the most sensitive cell line to the influence of the new derivatives. With respect to the HCT-15 colon carcinoma cell line, compound **5b** displayed the highest anticancer activity with (IC_50_ = 6.62 ± 0.35 µM). In particular, compound **5b** was found to be the most potent derivative overall the tested compounds against HL-60, HCT-15, and UO-31 cancer cell lines with IC_50_ = 10.32 ± 0.55, 6.62 ± 0.35, and 7.69 ± 0.41 µM, respectively, compared to the activity of sunitinib against the three cell lines (IC_50_ = 2.77 ± 0.14, 9.06 ± 0.49, and 18.05 ± 0.95 µM, respectively).

**Table 2.** *In vitro* cytotoxic activities of the selected compounds **5b** and **5c** against HL-60, HCT-15 and UO-31 cell lines and VEGFR-2 kinase assay.

Compound	Cytotoxicity IC_50_ (μM)^a^^,b^			
	HL-60	HCT-15	UO-31	VEGFR-2
**5b**	10.32 ± 0.55	6.62 ± 0.35	7.69 ± 0.41	0.20 ± 0.02
**5c**	31.66 ± 1.70	19.04 ± 1.03	17.43 ± 0.93	NT
Sunitinib	2.77 ± 0.14	9.06 ± 0.49	18.05 ± 0.95	0.11 ± 0.01

^a^IC_50_ values are the mean ± SD of three separate experiments; ^b^The concentration recorded to produce 50% inhibition of cell growth or VEGFR-2.

NT: compound not tested for its VEGFR-2 inhibitory assay.

#### *In vitro* cell-based VEGFR-2 TK inhibitory assay

Based on the docking study results represented in the compound scoring energy and binding mode in the VEGFR-2 active site, the most active antiproliferative derivative **5b** was selected to evaluate its inhibitory activity against VEGFR-2 by using the VEGFR-2 kinase inhibitory kit assay. The results were reported as a 50% inhibition concentration value (IC_50_) calculated from the concentration–inhibition response curve and summarised in Table 2. Sunitinib was used as a positive control in this assay. The tested compound **5b** displayed high inhibitory activity with IC_50_ value in the sub-micromolar range (0.20 µM).

#### Wound healing assay

**Figure 4.**
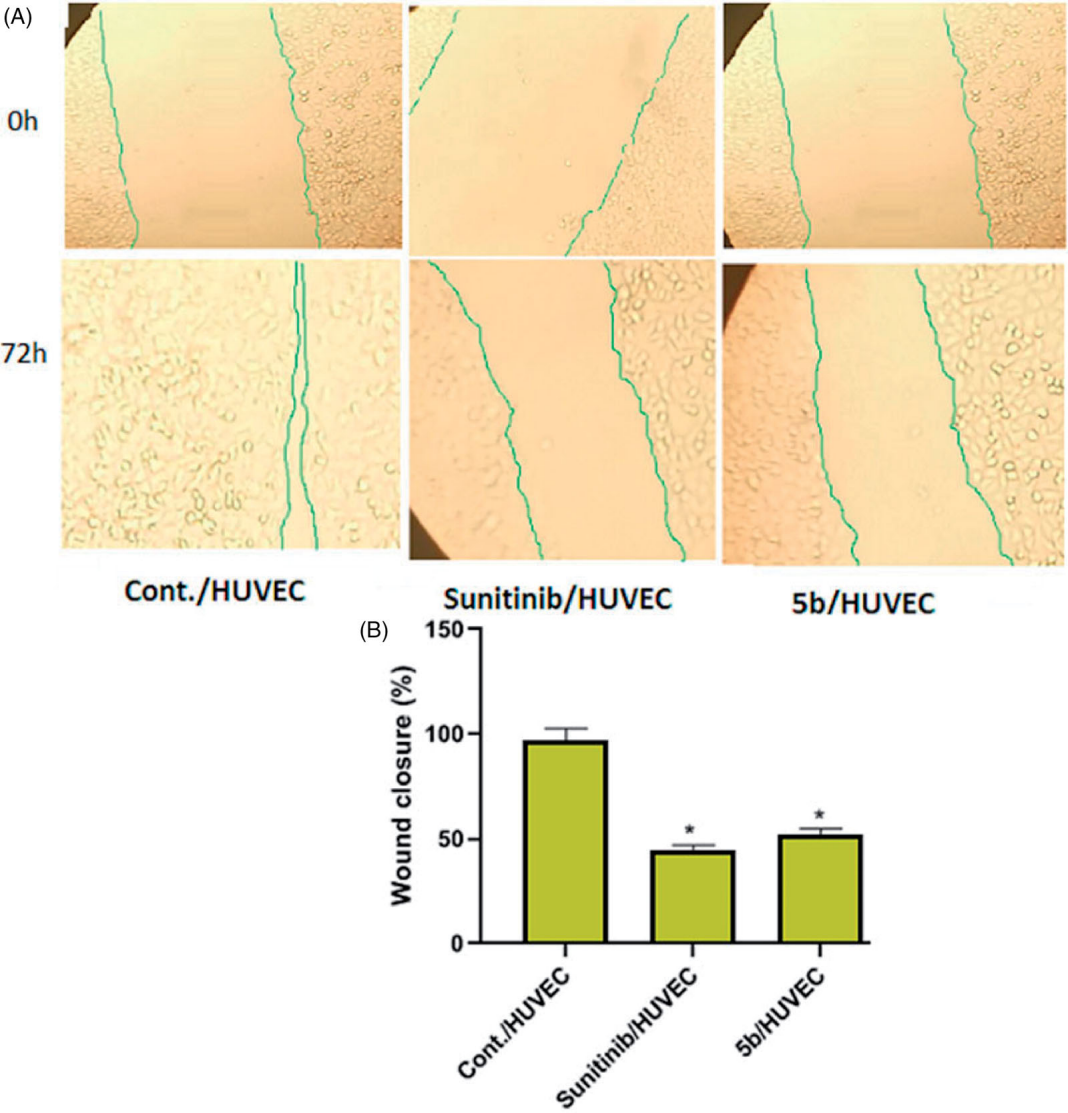
Effects of compound **5b** on endothelial cell migration in HUVEC cells compared to sunitinib. (A) HUVECs were treated with 2.4 μM compound **5b** and 3.2 μM sunitinib for 72 h. (B) Represents the graphical illustration for % of wound closure in control, sunitinib and **5b** treated cells. Data are represented as mean ± SD, *significant from control group at *p*-values <0.001.

One of the hallmarks of angiogenesis is cell migration, which happens in the earlier stages of the angiogenic cascade. A wound-healing assay was performed to investigate *in vitro* anti-angiogenic property of compound **5b**. It reduced HUVECs proliferation and migratory potential. As Figure 4 showed, compound **5b** and sunitinib extremely reduced the HUVECs migration potential exhibiting deeply reduced wound healing patterns after 72 h. Wound closure percentage was significantly lower in the compound **5b** group [51.85 ± 2.92] and sunitinib group [44.44 ± 2.51] when compared to the control group [97.03 ± 5.47] (*p* < 0.001).

#### Cell cycle analysis

**Figure 5.**
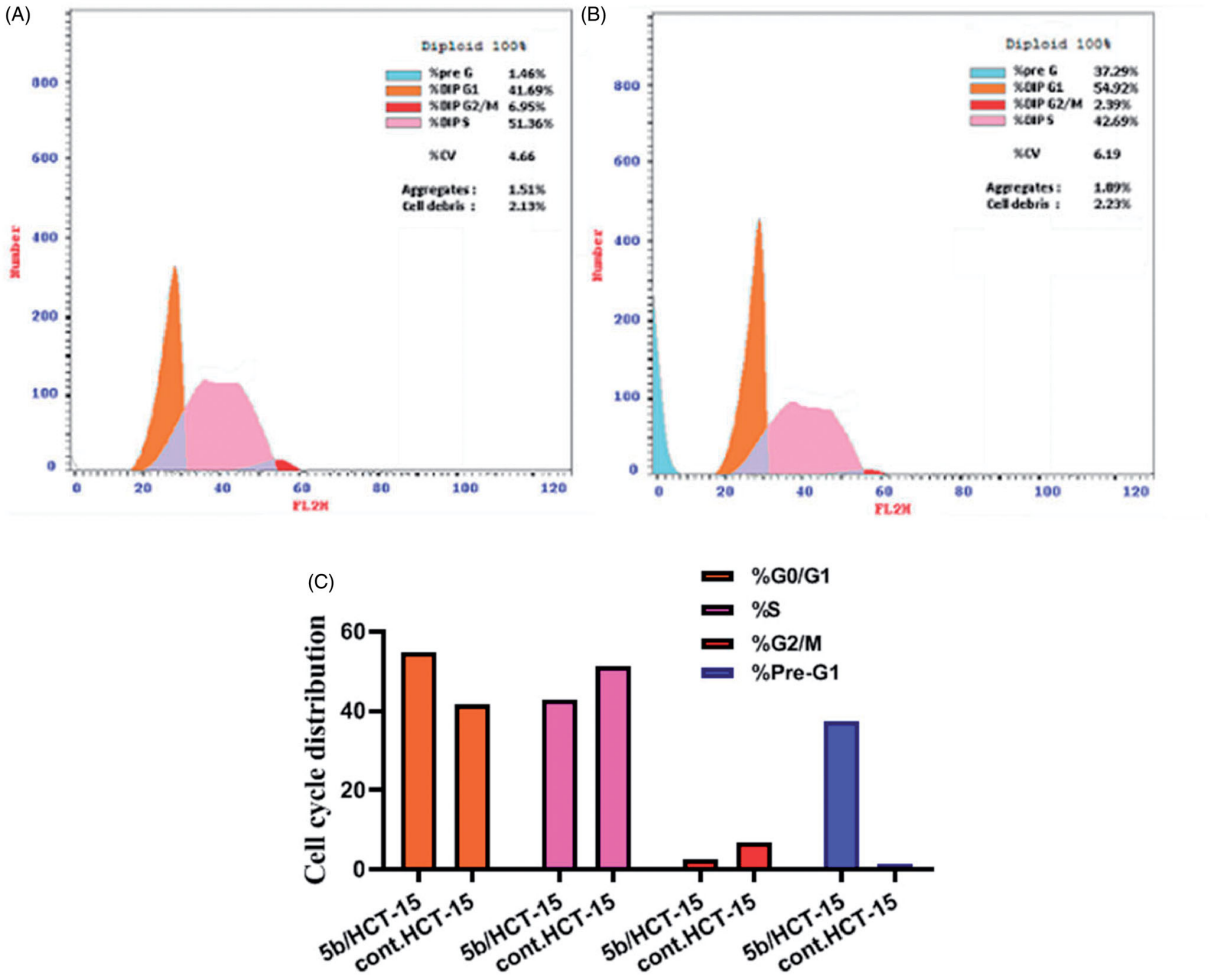
**5b** induces cell cycle arrest in HCT-15 cells. HCT-15 cells were incubated with **5b** or vehicle for 24 h and subjected to cell cycle analysis by flow cytometry. (A) Control and (B) **5b** treated cells. (C) Represents the graphical illustration for cell cycle distribution analysis in control and **5b** treated cells.

Anticancer agents exert their cytotoxic action by aborting cellular proliferation at certain checkpoints. These checkpoints are distinguishable phases in the cell cycle, whose suppression results in the termination of the cell proliferation. Cell cycle analysis employs flow cytometry to distinguish cells within the different cell cycle phases. In this work, the effect of the most potent compound **5b** on the cell cycle progression was scrutinised in order to explore the definite phase at which cell cycle arrest takes place in the HCT-15 colon cancer cell line. HCT-15 cells were treated with IC_50_ concentrations (6.62 µM) of compound **5b** and its effect on the cell population in different cell phases was recorded and displayed in Table 3 and Figure 5. Interestingly, exposure of HCT-15 cells to compound **5b** resulted in significant decline in the cell population at the S and G2/M phases with 16.88 and 65.61%, respectively. Moreover, marked augmentation in the proportion of cells in the G0-G1 phase by 1.32-fold, and in the Sub-G1 phase by 18.7-fold, in comparison to the control (DMSO). This indicates that the target tolmetin derivative **5b** arrested the cell cycle proliferation of HCT-15 cells in the G0-G1 phase.

**Table 3.** Effect of compound **5b** on the cell cycle phases of HCT-15 cells.

Compound	%G0–G1	%S	%G2/M	%Pre-G1
**5b**	54.92	42.69	2.39	37.29
Control	41.69	51.36	6.95	1.46

#### Annexin V-FITC apoptosis assay

Anx. V-based flow cytometry assay represents a helpful tactic for determining whether the death of cells is attributed to programmed apoptosis or to uncontrolled necrosis. Compound **5b** was selected to be tested for its effect on the cell cycle of the HCT-15 cell line because it showed the highest anticancer action towards the aforementioned cells.

**Figure 6.**
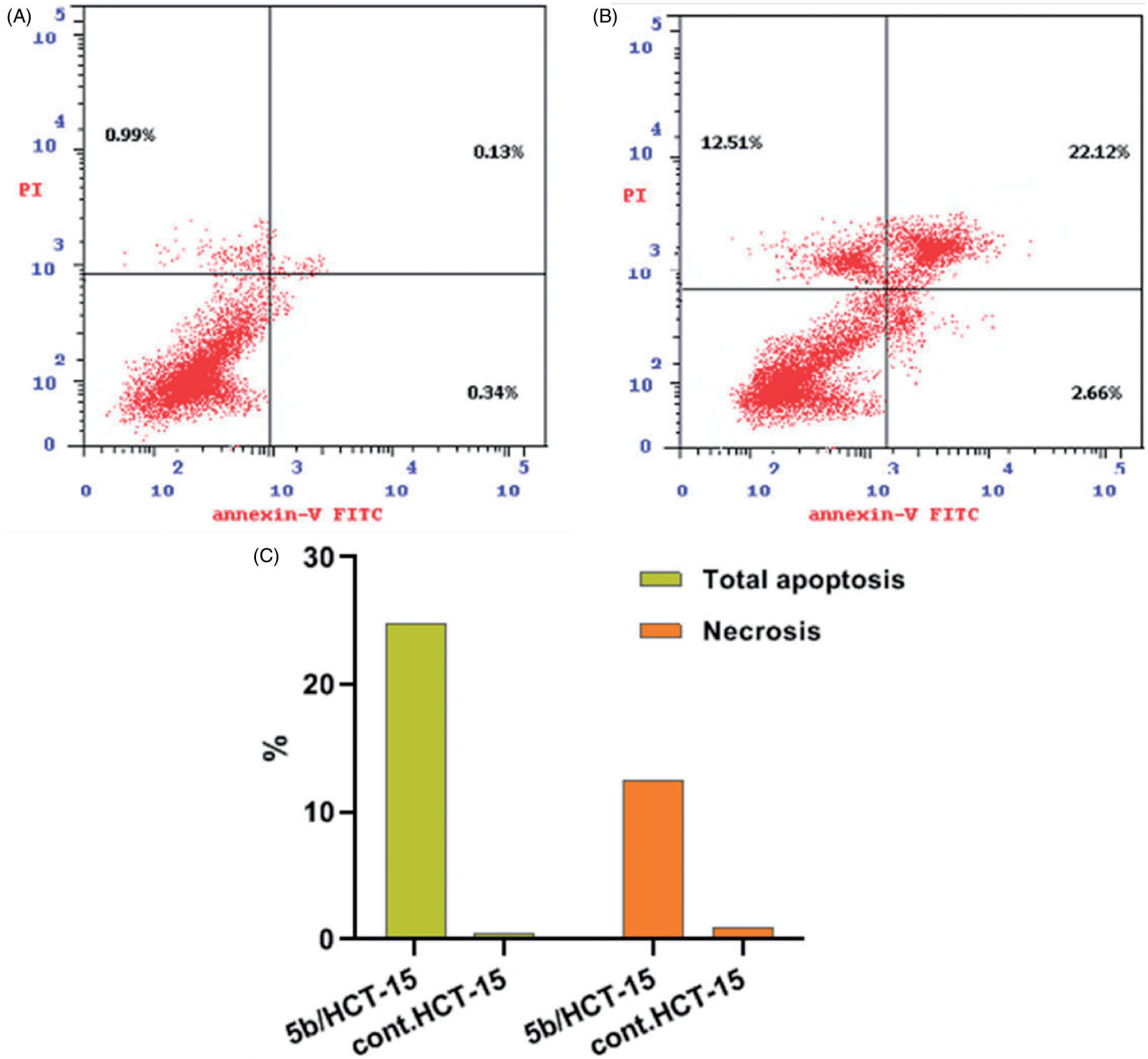
Apoptosis in HCT-15 cells by the treatment with **5b**. (A) Control and (B) **5b** treated cells. (C) Represents the graphical illustration for % of apoptotic and necrotic cells in control and **5b** treated cells. Different cellular distributions are shown in the quadrants, (Q1: Left top) Necrotic cells (Annexin^−,^ PI^+^), (Q2: Right top) Late apoptotic and secondary necrotic cells (Annexin^+^, PI^+^), (Q3: Left bottom) Normal cells (Annexin^−^,PI^−^), and (Q4: Right bottom) Early apoptotic cells (Annexin^+^, PI^−^).

The results revealed a significant elevation in the Anx. V-FITC apoptotic cells percentage in both early (from 0.34 to 2.66%) and late apoptosis (from 0.13 to 22.12%) phases (Table 4 and Figure 6), this corresponds to an increase in the total apoptosis percentage by 52.72-fold, in comparison to the control (DMSO). This clearly verifies that, cell death resulting from the antiproliferative action of the target tolmetin derivative **5b** was attributable to physiological apoptosis.

**Table 4.** Distribution of apoptosis cells in the annexin V-FITC staining assay in HCT-15 cells after treatment with compound **5b**.

Compound	Apoptosis			Necrosis
	Total	Early	Late	
**5b**	24.78	2.66	22.12	12.51
Control	0.47	0.34	0.13	0.99

#### The effect on the apoptotic marker levels

**Figure 7.**
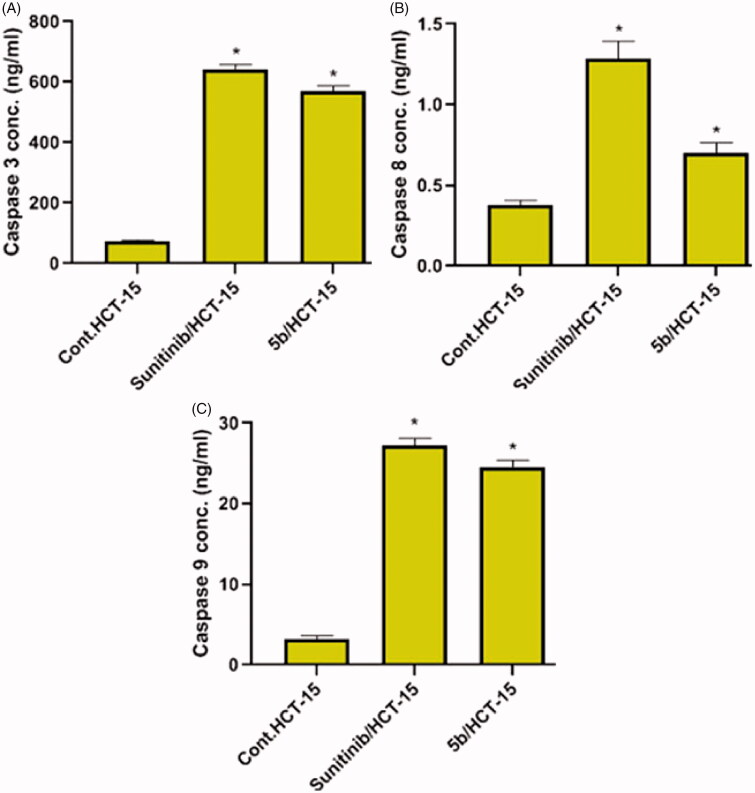
Effects of compound **5b** on the caspase-3, caspase-8 and caspase-9 activity (A, B and C, respectively) in HCT-15 cells compared to sunitinib. Data are represented as mean ± SD, *significant from control group at *p*-values <0.0001.

The activation of caspases as caspase-3, caspase-8, and caspase-9 is responsible for apoptosis, particularly caspase-3 which is an effector caspase that has an important role in apoptosis. It is activated by one of the initiator caspases which results in the activation of certain enzymes responsible for the fragmentation of DNA^53^. Caspase-8 and -9 are initiator caspases that are responsible for caspase-3 activation^54^. Therefore, the apoptosis induction by the compound **5b**, in HCT-15 cells was investigated via caspase-3, caspase-8, and caspase-9 assays, compared to sunitinib as a reference drug. Auspiciously, compound **5b** evidently enhanced the level of the apoptotic caspases-3, -8 and -9 by 7.808-, 1.867- and 7.622-fold, respectively. Compared to sunitinib which markedly boosted the levels of caspases by 8.780-, 3.406- and 8.453-fold, respectively (Table 5 and Figure 7). This suggested that compounds **5b** might induce apoptosis through a caspase-dependent pathway.

**Table 5.** Effect of compound **5b** on the expression of caspase-3, -8 and -9 in HCT-15 cancer cells.

Compound	Caspase-3 (ng/ml^a^)	Caspase-8 (ng/ml^a^)	Caspase-9 (ng/ml^a^)
**5b**	0.570 ± 0.01	0.704 ± 0.06	24.58 ± 0.82
Sunitinib	0.641 ± 0.02	1.284 ± 0.11	27.26 ± 0.82
Control	0.073 ± 0.01	0.377 ± 0.03	3.225 ± 0.44

^a^The values given are means ± SD of three experiments.

### Molecular modelling studies

#### Molecular docking study

**Figure 8.**
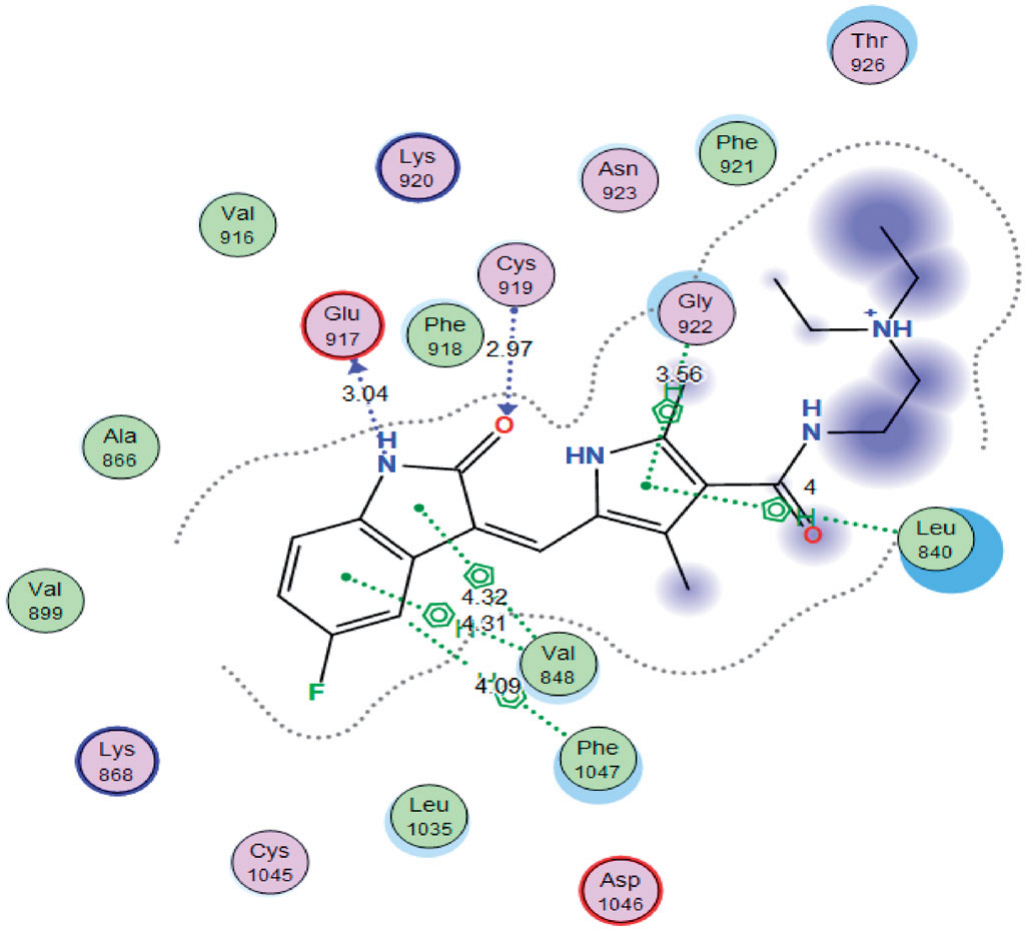
2D interaction diagram showing sunitinib docking pose interactions with the key amino acids in the VEGFR-2 binding site.

**Figure 9.**
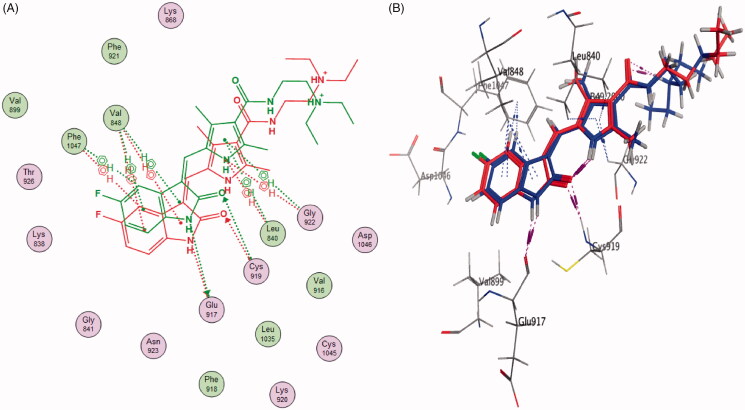
2D diagram (A) and 3D representation (B) of the superimposition of the co-crystallised (red) and the docking pose (blue) of sunitinib in the VEGFR-2 binding site with RMSD of 1.48 Å.

In order to study the binding characteristics of the newly synthesised compounds in the binding site of VEGFR-2, molecular docking studies were performed by Molecular Operating Environment (MOE, 2010.10) software. The X-ray crystallographic structure of VEGFR-2 (PDB ID: 4AGD)^46^ in their active (DFG-in) conformation was downloaded from the Protein Data Bank (PDB)^47^. The downloaded protein is co-crystalized with a type I protein kinase inhibitor, sunitinib. Molecular docking protocol was initially validated by re-docking of the co-crystalized ligand, sunitinib, in the binding site of VEGFR-2. The simulation successfully reproduced the binding pattern of the co-crystalized ligand in the VEGFR-2 binding site with an energy score of −8.4167 kcal/mol, and with an RMSD of 1.48 Å, between the docked pose and the co-crystalized ligand. In addition, the docking pose reproduced all the key interactions achieved by the co-crystallised ligand with the binding site hot spots in VEGFR-2 (Glu917, Cys919) (Figures 8 and 9). The validation step results demonstrate the appropriateness of the utilised molecular docking protocol for the molecular docking study of the newly synthesised compounds in the binding site of VEGFR-2.

**Figure 10.**
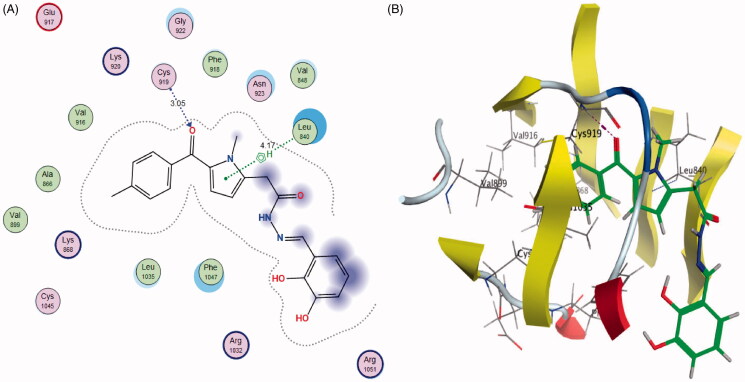
2D diagram (A) and 3D representation (B) of compound **5b** in the VEGFR-2 binding site.

Generally, the newly synthesised compound **5b** showed a comparable binding pattern in the binding site of VEGFR-2 with a predicted docking energy score of −7.3725 kcal/mol, in comparison to the native ligand binding score of −8.4167 (Figure 10).

The designed compound **5b** showed a general promising binding pattern as it interacted with the key amino acids in the VEGFR-2 binding site (Table 6). The pyrrole ring was accommodated in the linker region between the ATP binding domain and the DFG domain of the enzyme and form pi–H bond with leu840. The carbonyl group of *p*-tolylmethanone moiety bind to the ATP binding pocket in the active “DFG-in” conformation via hydrogen bonding with the hinge region amino acid Cys919 in VEGFR-2, and extends to fit the hydrophobic domain (Phe1047, Leu1035, and Lys868, Val899, Cys1045, Ala866, Lys920, and Val916) in and around the adenine region of VEGFR-2 (Figure 10). On the other side, this orientation of the hydrophobic substituents on the hydrazone group extends and fits the allosteric hydrophobic pocket.

**Table 6.** Docking energy scores (S) in kcal/mol, interacting amino acid, Distance in Å, H-bonding energies in kcal/mol of the tested compound (**5b**), sunitinib and their inhibitory activity (IC_50_ μM).

Compound	S Score^a^ (Kcal/mol)	Amino acid/bond	Distance (Å)	H-bond energies (kcal/mol)	RMSD_Refine^b^	VEGFR-2 IC_50_ (μM)^c^
**5b**	−7.3725	Cys919/H-acceptor	3.05	−2.4	1.75	0.20 ± 0.02
		Leu840/pi-H	4.17	−0.5		
Sunitinib	−8.4167	Cys919/H-acceptor	2.97	−0.6	1.48	0.11 ± 0.01
		Glu917/H-donor	3.04	−3.6		
		Gly922/pi-H	3.56	−0.6		
		Leu840/pi-H	4.00	−0.6		
		Phe1047/pi-H	4.09	−1		
		Val848/pi-H	4.32	−0.5		
		Val848/pi-H	4.31	−0.7		

^a^S: The score of placements of a compound into binding pocket of protein using London dG scoring function; ^b^RMSD_Refine: the root-mean-squared-deviation (RMSD) between the heavy atoms of the predicted pose (after refinement) and those of the crystal structure (before refinement); ^c^IC_50_ values are the mean ± SD of three separate experiments.

#### Physicochemical, ADME, and pharmacokinetic properties prediction

**Figure 11.**
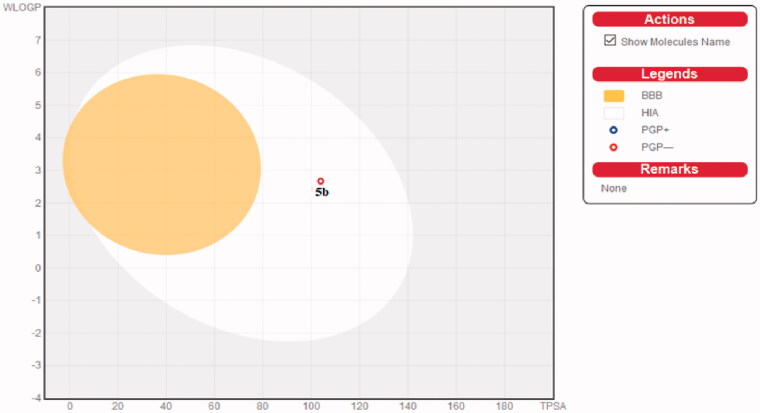
Predicted Boiled-Egg plot from SwissADME online web tool for compound **5b**.

SwissADME online web tool provided by the Swiss Institute of Bioinformatics (SIB) is utilised for the computation of the physicochemical properties and the expectation of the pharmacokinetic properties and drug-like nature of the most active tolmetin derivative **5b**^48^. This was performed to assure that compound **5b** is a promising candidate in terms of biological efficacy and pharmacokinetic characteristics. The submitted compound **5b** exhibited an anticipated wlogP value of 2.76, moderate water solubility, high GIT absorption (high oral bioavailability) with no BBB permeability and so no predicted CNS adverse effects. Figure 11 illustrates the BOILED-Egg graph of the WLOGP vs. TPSA (Topological Polar Surface Area) for the submitted compound **5b**^49^. Compound **5b** was placed in the area of human intestinal absorption (HIA) with no BBB permeability. Moreover, this graph shows that compound **5b** was not P-glycoprotein substrate (PGP-), so it is not amenable to the efflux system done by this transporter which is utilised by numerous tumour cells lines as a drug-resistance mechanism.

SwissADME online web tool also showed that compound **5b** (due to its high molecular weight and logP) satisfies the drug-likeness characteristics as defined by the major pharmaceutical companies; Lipinski’s (Pfizer)^55^, Ghose’s (Amgen)^56^, Veber’s (GSK)^57^ filters. Concisely, the computational study of the physicochemical and pharmacokinetic properties confirmed that compound **5b** exhibited promising biological efficiency with hopeful pharmacokinetic properties.

## Conclusion

A series of tolmetin analogs **5a–f, 6a–c, 7a–c**, and **8a–c** were synthesised. All the synthesised compounds were chosen by the USA National Cancer Institute (NCI) for testing their antiproliferative action on a panel of 60 cell lines. Compounds **5a–c** exhibited broad antiproliferative activity on numerous cell lines. SAR studies revealed that grafting azomethine spacer between acetamide linker and the terminal hydrophobic moiety had a good impact on the anticancer activity. Tolmetin derivatives incorporating azomethine linker directly connected to a phenyl ring bearing hydroxyl group at ortho position (**5a–c**) or indole ring with a nearby NH group (**5e,f**) showed potent anti-proliferative activity. Compound **5b** was found to be the most potent derivative against HL-60, HCT-15, and UO-31 cancer cell lines with IC_50_ values of 10.32 ± 0.55, 6.62 ± 0.35, and 7.69 ± 0.41 µM, respectively. The results of the molecular docking study confirmed that the binding mode of compound **5b** was consistent with its VEGFR-2 inhibitory activity, exerting IC_50_ value in the sub-micromolar range (0.20 µM). Compound **5b** extremely reduced the HUVECs migration potential exhibiting deeply reduced wound healing patterns after 72 h. Wound closure percentage was significantly lower in the compound **5b** group [51.85 ± 2.92] when compared to the control group [97.03 ± 5.47] (*p* < 0.001). The most potent congeners **5b** induced apoptosis in HCT-15 cells (52.72-fold increase in the total apoptosis percentage) as evident by Annexin V-FITC/PI assay. This evidence was supported by an increase in the level of apoptotic caspases-3, -8 and -9 by 7.808-, 1.867- and 7.622-fold, respectively. Moreover, scrutinising results of the cell cycle analysis unravelled that compound **5b** arrested the cell cycle mainly in the G0/G1 phase. Compound **5b** is not only with significant anticancer activity, but also possessed promising pharmacokinetic properties.

## Supplementary Material

Supplemental Material
