# Supplementary material for: Design, synthesis, anticancer evaluation, and molecular modelling studies of novel tolmetin derivatives as potential VEGFR-2 inhibitors and apoptosis inducers
Source: J Enzyme Inhib Med Chem. 2021 Apr 26;36(1):922–39. doi: 10.1080/14756366.2021.1901089 (PMC8079033; doi:10.1080/14756366.2021.1901089)
Supplement: Supplemental Material [file IENZ_A_1901089_SM5324.pdf]

Supporting information

Design, synthesis, anticancer evaluation and molecular modeling studies of novel tolmetin derivatives as potential VEGFR-2 inhibitors and apoptosis inducers

Asmaa E. Kassab [a], Ehab M. Gedawy [a,b], Mohammed I. A. Hamed [c], Ahmed S. Doghish [d,e], Rasha A. Hassan [a]

[a] Department of Pharmaceutical Organic Chemistry, Faculty of Pharmacy, Cairo University, Cairo, 11562, Egypt.

[b] Department of Pharmaceutical Chemistry, Faculty of Pharmacy and Pharmaceutical Industries, Badr University in Cairo (BUC), Badr City, Cairo, 11829, Egypt.

[c] Department of Organic and Medicinal Chemistry, Faculty of Pharmacy, Fayoum University, Fayoum, 63514, Egypt.

[d] Department of Biochemistry, Faculty of Pharmacy (Boys), Al-Azhar University, Nasr City, Cairo, 11651, Egypt.

[e] Department of Biochemistry, Faculty of Pharmacy and Pharmaceutical Industries, Badr University in Cairo (BUC), Badr City, Cairo, 11829, Egypt.

Corresponding author: Email address: asmaa.kassab@pharma.cu.edu.eg

Compound 5a

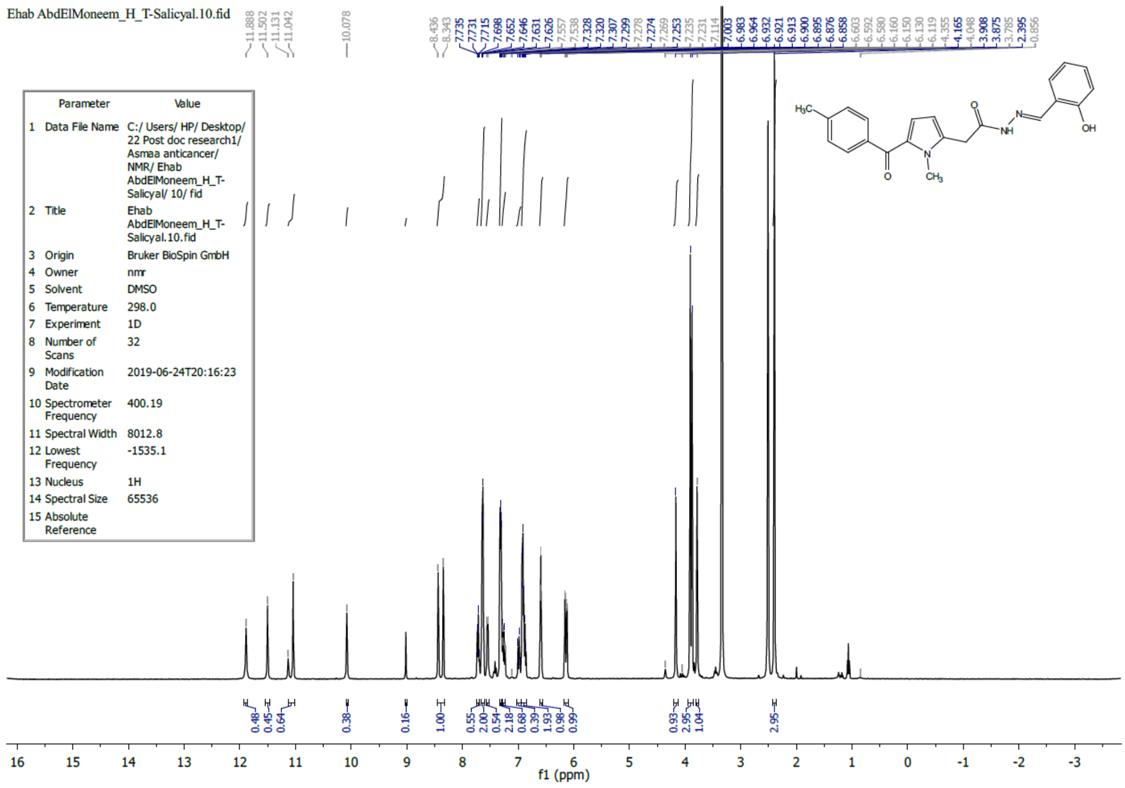
Ehab AbdElMoneem_H_T-Salicyal.10.fid
Parameter Value
1 Data File Name C:/ Users/ HP/ Desktop/ 22 Post doc research1/ Asmaa anticancer/ NMR/ Ehab AbdElMoneem_H_T-Salicyal/ 10/ fid
2 Title Ehab AbdElMoneem_H_T-Salicyal.10.fid
3 Origin Bruker BioSpin GmbH
4 Owner nmr
5 Solvent DMSO
6 Temperature 298.0
7 Experiment 1D
8 Number of Scans 32
9 Modification Date 2019-06-24T20:16:23
10 Spectrometer Frequency 400.19
11 Spectral Width 8012.8
12 Lowest Frequency -1535.1
13 Nucleus 1H
14 Spectral Size 65536
15 Absolute Reference
f1 (ppm)

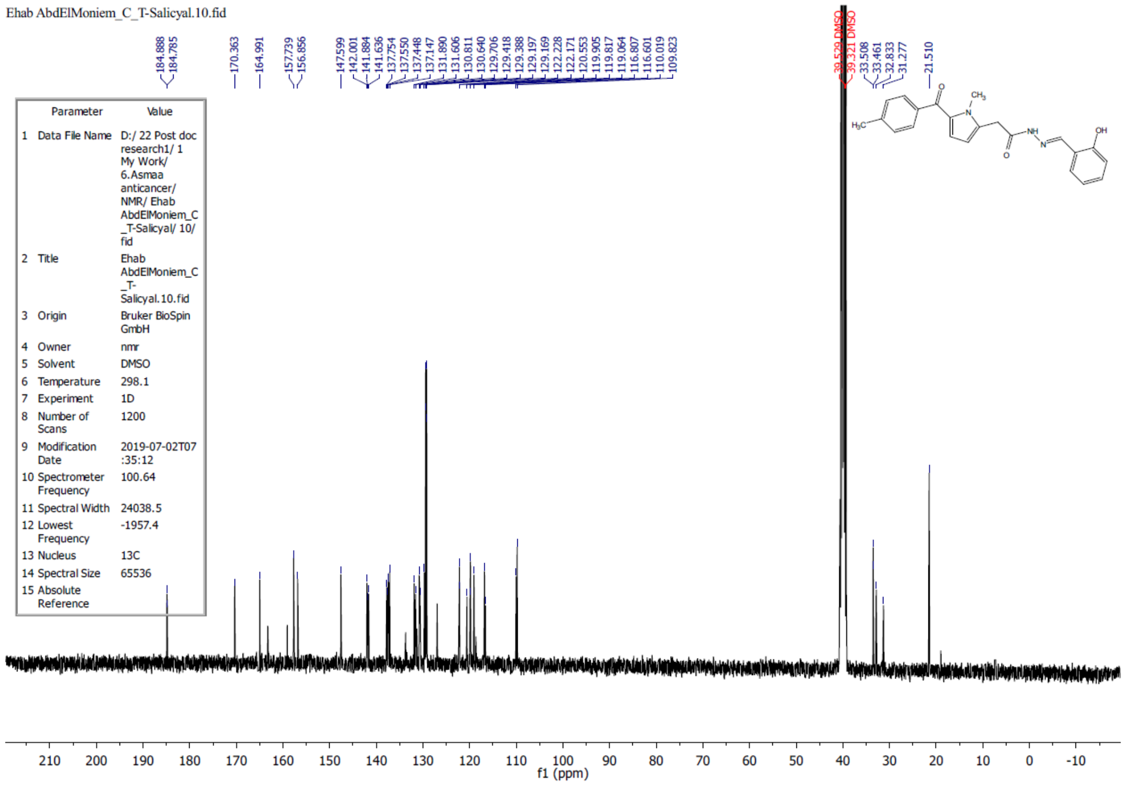
Ehab AbdElMoniem_C_T-Salicyal.10.fid
Parameter Value
1 Data File Name D:/ 22 Post doc research1/ 1 My Work/ 6.Asmaa anticancer/ NMR/ Ehab AbdElMoniem_C_T-Salicyal/ 10/ fid
2 Title Ehab AbdElMoniem_C_T-Salicyal.10.fid
3 Origin Bruker BioSpin GmbH
4 Owner nmr
5 Solvent DMSO
6 Temperature 298.1
7 Experiment 1D
8 Number of Scans 1200
9 Modification Date 2019-07-02T07:35:12
10 Spectrometer Frequency 100.64
11 Spectral Width 24038.5
12 Lowest Frequency -1957.4
13 Nucleus 13C
14 Spectral Size 65536
15 Absolute Reference
f1 (ppm)

Compound 5b

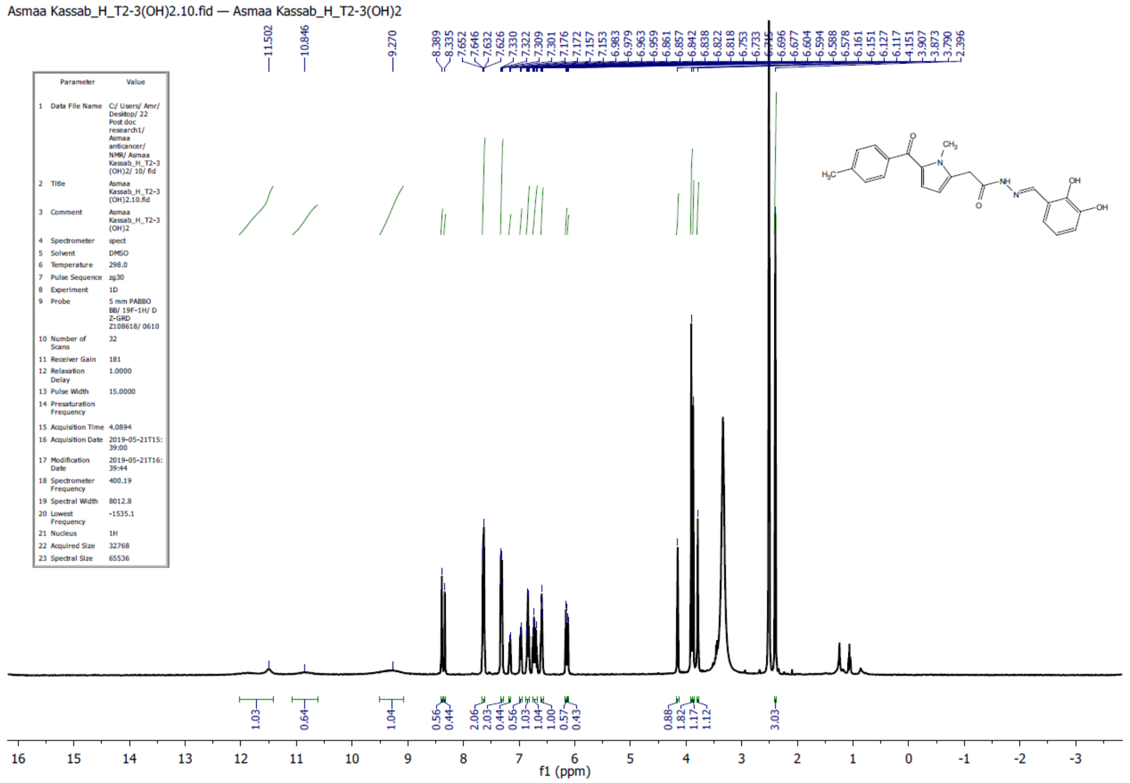

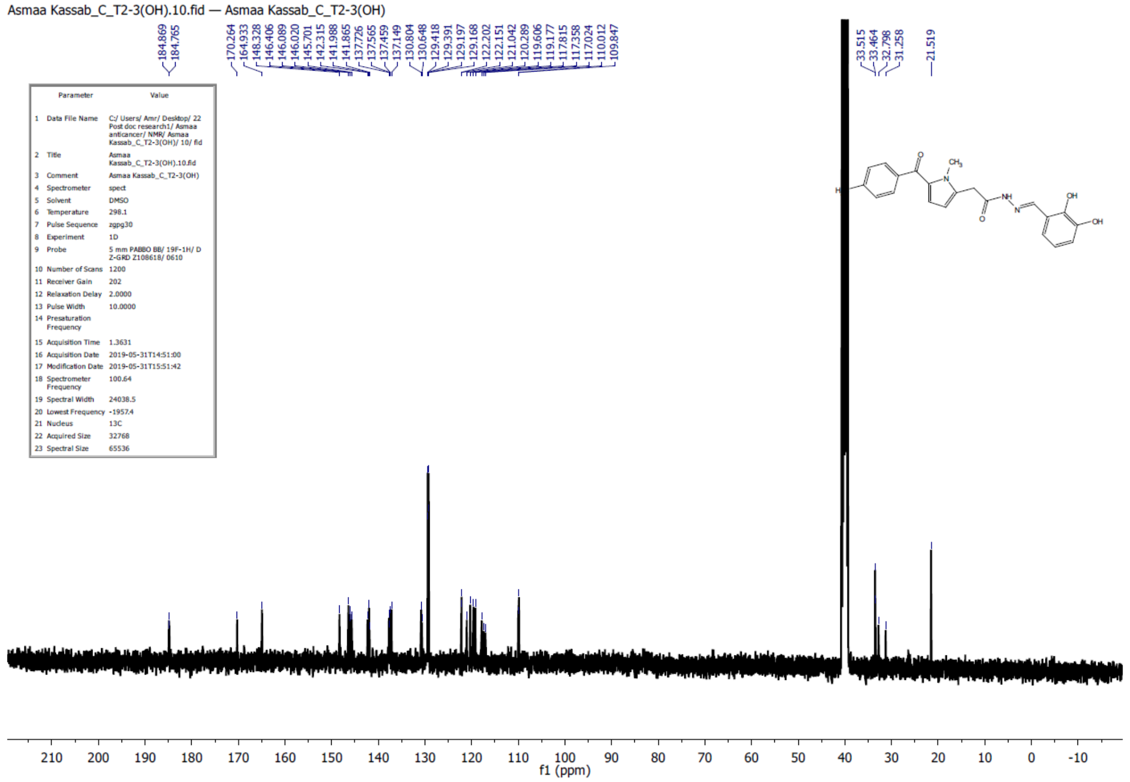

Compound 5c

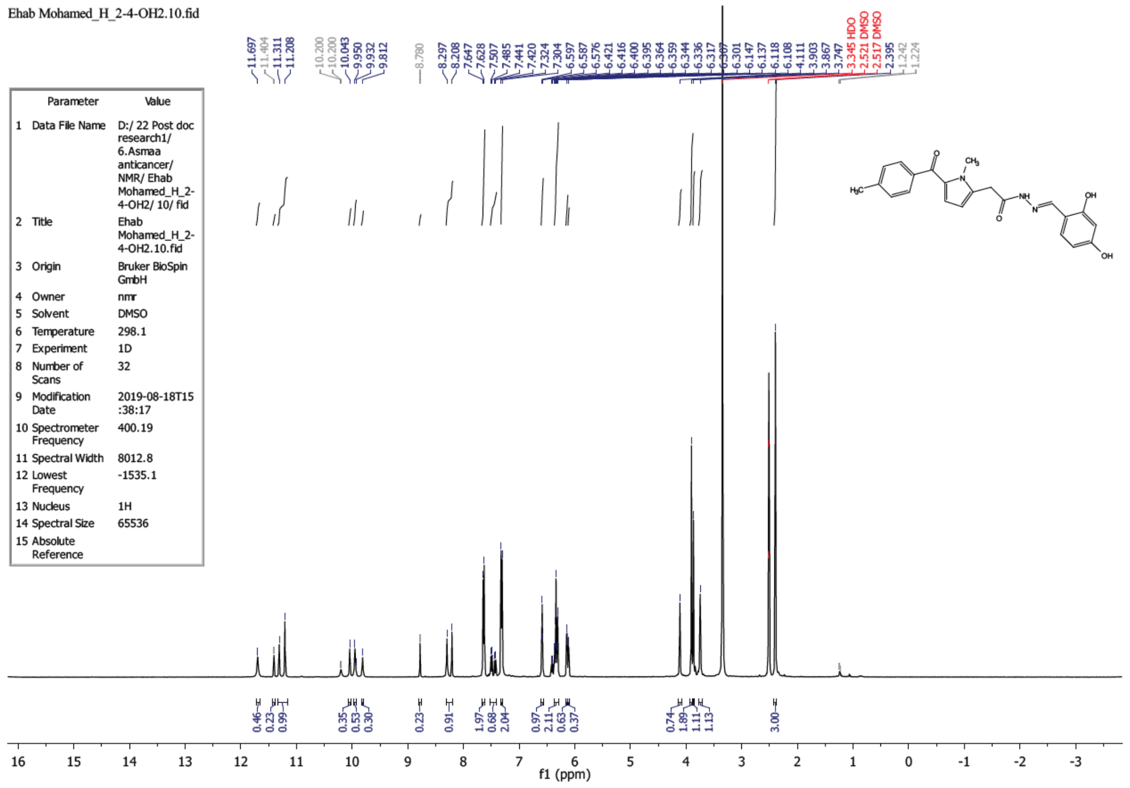

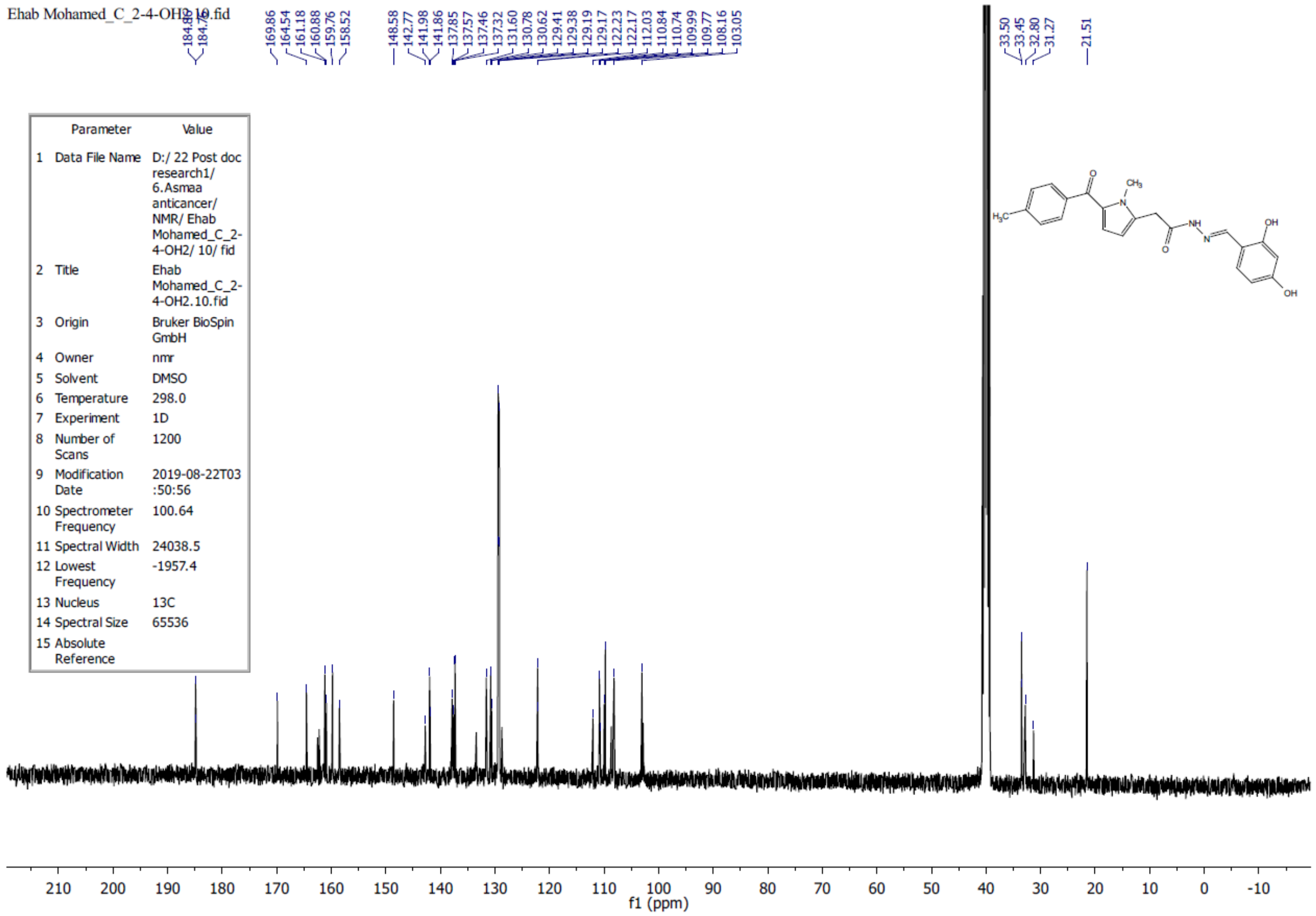

Compound 5d

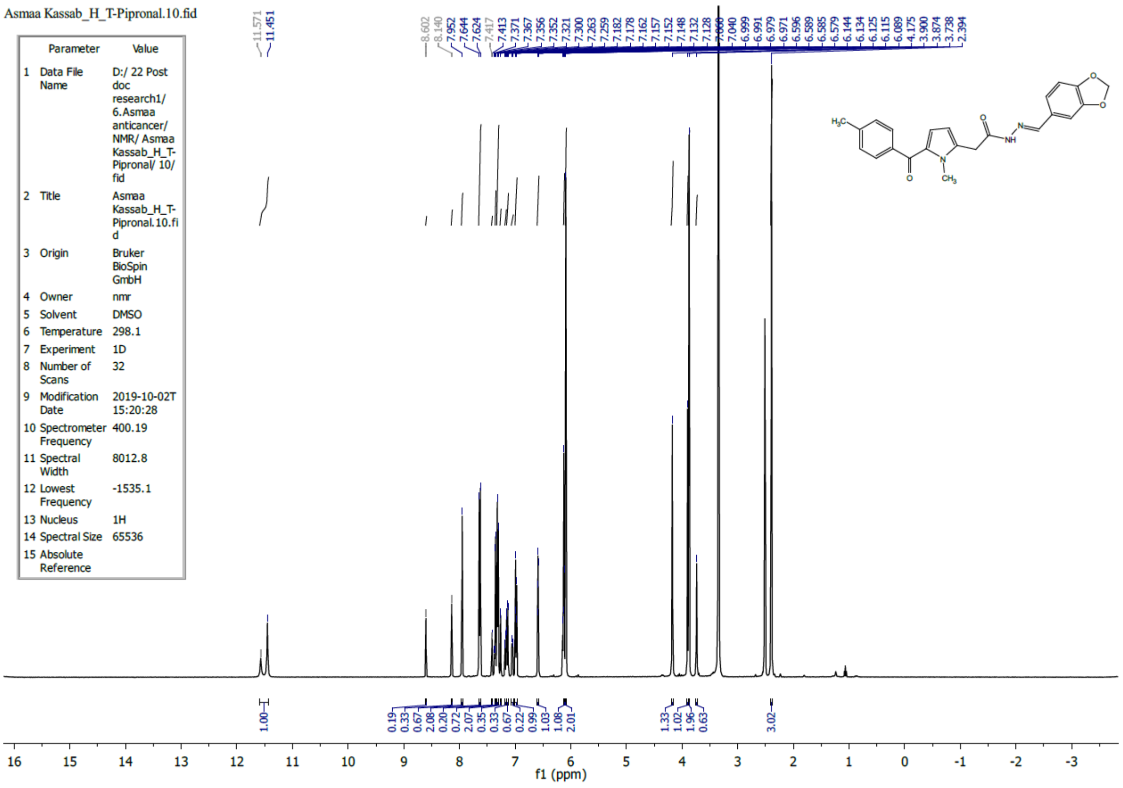

Asmaa Kassab_H_T-Pipronal.10.fid
Parameter Value
1 Data File Name D:/ 22 Post doc research1/ 6.Asmaa anticancer/ NMR/ Asmaa Kassab_H_T-Pipronal/ 10/ fid
2 Title Asmaa Kassab_H_T-Pipronal.10.fid
3 Origin Bruker BioSpin GmbH
4 Owner nmr
5 Solvent DMSO
6 Temperature 298.1
7 Experiment 1D
8 Number of Scans 32
9 Modification Date 2019-10-02T 15:20:28
10 Spectrometer Frequency 400.19
11 Spectral Width 8012.8
12 Lowest Frequency -1535.1
13 Nucleus 1H
14 Spectral Size 65536
15 Absolute Reference
f1 (ppm)

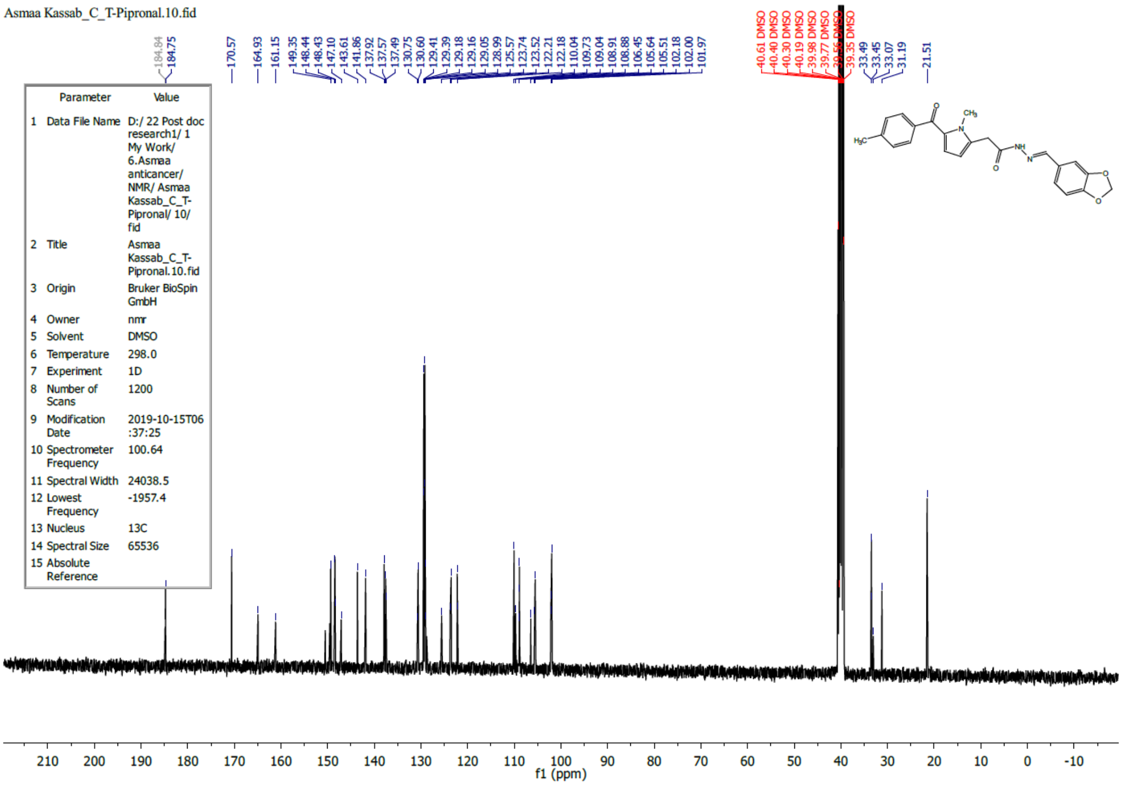

Asmaa Kassab_C_T-Pipronal.10.fid
Parameter Value
1 Data File Name D:/ 22 Post doc research1/ 1 My Work/ 6.Asmaa anticancer/ NMR/ Asmaa Kassab_C_T-Pipronal/ 10/ fid
2 Title Asmaa Kassab_C_T-Pipronal.10.fid
3 Origin Bruker BioSpin GmbH
4 Owner nmr
5 Solvent DMSO
6 Temperature 298.0
7 Experiment 1D
8 Number of Scans 1200
9 Modification Date 2019-10-15T06:37:25
10 Spectrometer Frequency 100.64
11 Spectral Width 24038.5
12 Lowest Frequency -1957.4
13 Nucleus 13C
14 Spectral Size 65536
15 Absolute Reference
f1 (ppm)

Compound 5e

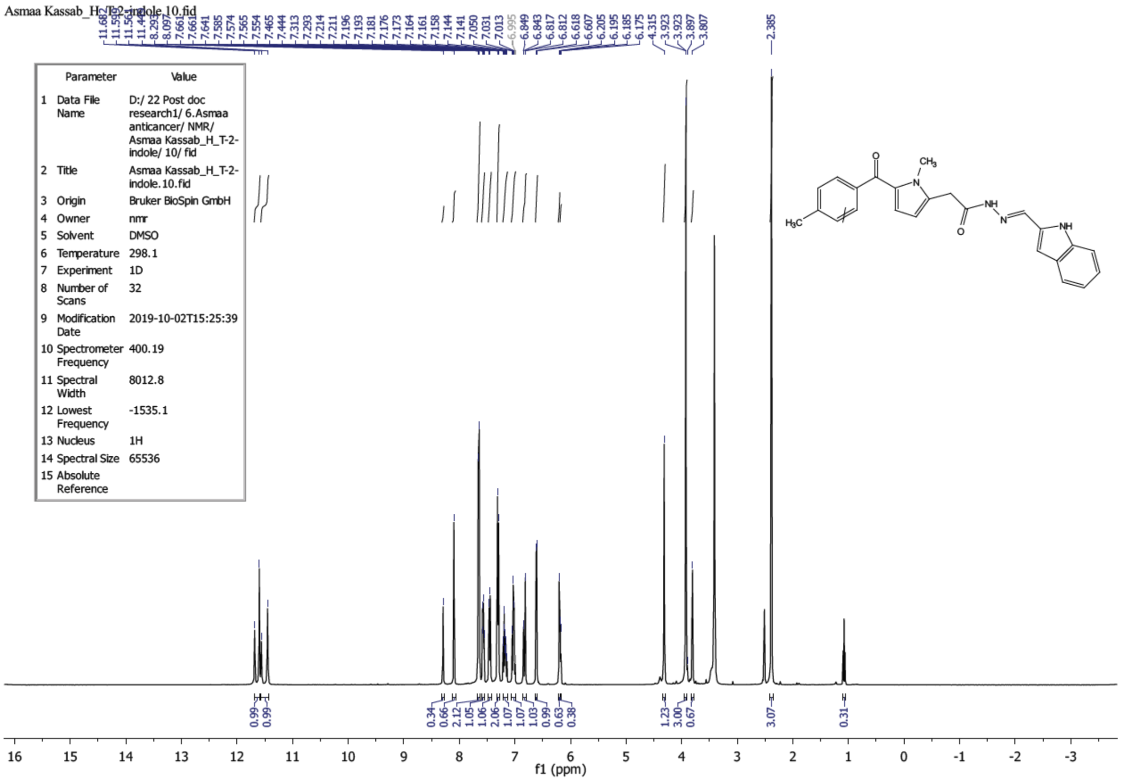

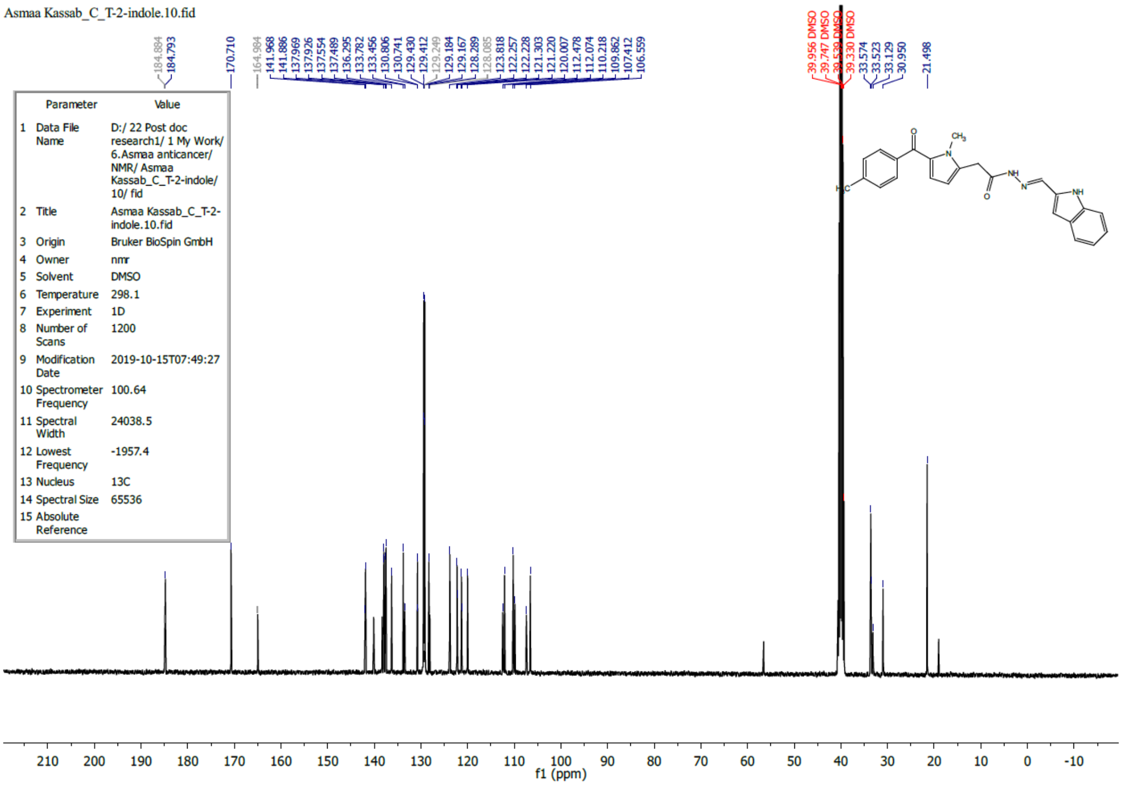

Compound 5f

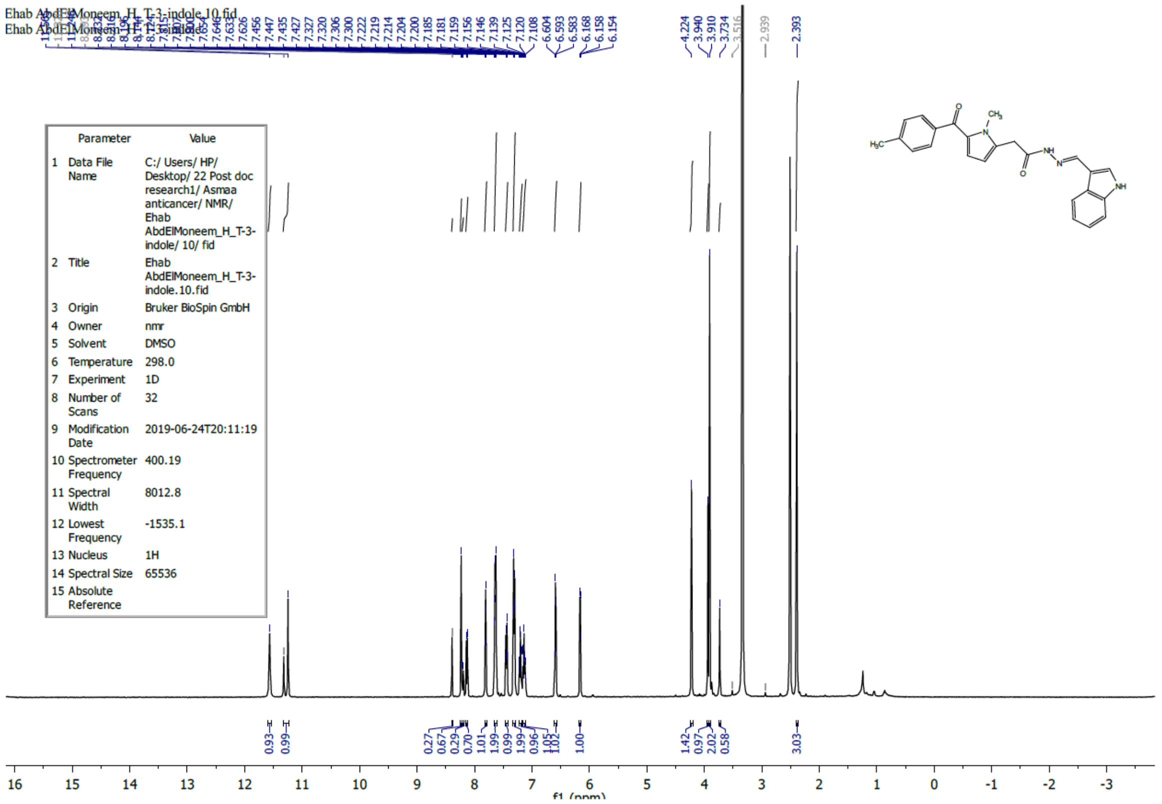

Ehab AbdElMoneem_H_T-3-indole.10.fid
7.456
7.447
7.435
7.427
7.327
7.320
7.306
7.300
7.222
7.219
7.214
7.204
7.200
7.185
7.181
7.159
7.156
7.146
7.139
7.125
7.120
7.108
6.604
6.593
6.583
6.168
6.158
6.154
4.224
3.940
3.910
3.734
3.516
2.939
2.393
Parameter Value
1 Data File Name C:/ Users/ HP/ Desktop/ 22 Post doc research1/ Asmaa anticancer/ NMR/ Ehab AbdElMoneem_H_T-3-indole/ 10/ fid
2 Title Ehab AbdElMoneem_H_T-3-indole.10.fid
3 Origin Bruker BioSpin GmbH
4 Owner nmr
5 Solvent DMSO
6 Temperature 298.0
7 Experiment 1D
8 Number of Scans 32
9 Modification Date 2019-06-24T20:11:19
10 Spectrometer Frequency 400.19
11 Spectral Width 8012.8
12 Lowest Frequency -1535.1
13 Nucleus 1H
14 Spectral Size 65536
15 Absolute Reference
0.93
0.99
0.27
0.67
0.29
0.70
1.01
1.99
0.99
1.99
0.96
1.02
1.00
1.42
0.97
2.02
0.58
3.03
16 15 14 13 12 11 10 9 8 7 6 5 4 3 2 1 0 -1 -2 -3
f1 (ppm)

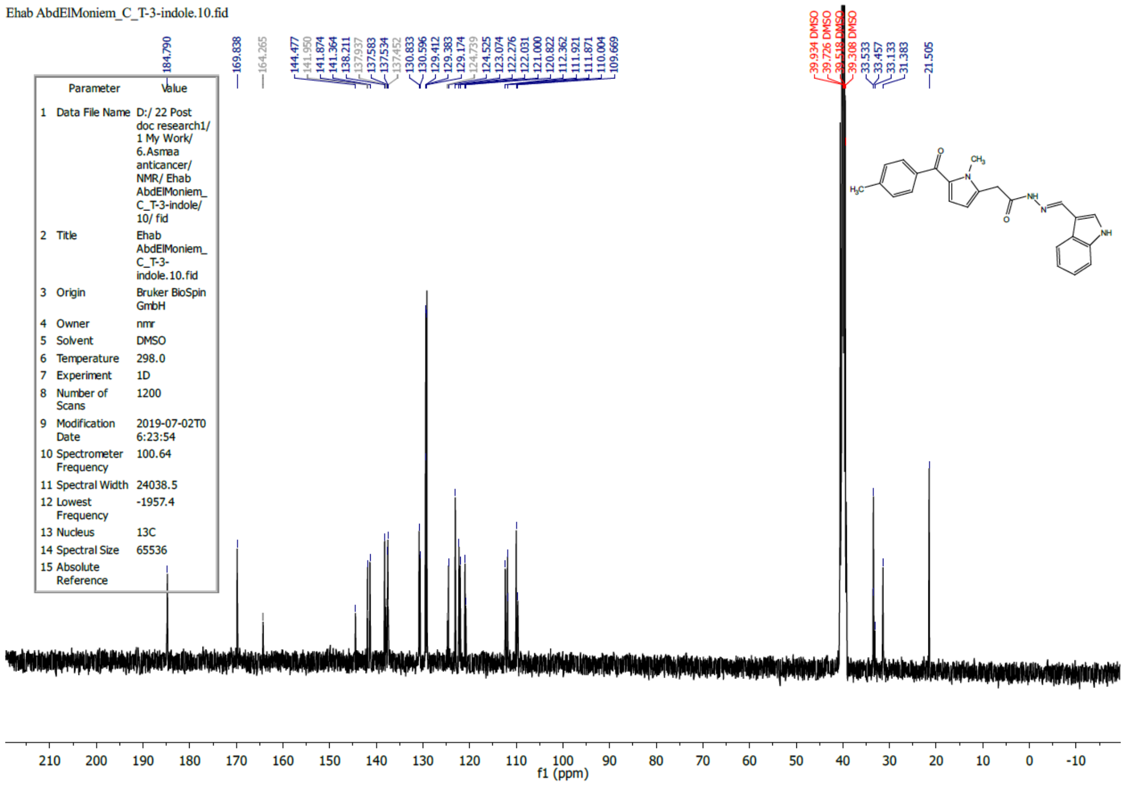

Ehab AbdElMoniem_C_T-3-indole.10.fid
184.790
169.838
164.265
144.477
141.950
141.874
141.364
138.211
137.937
137.583
137.534
137.452
130.833
130.596
129.412
129.383
129.174
124.739
124.525
123.074
122.276
122.031
121.000
120.822
112.362
111.921
111.871
110.004
109.669
39.934 DMSO
39.726 DMSO
39.518 DMSO
39.308 DMSO
33.533
33.457
33.133
31.383
21.505
Parameter Value
1 Data File Name D:/ 22 Post doc research1/ 1 My Work/ 6.Asmaa anticancer/ NMR/ Ehab AbdElMoniem_C_T-3-indole/ 10/ fid
2 Title Ehab AbdElMoniem_C_T-3-indole.10.fid
3 Origin Bruker BioSpin GmbH
4 Owner nmr
5 Solvent DMSO
6 Temperature 298.0
7 Experiment 1D
8 Number of Scans 1200
9 Modification Date 2019-07-02T06:23:54
10 Spectrometer Frequency 100.64
11 Spectral Width 24038.5
12 Lowest Frequency -1957.4
13 Nucleus 13C
14 Spectral Size 65536
15 Absolute Reference
210 200 190 180 170 160 150 140 130 120 110 100 90 80 70 60 50 40 30 20 10 0 -10
f1 (ppm)

Compound 6a

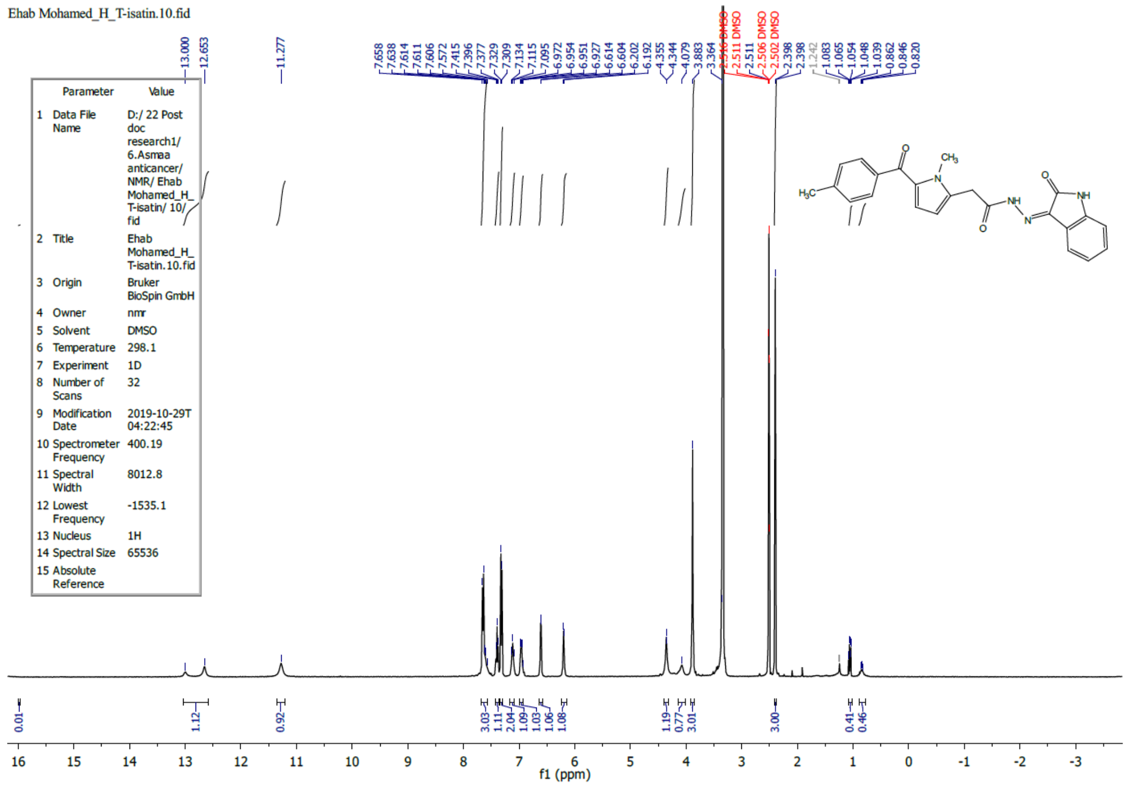
Ehab Mohamed_H_T-isatin.10.fid
f1 (ppm)

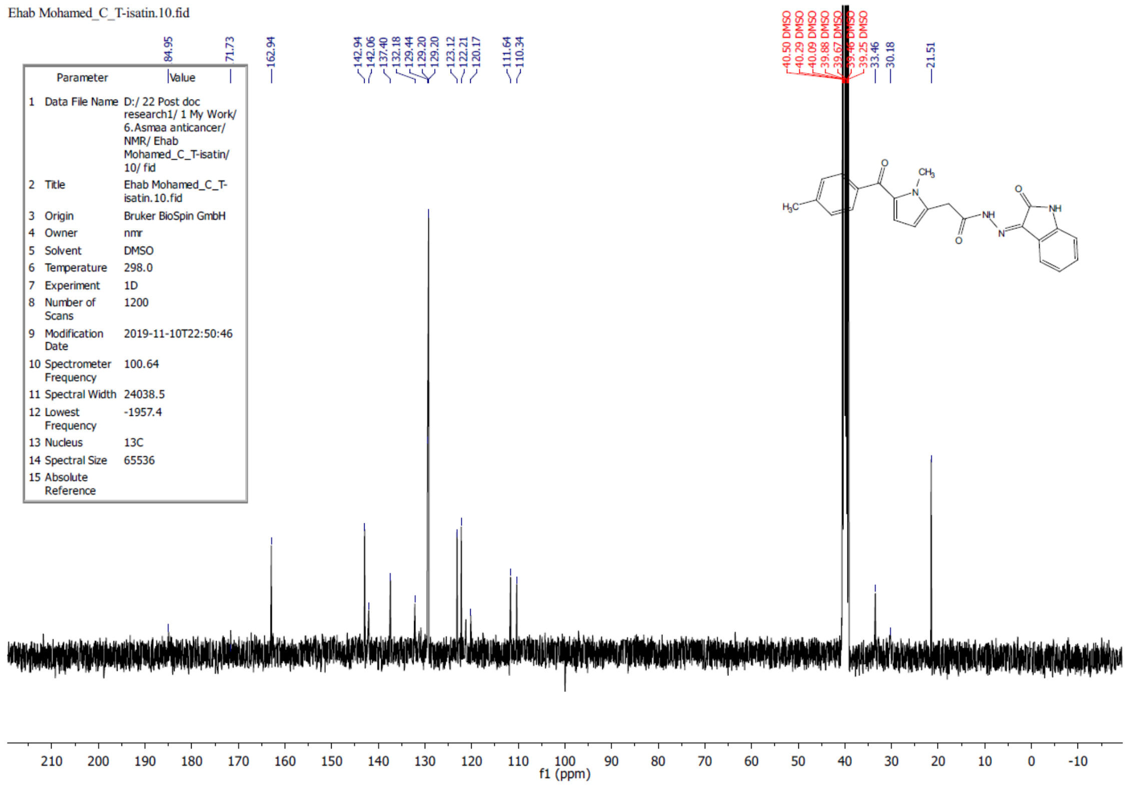
Ehab Mohamed_C_T-isatin.10.fid
f1 (ppm)

Compound 6b

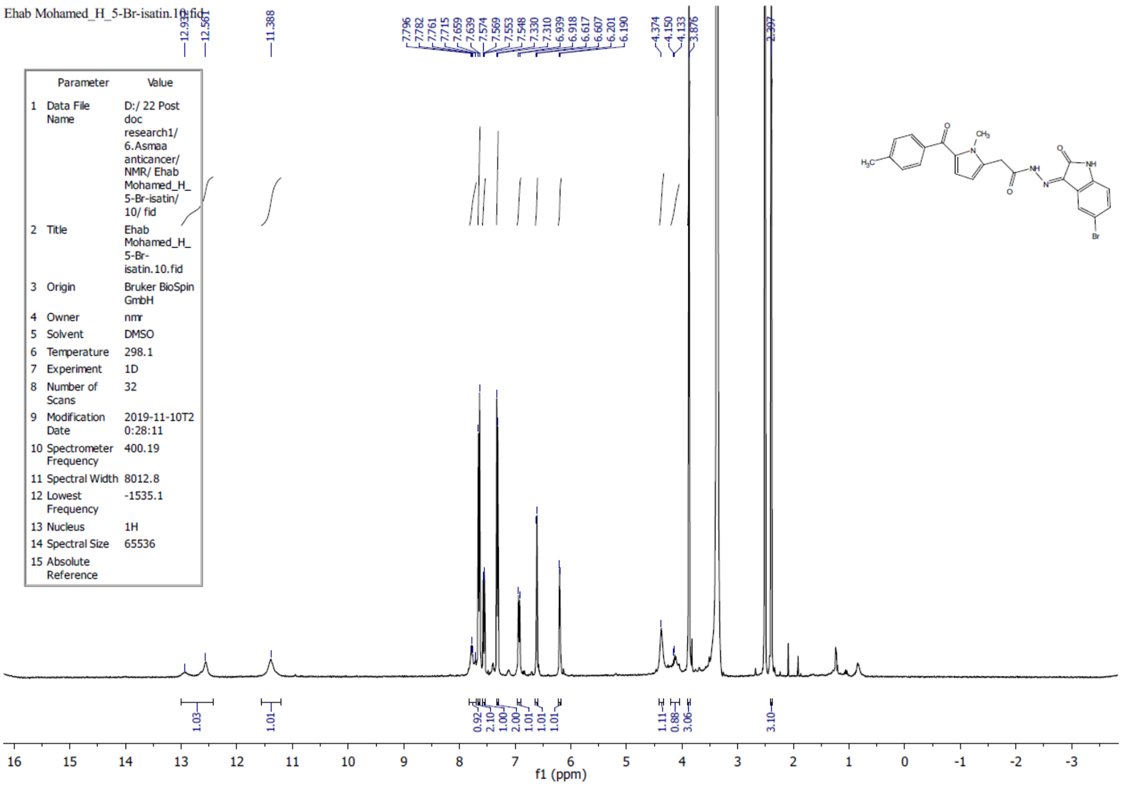

	Parameter	Value
1	Data File Name	D:/ 22 Post doc research1/ 6.Asmaa anticancer/ NMR/ Ehab Mohamed_H_5-Br-isatin/ 10/ fid
2	Title	Ehab Mohamed_H_5-Br-isatin.10.fid
3	Origin	Bruker BioSpin GmbH
4	Owner	nmr
5	Solvent	DMSO
6	Temperature	298.1
7	Experiment	1D
8	Number of Scans	32
9	Modification Date	2019-11-10T20:28:11
10	Spectrometer Frequency	400.19
11	Spectral Width	8012.8
12	Lowest Frequency	-1535.1
13	Nucleus	1H
14	Spectral Size	65536
15	Absolute Reference	

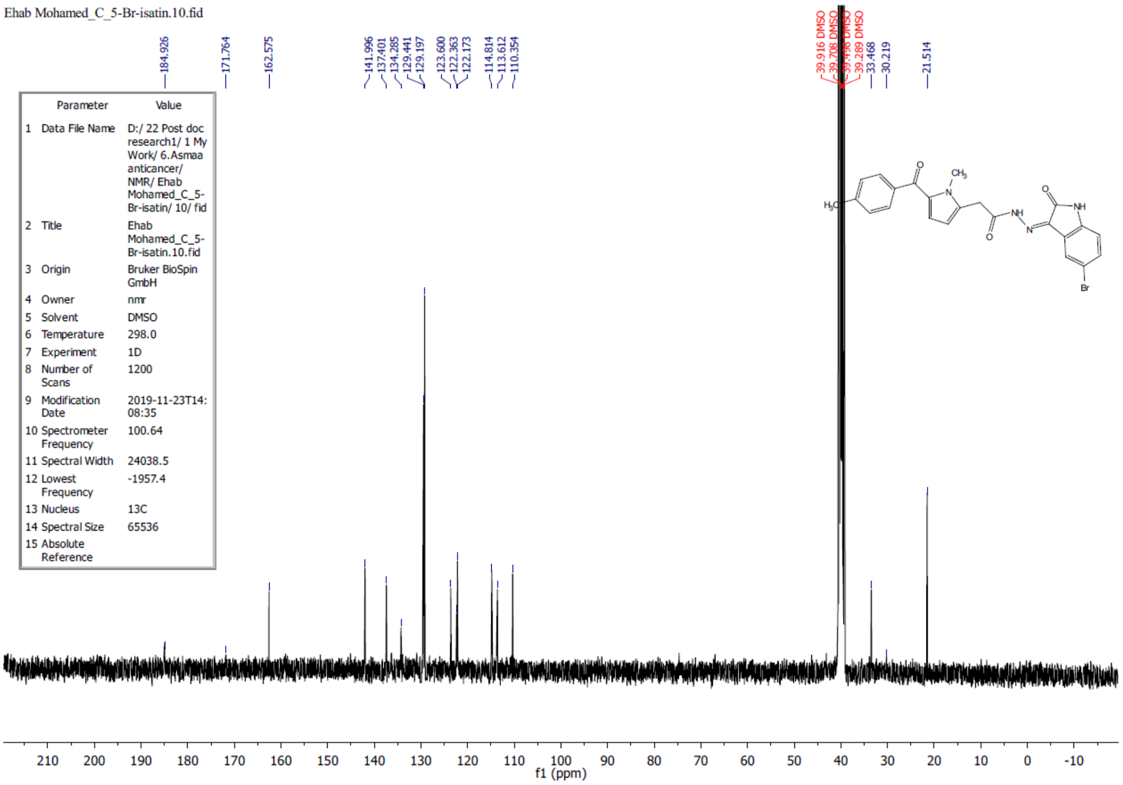

	Parameter	Value
1	Data File Name	D:/ 22 Post doc research1/ 1 My Work/ 6.Asmaa anticancer/ NMR/ Ehab Mohamed_C_5-Br-isatin/ 10/ fid
2	Title	Ehab Mohamed_C_5-Br-isatin.10.fid
3	Origin	Bruker BioSpin GmbH
4	Owner	nmr
5	Solvent	DMSO
6	Temperature	298.0
7	Experiment	1D
8	Number of Scans	1200
9	Modification Date	2019-11-23T14:08:35
10	Spectrometer Frequency	100.64
11	Spectral Width	24038.5
12	Lowest Frequency	-1957.4
13	Nucleus	13C
14	Spectral Size	65536
15	Absolute Reference	

Compound 6c

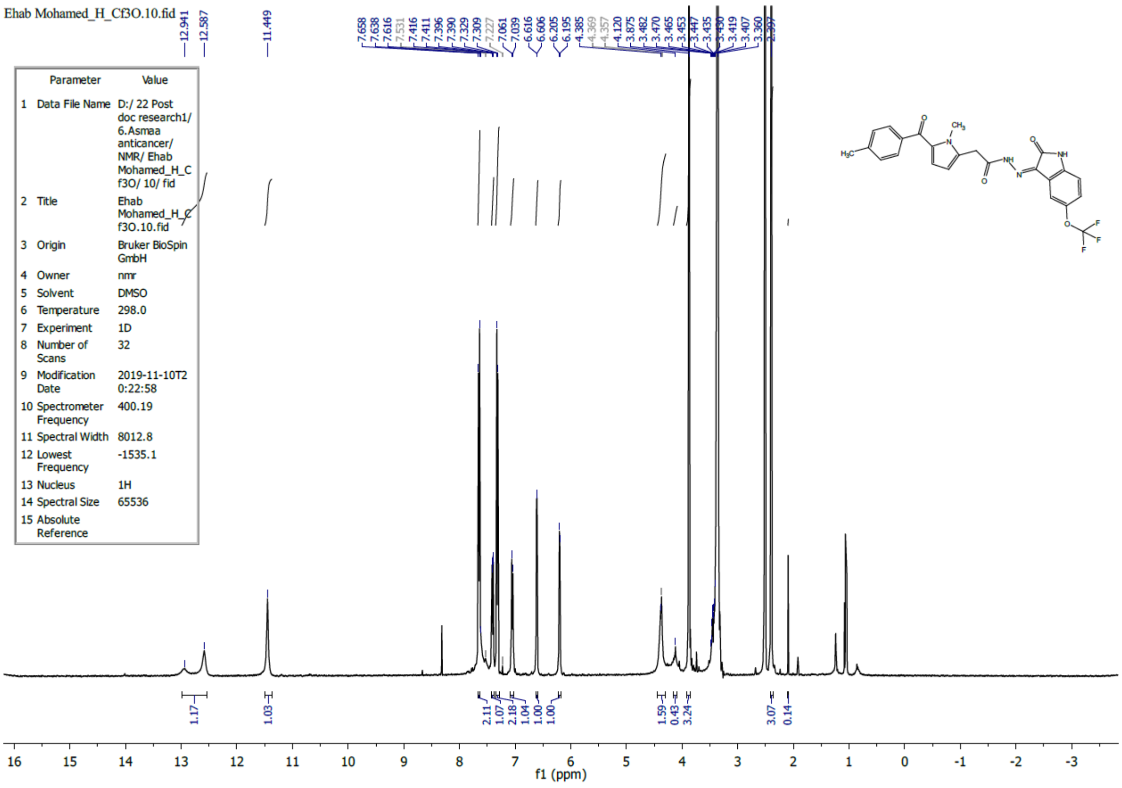

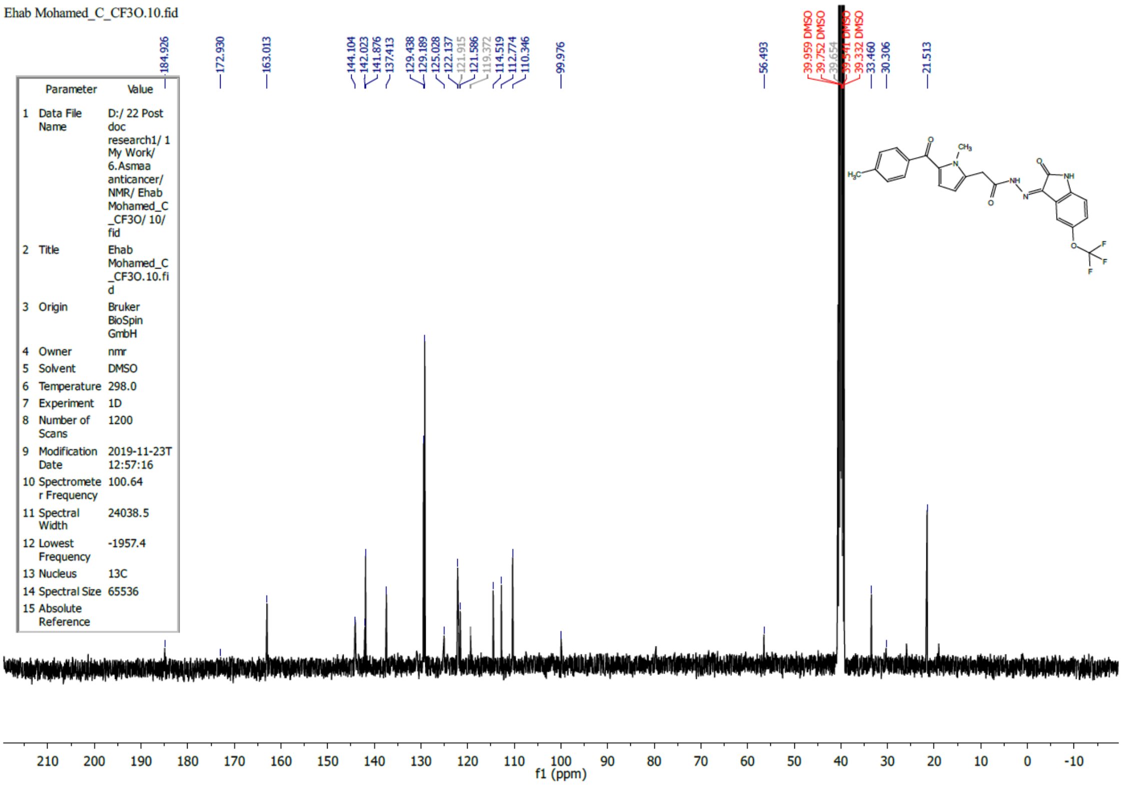

Compound 7a

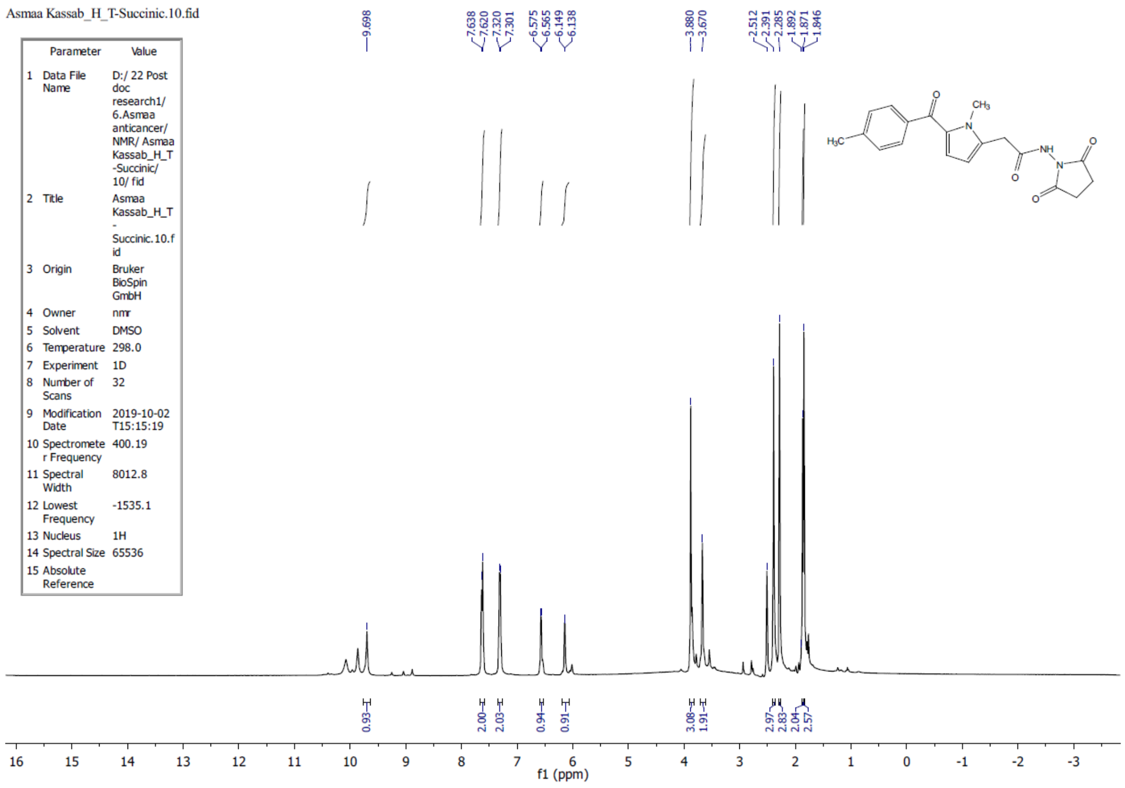
Asmaa Kassab_H_T-Succinic.10.fid
9.698
7.638
7.620
7.320
7.301
6.575
6.565
6.149
6.138
3.880
3.670
2.512
2.391
2.285
1.892
1.871
1.846
Parameter
Value
1 Data File Name
D:/ 22 Post doc research1/ 6.Asmaa anticancer/ NMR/ Asmaa Kassab_H_T -Succinic/ 10/ fid
2 Title
Asmaa Kassab_H_T - Succinic.10.fid
3 Origin
Bruker BioSpin GmbH
4 Owner
nmr
5 Solvent
DMSO
6 Temperature
298.0
7 Experiment
1D
8 Number of Scans
32
9 Modification Date
2019-10-02 T15:15:19
10 Spectrometer Frequency
400.19
11 Spectral Width
8012.8
12 Lowest Frequency
-1535.1
13 Nucleus
1H
14 Spectral Size
65536
15 Absolute Reference
H3C
O
CH3
N
NH
N
O
O
O
0.93
2.00
2.03
0.94
0.91
3.08
1.91
2.97
2.83
2.04
2.57
16
15
14
13
12
11
10
9
8
7
6
5
4
3
2
1
0
-1
-2
-3
f1 (ppm)

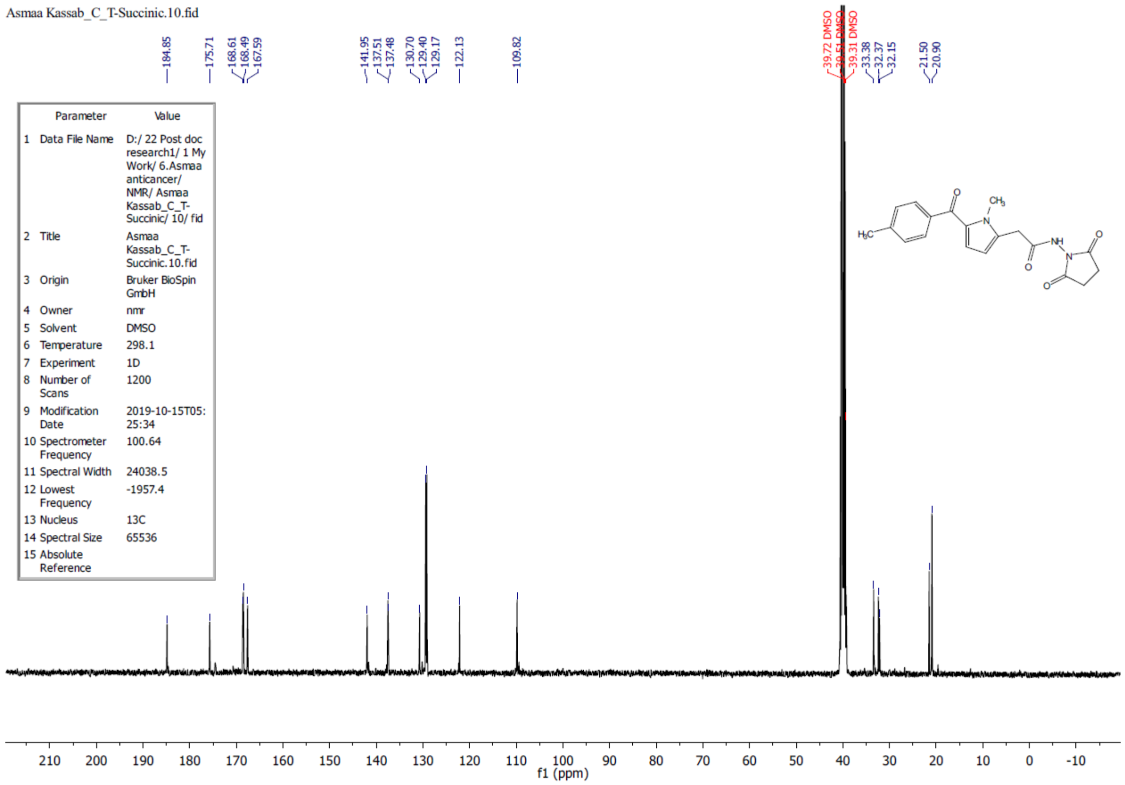
Asmaa Kassab_C_T-Succinic.10.fid
184.85
175.71
168.61
168.49
167.59
141.95
137.51
137.48
130.70
129.40
129.17
122.13
109.82
39.72 DMSO
39.51 DMSO
39.31 DMSO
33.38
32.37
32.15
21.50
20.90
Parameter
Value
1 Data File Name
D:/ 22 Post doc research1/ 1 My Work/ 6.Asmaa anticancer/ NMR/ Asmaa Kassab_C_T-Succinic/ 10/ fid
2 Title
Asmaa Kassab_C_T-Succinic.10.fid
3 Origin
Bruker BioSpin GmbH
4 Owner
nmr
5 Solvent
DMSO
6 Temperature
298.1
7 Experiment
1D
8 Number of Scans
1200
9 Modification Date
2019-10-15T05:25:34
10 Spectrometer Frequency
100.64
11 Spectral Width
24038.5
12 Lowest Frequency
-1957.4
13 Nucleus
13C
14 Spectral Size
65536
15 Absolute Reference
H3C
O
CH3
N
NH
N
O
O
O
210
200
190
180
170
160
150
140
130
120
110
100
90
80
70
60
50
40
30
20
10
0
-10
f1 (ppm)

Compound 7b

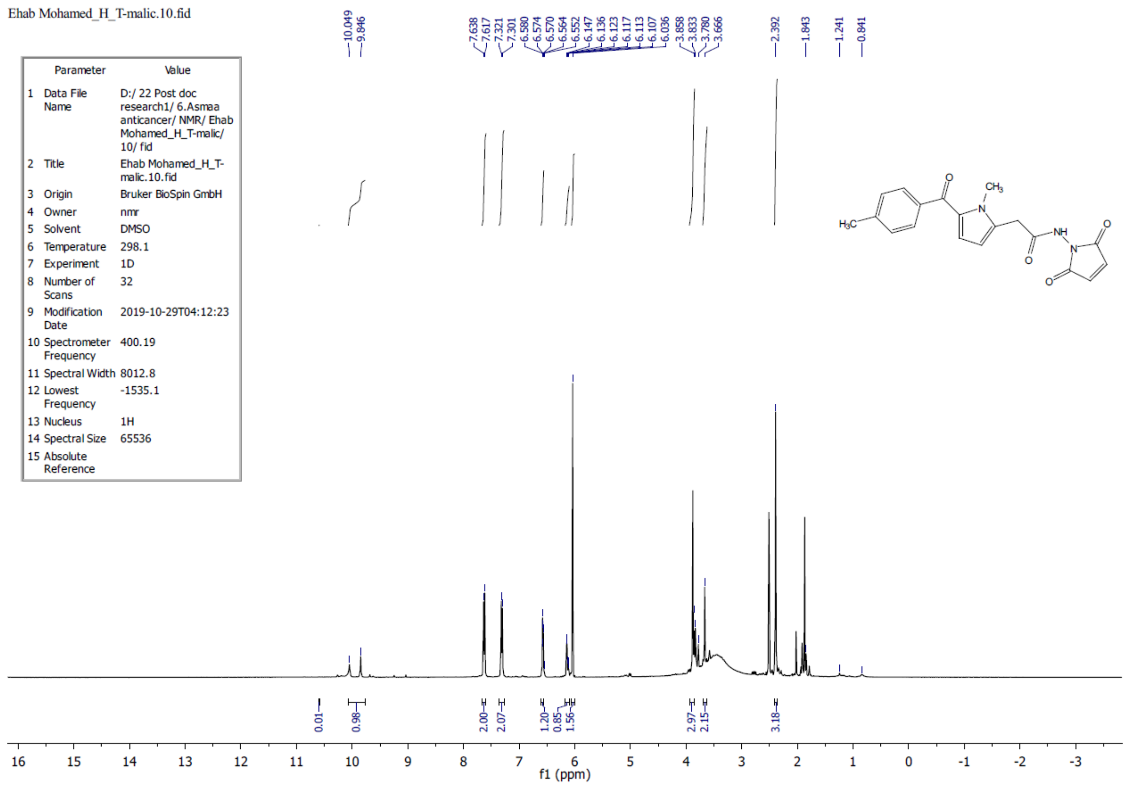

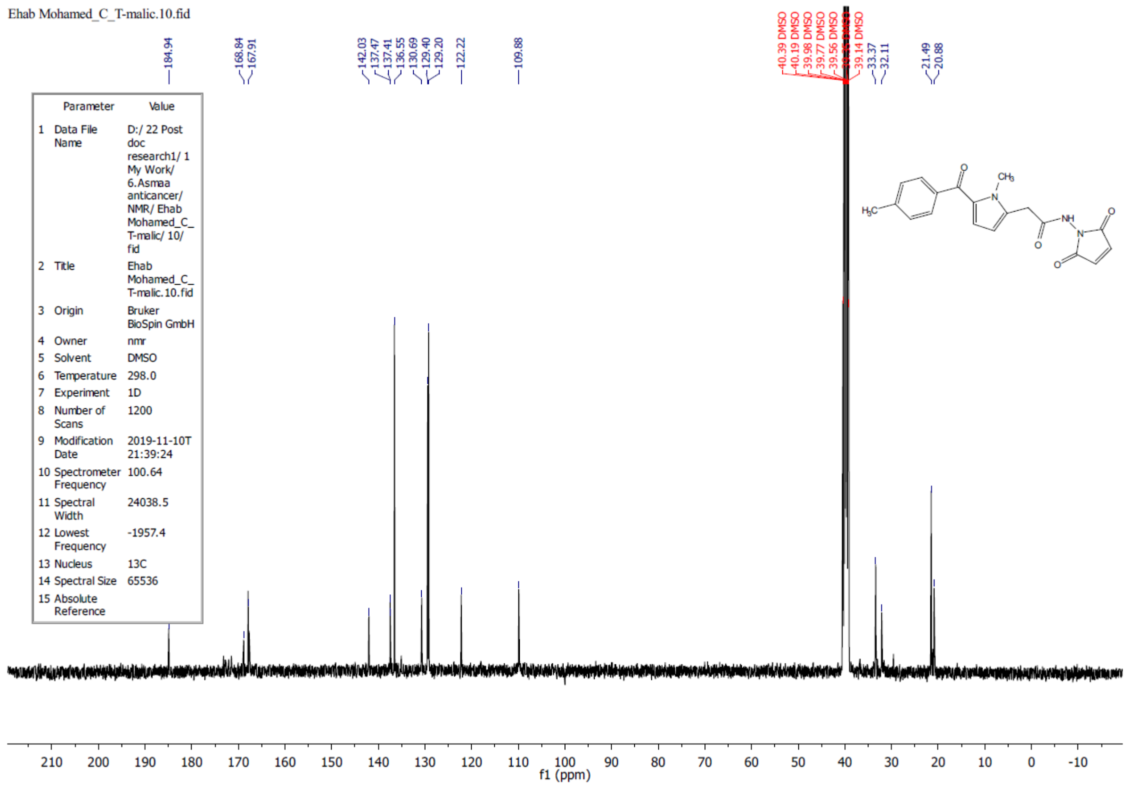

Compound 7c

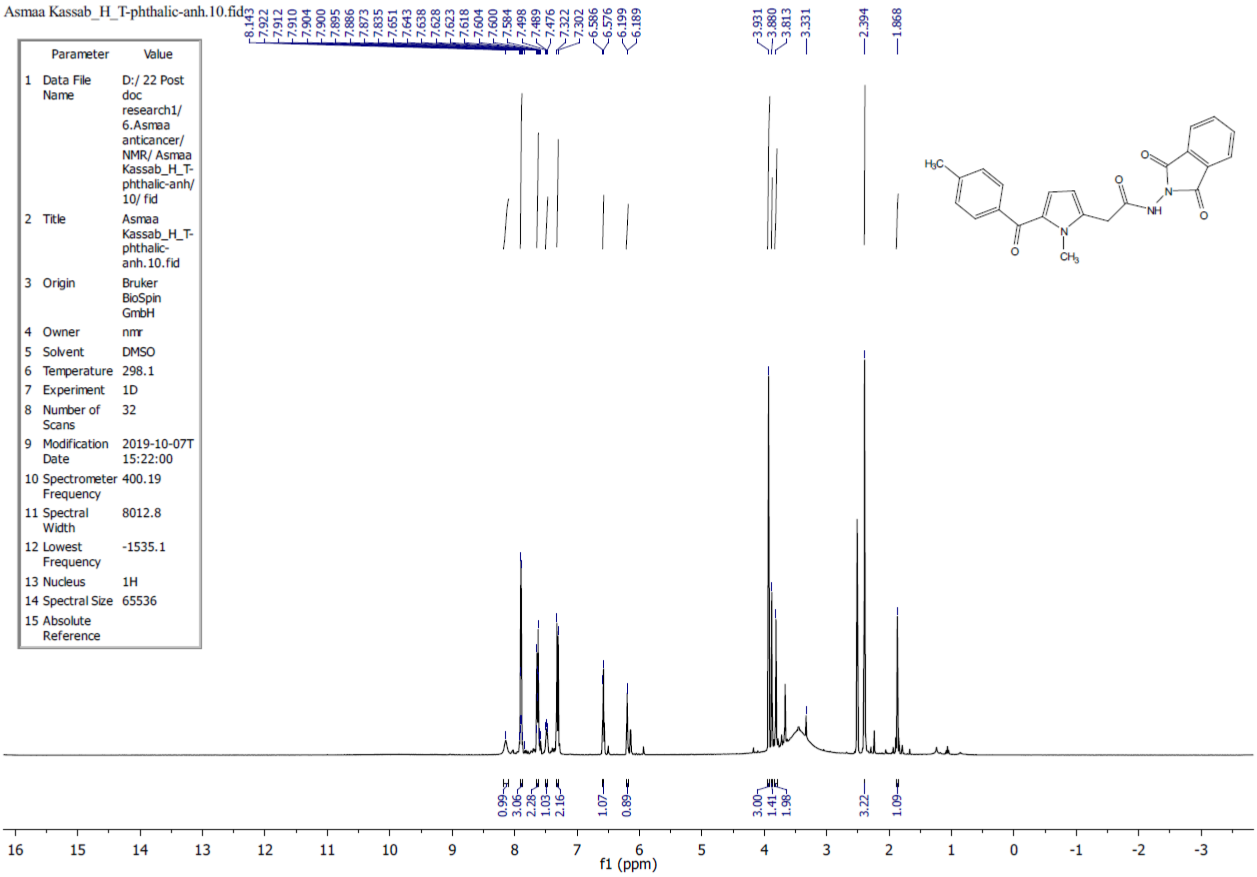

	Parameter	Value
1	Data File Name	D:/ 22 Post doc research1/ 6.Asmaa anticancer/ NMR/ Asmaa Kassab_H_T-phthalic-anh/ 10/ fid
2	Title	Asmaa Kassab_H_T-phthalic-anh.10.fid
3	Origin	Bruker BioSpin GmbH
4	Owner	nmr
5	Solvent	DMSO
6	Temperature	298.1
7	Experiment	1D
8	Number of Scans	32
9	Modification Date	2019-10-07T 15:22:00
10	Spectrometer Frequency	400.19
11	Spectral Width	8012.8
12	Lowest Frequency	-1535.1
13	Nucleus	1H
14	Spectral Size	65536
15	Absolute Reference	

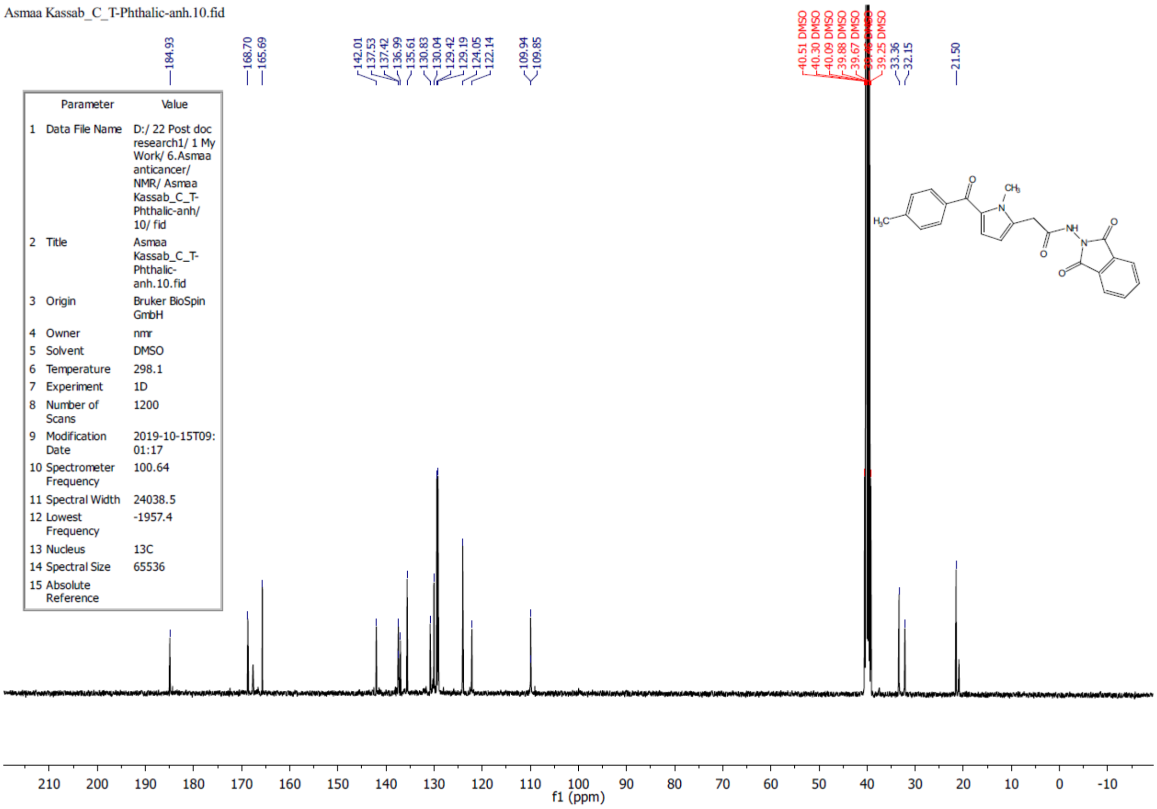

	Parameter	Value
1	Data File Name	D:/ 22 Post doc research1/ 1 My Work/ 6.Asmaa anticancer/ NMR/ Asmaa Kassab_C_T-Phthalic-anh/ 10/ fid
2	Title	Asmaa Kassab_C_T-Phthalic-anh.10.fid
3	Origin	Bruker BioSpin GmbH
4	Owner	nmr
5	Solvent	DMSO
6	Temperature	298.1
7	Experiment	1D
8	Number of Scans	1200
9	Modification Date	2019-10-15T09: 01:17
10	Spectrometer Frequency	100.64
11	Spectral Width	24038.5
12	Lowest Frequency	-1957.4
13	Nucleus	13C
14	Spectral Size	65536
15	Absolute Reference	

Compound 8a

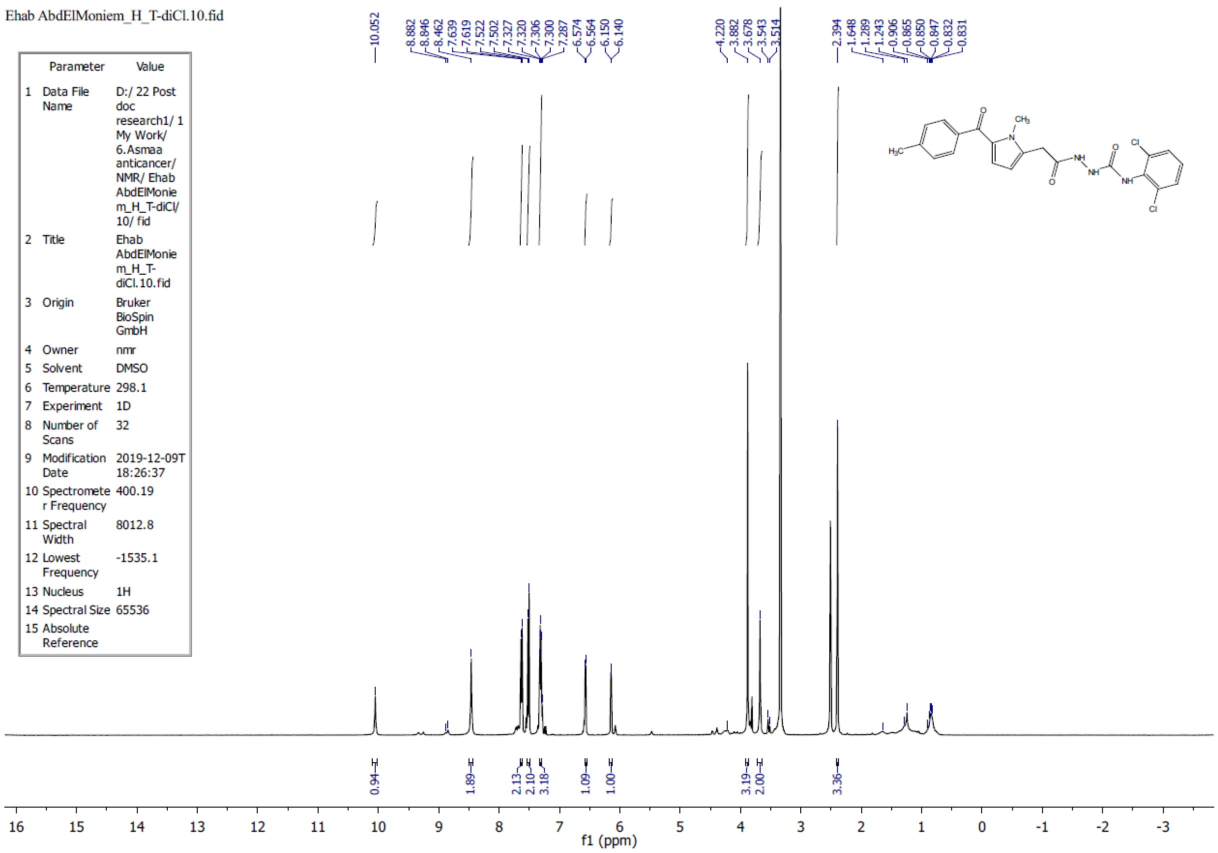

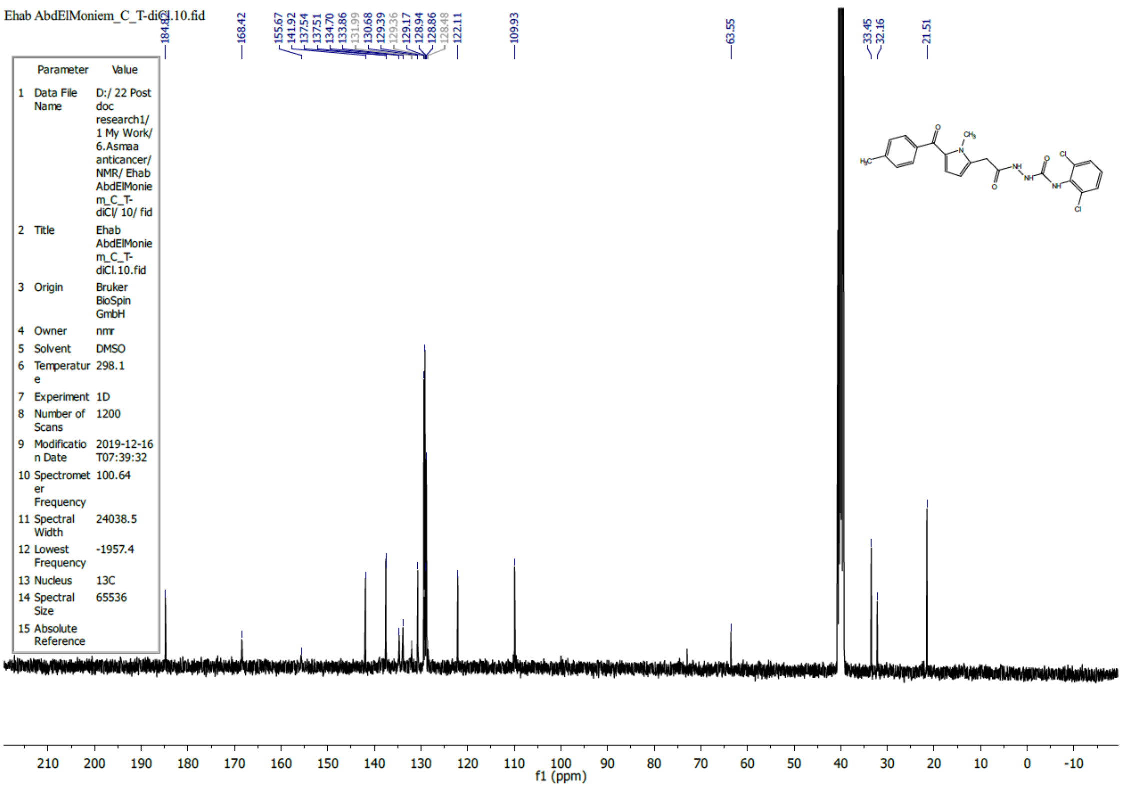

Compound 8b

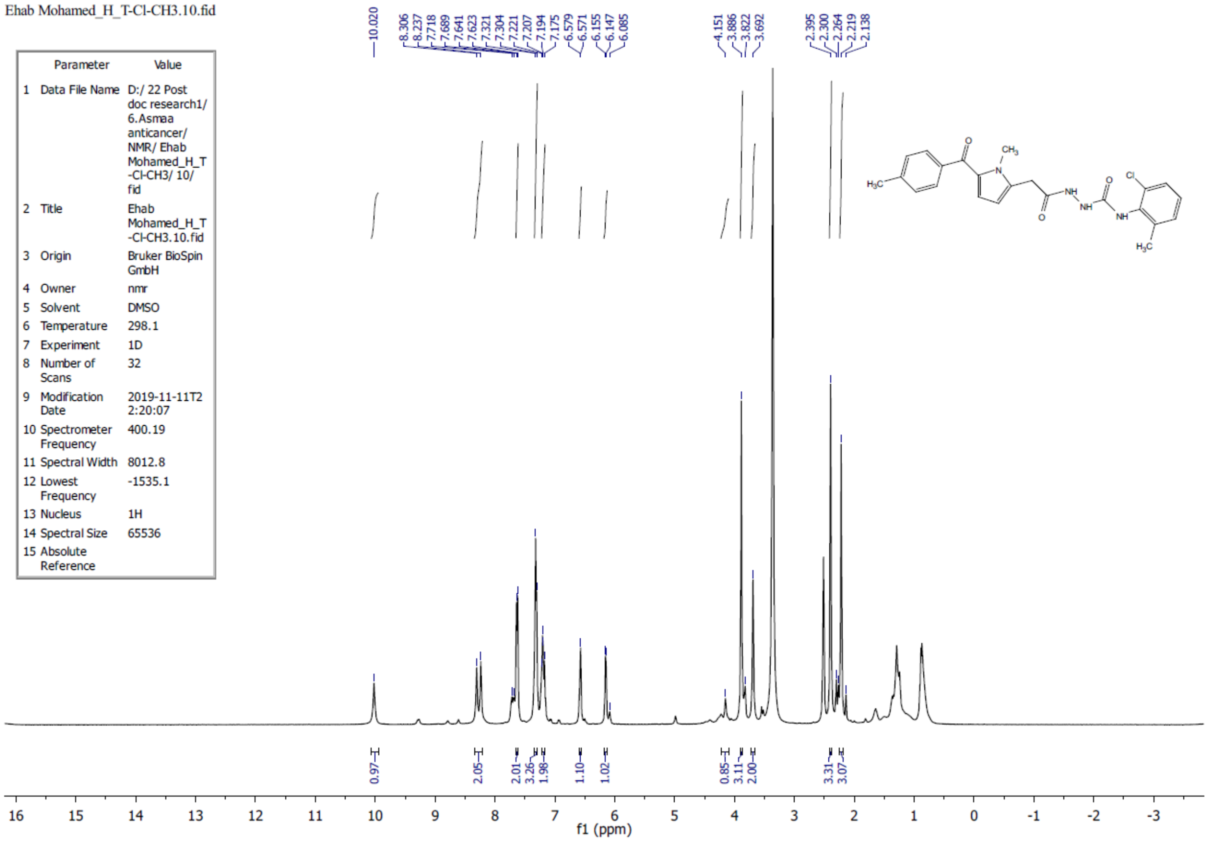
Ehab Mohamed_H_T-Cl-CH3.10.fid
Parameter Value
1 Data File Name D:/ 22 Post doc research1/ 6.Asmaa anticancer/ NMR/ Ehab Mohamed_H_T -Cl-CH3/ 10/ fid
2 Title Ehab Mohamed_H_T -Cl-CH3.10.fid
3 Origin Bruker BioSpin GmbH
4 Owner nmr
5 Solvent DMSO
6 Temperature 298.1
7 Experiment 1D
8 Number of Scans 32
9 Modification Date 2019-11-11T2 2:20:07
10 Spectrometer Frequency 400.19
11 Spectral Width 8012.8
12 Lowest Frequency -1535.1
13 Nucleus 1H
14 Spectral Size 65536
15 Absolute Reference
10.020
8.306 8.237 7.718 7.689 7.641 7.623 7.321 7.304 7.221 7.207 7.194 7.175 6.579 6.571 6.155 6.147 6.085
4.151 3.886 3.822 3.692
2.395 2.300 2.264 2.219 2.138
0.97 2.05 2.01 3.26 1.98 1.10 1.02 0.85 3.11 2.00 3.31 3.07
16 15 14 13 12 11 10 9 8 7 6 5 4 3 2 1 0 -1 -2 -3
f1 (ppm)

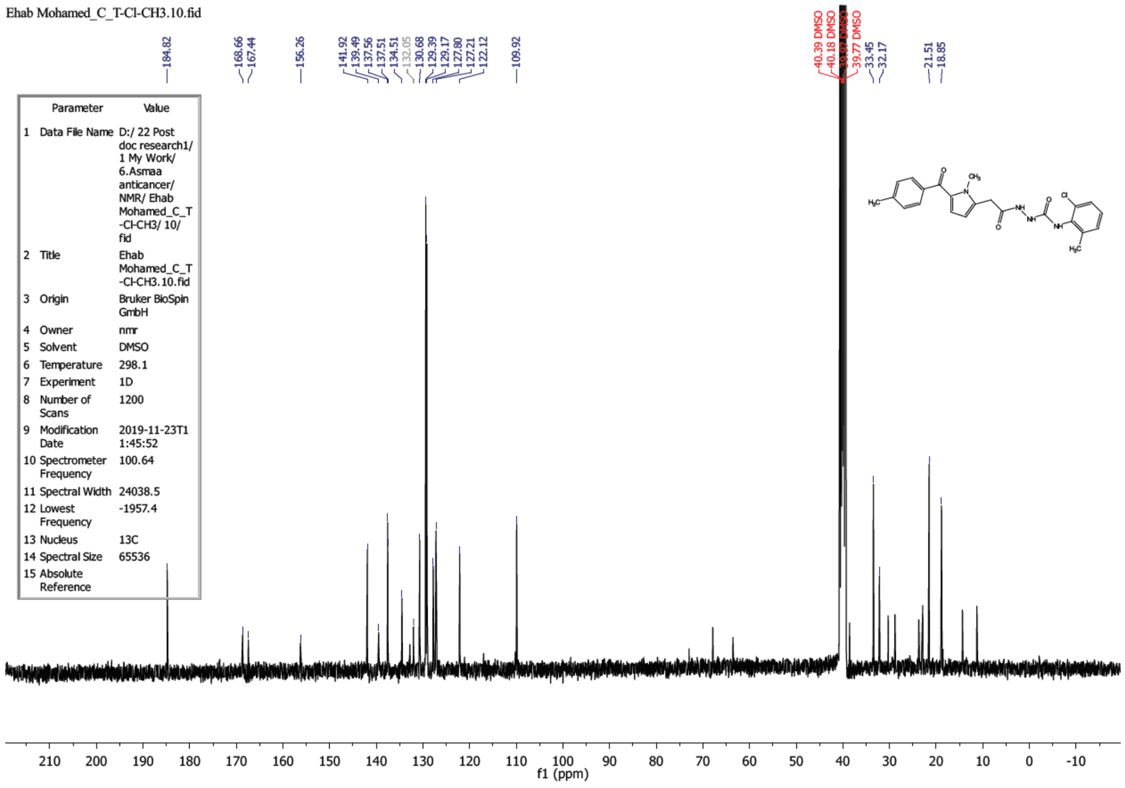
Ehab Mohamed_C_T-Cl-CH3.10.fid
Parameter Value
1 Data File Name D:/ 22 Post doc research1/ 1 My Work/ 6.Asmaa anticancer/ NMR/ Ehab Mohamed_C_T -Cl-CH3/ 10/ fid
2 Title Ehab Mohamed_C_T -Cl-CH3.10.fid
3 Origin Bruker BioSpin GmbH
4 Owner nmr
5 Solvent DMSO
6 Temperature 298.1
7 Experiment 1D
8 Number of Scans 1200
9 Modification Date 2019-11-23T1 1:45:52
10 Spectrometer Frequency 100.64
11 Spectral Width 24038.5
12 Lowest Frequency -1957.4
13 Nucleus 13C
14 Spectral Size 65536
15 Absolute Reference
184.82
168.66 167.44
156.26
141.92 139.49 137.56 137.51 134.51 132.05 130.68 129.39 129.17 127.80 127.21 122.12
109.92
40.39 DMSO 40.18 DMSO 39.77 DMSO
33.45 32.17
21.51 18.85
210 200 190 180 170 160 150 140 130 120 110 100 90 80 70 60 50 40 30 20 10 0 -10
f1 (ppm)

Compound 8c

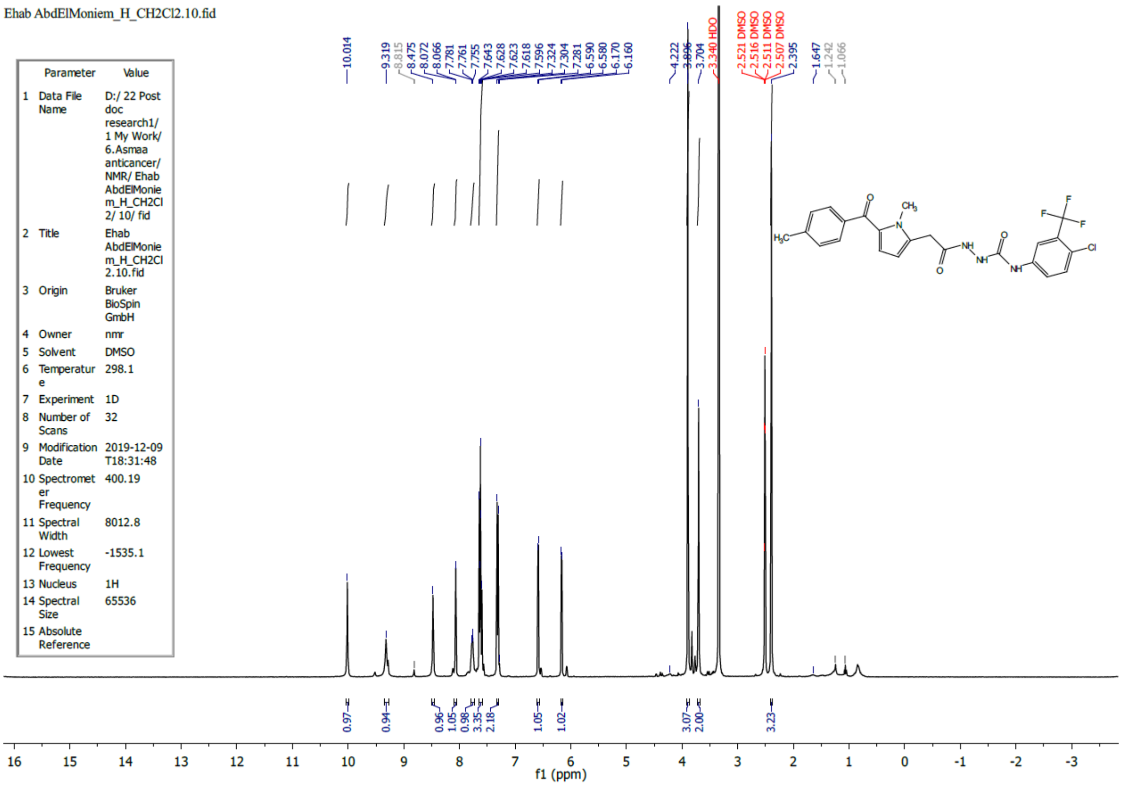
Ehab AbdElMoniem_H_CH2Cl2.10.fid
Parameter Value
1 Data File Name D:/ 22 Post doc research1/ 1 My Work/ 6.Asmaa anticancer/ NMR/ Ehab AbdElMoniem_H_CH2Cl2/ 10/ fid
2 Title Ehab AbdElMoniem_H_CH2Cl2.10.fid
3 Origin Bruker BioSpin GmbH
4 Owner nmr
5 Solvent DMSO
6 Temperature 298.1
7 Experiment 1D
8 Number of Scans 32
9 Modification Date 2019-12-09 T18:31:48
10 Spectrometer Frequency 400.19
11 Spectral Width 8012.8
12 Lowest Frequency -1535.1
13 Nucleus 1H
14 Spectral Size 65536
15 Absolute Reference
10.014
9.319
8.815
8.475
8.072
8.066
7.781
7.761
7.755
7.643
7.628
7.623
7.618
7.596
7.324
7.304
7.281
6.590
6.580
6.170
6.160
4.222
3.896
3.704
3.340 HDO
2.521 DMSO
2.516 DMSO
2.511 DMSO
2.507 DMSO
2.395
1.647
1.242
1.066
0.97
0.94
0.96
1.05
0.98
3.35
2.18
1.05
1.02
3.07
2.00
3.23
f1 (ppm)

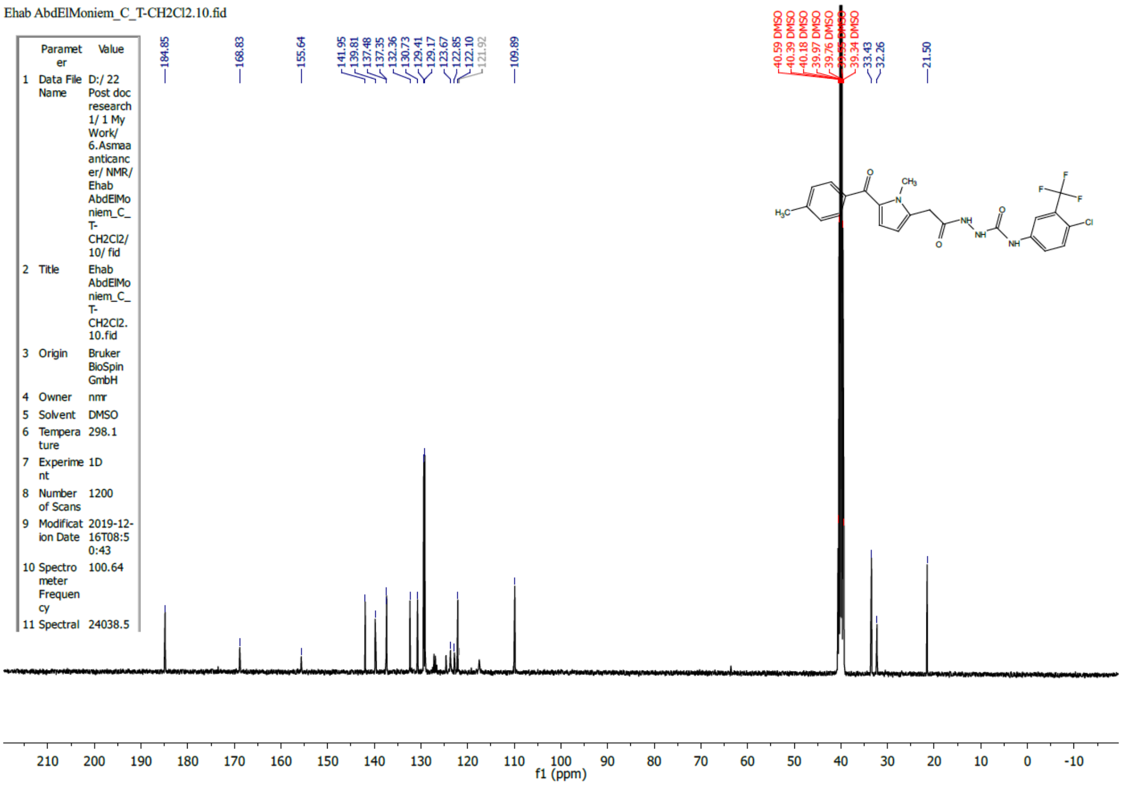
Ehab AbdElMoniem_C_T-CH2Cl2.10.fid
Parameter Value
1 Data File Name D:/ 22 Post doc research1/ 1 My Work/ 6.Asmaa anticancer/ NMR/ Ehab AbdElMoniem_C_T-CH2Cl2/ 10/ fid
2 Title Ehab AbdElMoniem_C_T-CH2Cl2.10.fid
3 Origin Bruker BioSpin GmbH
4 Owner nmr
5 Solvent DMSO
6 Temperature 298.1
7 Experiment 1D
8 Number of Scans 1200
9 Modification Date 2019-12-16T08:50:43
10 Spectrometer Frequency 100.64
11 Spectral 24038.5
184.85
168.83
155.64
141.95
139.81
137.48
137.35
132.36
130.73
129.41
129.17
123.67
122.85
122.10
121.92
109.89
40.59 DMSO
40.39 DMSO
40.18 DMSO
39.97 DMSO
39.76 DMSO
39.34 DMSO
33.43
32.26
21.50
f1 (ppm)
